# A Journey through Diastereomeric Space: The Design, Synthesis, In Vitro and In Vivo Pharmacological Activity, and Molecular Modeling of Novel Potent Diastereomeric MOR Agonists and Antagonists

**DOI:** 10.3390/molecules27196455

**Published:** 2022-09-30

**Authors:** Dana R. Chambers, Agnieszka Sulima, Dan Luo, Thomas E. Prisinzano, Alexander Goldberg, Bing Xie, Lei Shi, Carol A. Paronis, Jack Bergman, Nima Nassehi, Dana E. Selley, Gregory H. Imler, Arthur E. Jacobson, Kenner C. Rice

**Affiliations:** 1 Drug Design and Synthesis Section, Molecular Targets and Medications Discovery Branch, Intramural Research Program, National Institute on Drug Abuse and the National Institute on Alcohol Abuse and Alcoholism, National Institutes of Health, Department of Health and Human Services, 9800 Medical Center Drive, Bethesda, MD 20892, USA; 2 Department of Pharmaceutical Sciences, College of Pharmacy, University of Kentucky, 789 S. Limestone Street, Lexington, KY 40536, USA; 3 Computational Chemistry and Molecular Biophysics Section, Molecular Targets and Medications Discovery Branch, Intramural Research Program, National Institute on Drug Abuse, National Institutes of Health, Department of Health and Human Services, 333 Cassell Drive, Baltimore, MD 21224, USA; 4 McLean Hospital, Harvard Medical School, 115 Mill Street, Belmont, MA 02478, USA; 5 Department of Pharmacology and Toxicology, Virginia Commonwealth University, 1112 East Clay Street, Richmond, VA 23298, USA; 6 Naval Research Laboratory, Center for Biomolecular Science and Engineering, Washington, DC 20375, USA

**Keywords:** diastereomeric C9-alkenyl 5-phenylmorphans, *m*-hydroxy-*N*-phenethyl-5-phenylmorphan, *N*-phenethyl-2-azabicyclo [3.3.1] nonan-5-yl) phenols, MOR, DOR, KOR agonists and antagonists, respiratory depression, molecular modeling and simulation, inactive (4DKL) MOR crystal structures, active (6DDF) MOR crystal structures

## Abstract

Four sets of diastereomeric C9-alkenyl 5-phenylmorphans, varying in the length of the C9-alkenyl chain, were designed to examine the effect of these spatially distinct ligands on opioid receptors. Functional activity was obtained by forskolin-induced cAMP accumulation assays and several compounds were examined in the [^35^S]GTPgS assay and in an assay for respiratory depression. In each of the four sets, similarities and differences were observed dependent on the length of their C9-alkenyl chain and, most importantly, their stereochemistry. Three MOR antagonists were found to be as or more potent than naltrexone and, unlike naltrexone, none had MOR, KOR, or DOR agonist activity. Several potent MOR full agonists were obtained, and, of particular interest partial agonists were found that exhibited less respiratory depression than that caused by morphine. The effect of stereochemistry and the length of the C9-alkenyl chain was also explored using molecular modeling. The MOR antagonists were found to interact with the inactive (4DKL) MOR crystal structures and agonists were found to interact with the active (6DDF) MOR crystal structures. The comparison of their binding modes at the mouse MOR was used to gain insight into the structural basis for their stereochemically induced pharmacological differences.

## 1. Introduction

Opium from the plant *Papaver somniferum* has been used for millennia, and the opioids isolated from the plant have been clinically used for almost two centuries to treat acute and chronic pain. More recently, these opioids and their derivatives have become controversial due to the development of Opioid Use Disorder (OUD) from their use. A serious side effect, respiratory depression, is a major cause of death due to overdose from the misuse of opioids, and constipation and other gastrointestinal (GI) effects can become life-threatening from chronic use of opioids. In addition, tolerance to their analgesic effects develops from their chronic use, necessitating increasing amounts of medication to treat pain. Both licit and illicit use of opioids can lead to physical dependence and OUD. For these reasons, structural modifications and alternatives to the classical opioid morphine-like structures have, over the past century, been designed and synthesized by medicinal chemists at NIH, and in universities and pharmaceutical industry in many different countries [1,2,3]. The synthetic opioids were based initially on the epoxymorphinan and morphinan structures, and eventually both simpler and more complex molecular structures with analgesic activity were derived from them. One of the simplest designed alternative structures was a *m*-hydroxy-*N*-substituted-5-phenylmorphan (*N*-substituted 2-azabicyclo [3.3.1] nonan-5-yl) phenols). These were originally synthesized by May and co-workers starting in 1955 [4], in their successful attempt to find a minimal molecular skeleton based on morphine that would retain antinociceptive activity. These 5-phenylmorphans, unlike the 6,7-benzomorphans and the classical morphinans and epoxmorphinans, have a phenyl ring equatorially, rather than axially oriented towards the piperidine ring of the opioids. The phenylmorphans are, in that respect, not molecularly like the “classical” opioids; the molecule is less rigid than the morphinans and expoxymorphinans.

Analgesics that have less molecular resemblance to the classical opioids have been recently found to have fewer opioid-like side effects [5,6,7,8,9], although the reasons for that are still being debated. One point of view noted that compounds that had fewer opioid-like side effects (e.g., reduced, but not eliminated, respiratory effects) were partial agonists, not fully efficacious in cAMP or [^35^S]GTPgS assays, or in vivo [10]. It has also been noted that G-protein signaling-biased agonists, those that recruit less beta-arrestin on interaction with the μ-opioid receptor (MOR), have fewer side effects, although there has been considerable debate about that theory [9,11]. We formerly examined the side effects of 5-phenylmorphan compounds that did not recruit beta-arrestin [12], and we now explore the effects of those that act as partial agonists. The efficacy of partial agonists that would result in antinociceptives with fewer opioid-like side effects has not been determined. We hoped that with our sets of designed diastereomeric compounds we could gain some insight into the efficacy of partial MOR agonists that might be needed to obtain separation of their antinociceptive activity from their opioid-like side effects. Molecular modeling was employed to examine the various sets of diastereomeric agonists and antagonists for their interaction with the mouse MOR at the molecular level to gain insight into the structural basis for their stereochemically induced pharmacological differences and the mechanism by which they bind to the MOR, using induced-fit docking in combination with MM/GBSA calculations of representative pairs of stereoisomers.

We have synthesized several sets of diastereomers based on the C9-alkenyl-*m*-hydroxy-*N*-phenethyl-5-phenymorphans. For our initial studies, we retained an *N*-phenethyl substituent for all of the new compounds in order to compare our results with those formerly obtained [12]. It is well known that enantiomeric compounds can have totally different pharmacological activity; one enantiomer can be potent and efficacious and the other enantiomer may have little or no effect at MOR (e.g., (−)- and (+)-morphine) [13]. It is less well known whether this extreme difference would be displayed among diastereomers, especially those based on the 5-phenylmorphan molecule. A considerable number of 5-phenylmorphan derivatives have been found to be as or more potent than morphine as an antinociceptive [12,14], but as formerly mentioned, these ligands have marked differences from morphine-like epoxymorphinan structures in the attachment of the aromatic ring.

In our former work with *N*-phenethyl-3-hydroxy-5-phenylmorphans, we noted that a C9-hydroxy, methyl, and a vinyl substituent with specific stereochemistry gave moderately potent, and in some instances extremely potent MOR agonists in vitro and in vivo, and these appeared to be morphine-like in their side effects [14]. We thought that it might be of interest to synthesize diastereomeric sets of compounds with alkenyl substituents at C9 on the 5-phenylmorphan nucleus. Since there are three chiral atoms in these C9-alkenyl substituted molecules and one of them is structurally fixed, we would only need to synthesize four compounds for each C9-alkene moiety (two sets of enantiomers).

## 2. Results and Discussion

### 2.1. Chemistry

**Scheme 1:**
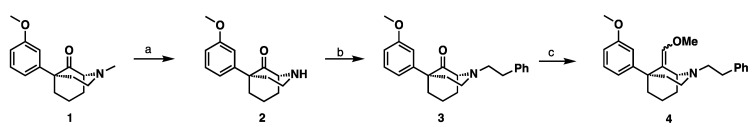
Reagents and Conditions: (**a**) 1. CNBr, K2CO3, MeCN, reflux 4 h, 2. 3 N aq. HCl, MeOH, reflux 16 h, 80%; (**b**) Ph(CH2)2Br, K2CO3, MeCN, reflux 16 h, 77%; (**c**) LiHMDS (methoxymethyl) triphenylphosphonium chloride, THF, 0 °C, 65%.

We sought synthetic pathways for access to the desired two-carbon C9-alkenyl 5-phenylmorphans using the known intermediate **1** [14,15,16]. This intermediate underwent von Braun demethylation to form the secondary amine **2**, followed by alkylation with phenethyl bromide to give intermediate **3** using optimized synthetic procedures [16]. The formation of enol ether **4** was achieved by a Wittig olefination which gave a 1:4 ratio of *E*/*Z* isomers (Scheme 1).

Hydrolysis of methyl vinyl ether **4** with varying concentrations of HCl gave an epimeric mixture of aldehydes (**5**, Table 1). As discussed by Sulima et al., the epimeric mixture of aldehydes **5** was chromatographically unstable; the mixture was used without purification [16].

**Scheme 2:**
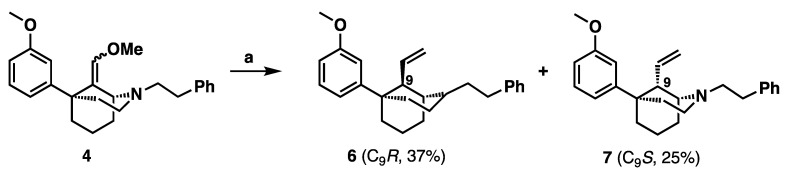
Reagents and Conditions: (**a**) i. HCl, ii. KOtBu, methyltriphenylphosphonium bromide, THF, 45 °C, 3 h.

The 1*R*,5*S*-vinyl (ethenyl) derivatives were synthesized from **4** via the aldehyde intermediate **5** using methyltriphenylphosphonium bromide in a Wittig reaction to yield **6** and **7** (Scheme 2).

**Scheme 3:**
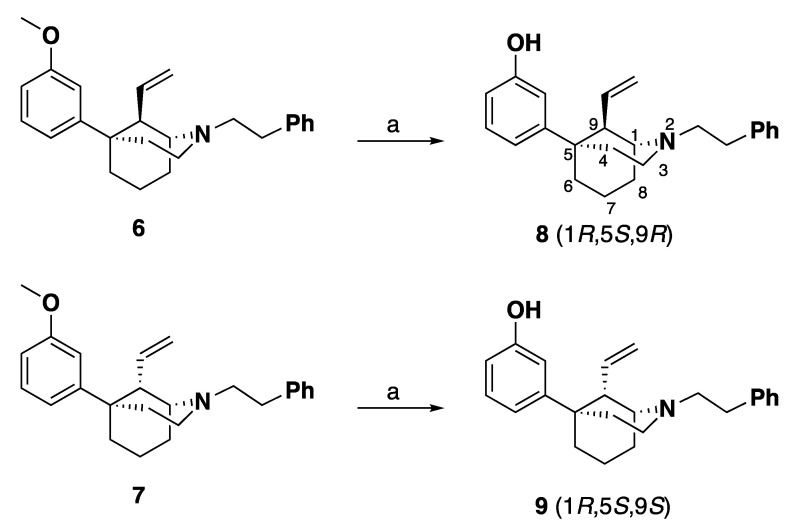
Reagents and Conditions: (**a**) BBr_3_, CH_2_Cl_2_, −78 °C—rt, 4 h, 92%.

The vinyl products **6** and **7** were readily separated by silica gel flash chromatography and the C_9_*R* isomer **6** and the C_9_*S* isomer **7** were subjected O-demethylation to form the phenolic vinyl compounds **8** and **9** (Scheme 3).

**Figure 1:**
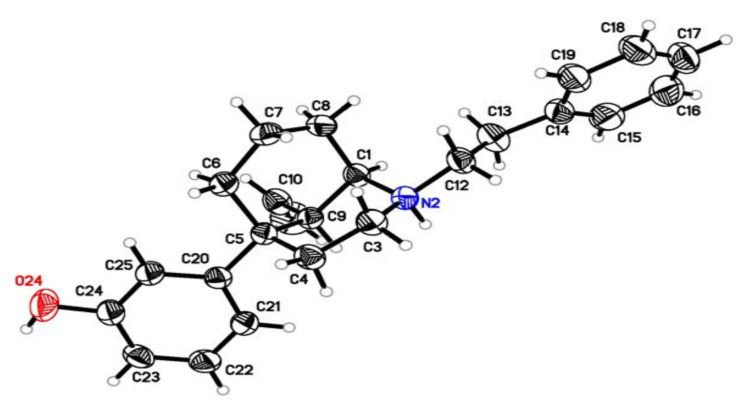
X-ray crystal structure of **8** (3-((1*R*,5*S*,9*R*)-2-phenethyl-9-vinyl-2-azabicyclo [3.3.1] nonan-5-yl) phenol). The ellipsoids are shown at the 50% probability level. Crystal data and atomic coordinates can be found in the Supplementary Materials.

The absolute configuration of **8** (1*R*,5*S*,9*R*) was confirmed by single-crystal X-ray diffraction analysis (Figure 1). Crystal data, atomic coordinates, etc, can be found in the Supplementary Materials.

**Scheme 4:**
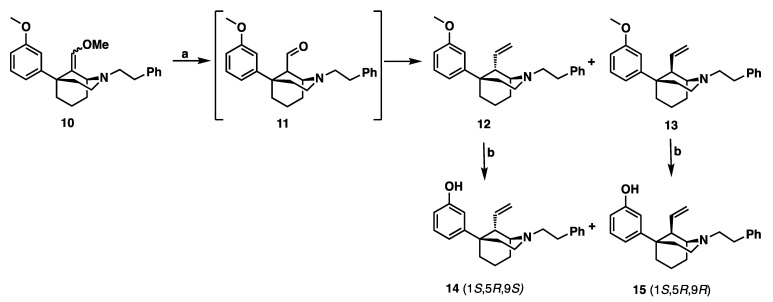
Reagents and Conditions: (**a**) (i). HCl, (ii). KOtBu, methyltriphenylphosphonium bromide, THF, 45 °C, 3 h, 32% **12**: 24% **13**; (**b**) BBr_3_, CH_2_Cl_2_, −78 °C—rt, 4 h, 76%.

The same synthetic route that was used in Scheme 2 and Scheme 3 to synthesize the diastereomers **8** and **9**, was also used to prepare the corresponding 1*S*,5*R* diastereomers **14** and **15**. Using the known 1*S*,5*R*-ketone analogous to **3** [16], the 1*S*,5*R*-aldehyde **11** epimeric mixture was obtained in situ from 1*S*,5*R*-**10**) [12], leading to the phenolic methoxy analogs **12** and **13** which, on *O*-demethylation, gave the desired 1*S*,5*R*-alkenes, the vinyl compounds **14** and **15** (Scheme 4).

**Scheme 5:**
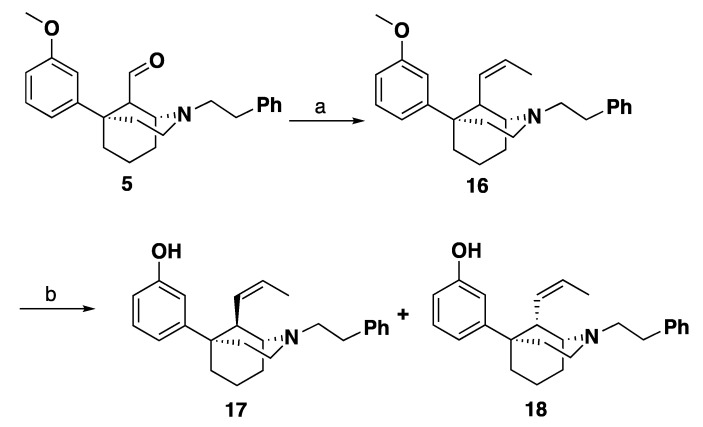
Reagents and Conditions: (**a**) ethyltriphenylphosphonium iodide, THF, LiHMDS, rt, 15 h, 73%; (**b**) BBr_3_, CH_2_Cl_2_, −78 °C—rt, 4 h, 60%.

Synthesis of the C9-propenyl compounds was achieved similarly to the C9-vinyl (ethenyl) products. A Wittig reaction on the unstable aldehyde **5,** formed in situ from **4** using LiHMDS and ethyltriphenylphosphonium iodide, introduced the propylene moiety to the C9 position (Scheme 5). The Wittig products formed were an epimeric mixture (**16**) that was not easily separable by column chromatography. The mixture **16** was subjected to the standard *O*-demethylation conditions to yield the phenolic 1*R*,5*S*-propylene compounds **17** and **18** which were easily separable.

**Scheme 6:**
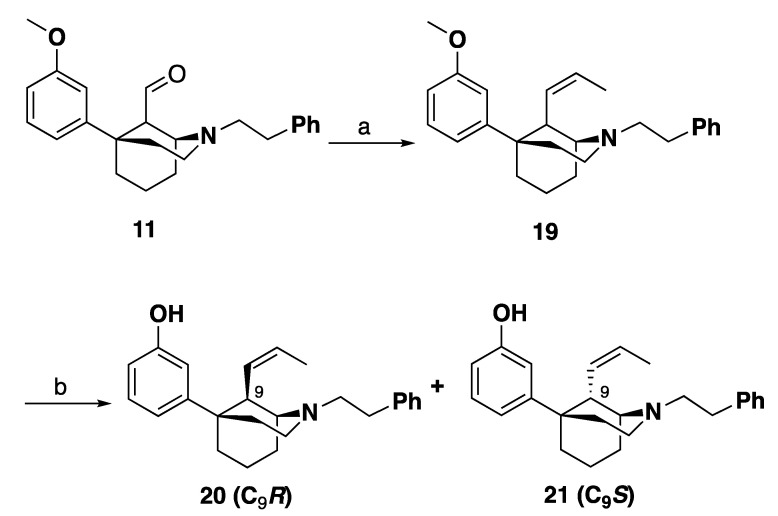
Reagents and Conditions: (**a**) ethyltriphenylphosphonium iodide, THF, LiHMDS, rt, 15 h, 43%; (**b**) BBr_3_, CH_2_Cl_2_, −78 °C—rt, 4 h, 94%.

The same synthetic steps that were performed on the 1*R*,5*S*-phenylmorphans (Scheme 5) were used to obtain the corresponding 1*S,*5*R*-C9-propylene target compounds **20** and **21** (Scheme 6).

**Scheme 7:**
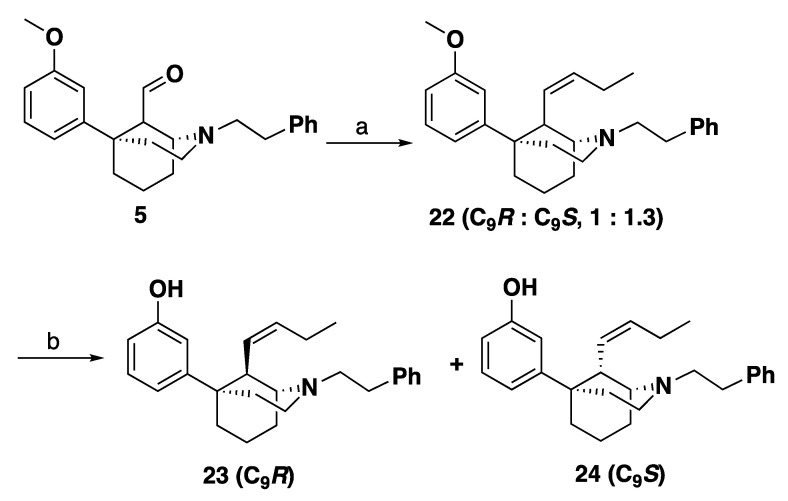
Reagents and Conditions: (**a**) propyltriphenylphosphonium bromide, THF, LiHMDS, 45 °C 15 h, 70%; (**b**) BBr_3_, CH_2_Cl_2_, −78 °C—rt, 4 h, 60%.

As with the other alkenes, the C9-butylene compounds **23** and **24** were synthesized from the aldehyde intermediate **5**, obtained in situ from **4** using propyltriphenylphosphinium bromide with LiHMDS as the base (Scheme 7). This Wittig reaction required heating at 45 °C for 15 h for consumption of starting material. The extended reaction times and heat resulted in more of the C9*R* epimer **23** to form from this reaction compared to the C9*S.*epimer **24**. This ratio was observed by ^1^H-NMR as these methoxy compounds were not easily separable by column chromatography. The mixture of epimers underwent *O*-demethylation using BBr_3_ at which point the epimers could be separated.

**Scheme 8:**
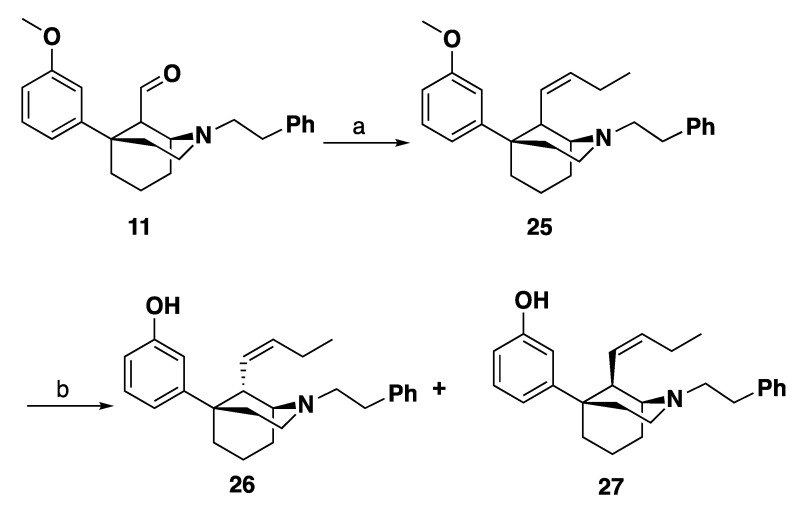
Reagents and Conditions: (**a**) propyltriphenylphosphonium bromide, THF, LiHMDS, 45 °C 15 h, 53%; (**b**) BBr_3_, CH_2_Cl_2_, −78 °C—rt, 4 h, 61%.

The same conditions were used to synthesize the C9-butylene phenolic compounds **26** and **27** (Scheme 8).

**Figure 2:**
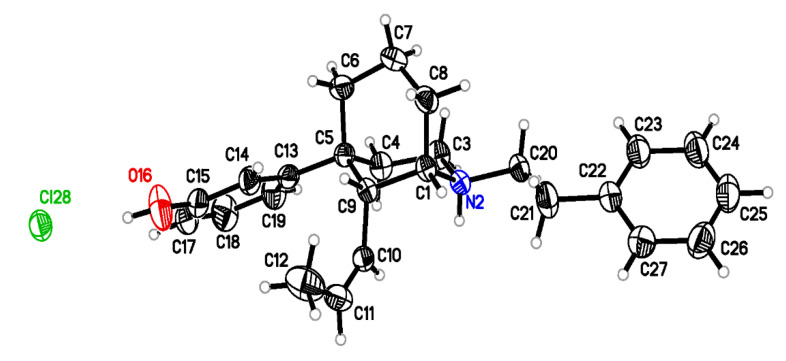
X-ray crystal structure of **20** (3-((1*S*,5*R*,9*R*)-2-phenethyl-9-((*Z*)-prop-1-en-1-yl)-2-azabicyclo [3.3.1] nonan-5-yl) phenol). The ellipsoids are shown at the 50% probability level. Crystal data and atomic coordinates can be found in the Supplementary Materials.

The propylene and the butylene series of compounds were all isolated as the *Z*-isomer, as indicated by the X-ray crystal structure of the propylene diastereomer **20** (Figure 2). The NMR pattern in the ca. d 5.7–5.3 region was similar for all of the Z-isomers, with an observed ca. 10.6 coupling constant typical of Z isomers, in several of the diastereomers that were not unresolved multiplets.

### 2.2. In Vitro Studies

#### 2.2.1. Ligand Efficacy and Potency (Forskolin-Induced cAMP Accumulation Assay)

The functional activity, as determined from the forskolin-induced cAMP accumulation assay, of three sets of compounds, each containing four diastereomers, can be seen in Table 2. The sets differed in the length of the alkenyl moiety at C9 and the compounds within each of the sets differed only in their stereochemistry. The vinyl compounds in the first set had disparate activities. Diastereomer **15** with 1*S*,5*R*,9*R* stereochemistry was a potent MOR antagonist, more than twice as potent as naltrexone IC_50_ = 3.58 nM vs. naltrexone IC_50_ = 10.78). It had some DOR (IC_50_ = 143.7 nM) and KOR (IC_50_ = 28.4 nM) antagonist activity and was devoid of agonist activity at MOR, DOR, and KOR. The other three diastereomers in that set were morphine-like agonists in potency at MOR, two of them, 1*R*,5*S*,9*S*-**9** and its diastereomer 1*S*,5*R*,9*S*-**14**, were partial agonists with moderate or low efficacy (%E_max_ = 67.3 and 46.6, respectively), and the third, 1*R*,5*S*,9*R*-**8** was a fully efficacious agonist in the cAMP assay. The 1*S*,5*R*,9*R* stereochemistry of **15** pertained to **20** in the second set, and in the third set to **27**. These 1*S*,5*R*,9*R* compounds acted as potent MOR antagonists, and variably potent DOR and KOR antagonists. None of them had MOR, DOR or KOR agonist activity in the cAMP assay.

The propenyl diastereomers in the second set were also quite different and, as observed in the first set, contained a potent antagonist **20** (IC_50_ = 2.34 nM), and three MOR agonists. The agonist **18** with 1R,5S,9S stereochemistry had subnanomolar potency at MOR (EC_50_ = 0.07 nM) in the cAMP assay. It was 89 times more potent than morphine, and it had some DOR partial agonist activity (EC_50_ = 9.69 nM, %E_max_= 74.5) and KOR antagonist activity with low potency (IC_50_ = 237.7 nM) (Table 2). The other two compounds in this set were MOR partial agonists, **17** and **21**, had morphine-like potency (EC_50_ = 2.61 and 4.66 nM). We considered **17** as worthy of further examination since it appeared, based on our previous work [12], to have efficacy that might be in the range (%E_max_ < 90 and >65 in the cAMP assay) that we hypothesized might be necessary for a morphine-like antinociceptive with reduced side effects.

The third set of diastereomers had a C9-butenyl substituent and it contained two antagonists, **26** and **27.** Diastereomer **27** had the anticipated 1*S*,5*R*,9*R* stereochemistry; it was twice as potent as naltrexone at MOR, with modest DOR antagonist activity and subnanomolar potency as a KOR antagonist. The 1*S*,5*R*,9S diastereomer **26** was a weak MOR antagonist. The two remaining diastereomers in that set (**23** and **24**) had relatively weak MOR potency. The agonist potency, but not the antagonist potency, decreased with increased bulk at C9.

The importance of a phenolic hydroxyl can be seen in the inactivity of the methoxy analog **13**. Apparently, that phenolic hydroxyl is essential for interaction with opioid receptors with the 5-phenylmorphans.

The cAMP functional assay clearly showed major differences in activity between the diastereomers within a set of four compounds, and differences between the diastereomers in each of the three sets. The vinyl (**15**), propenyl (**20**), and butenyl (**27**) diastereomers with the same 1*S*,5*R*,9*R* stereochemistry had the same activity, they were all MOR, DOR, KOR antagonists with varying potencies, and all three were more potent than naltrexone. Unlike naltrexone, none of them had KOR agonist activity. These antagonists might be better able to antagonize the in vivo effects of more potent narcotics such as fentanyl and etonitazene. Two or three partial agonists, the 1*R*,5*S*,9*S*-vinyl diastereomer (**9**) and the 1*R*,5*S*,9*R*-propenyl diastereomer (**17**) appeared to have sufficient efficacy to warrant further examination for their in vivo activity. The 1*S*,5*R*,9S- propenyl diastereomer (**21**) appeared, in theory, to have marginal efficacy for antinociceptive activity in vivo.

#### 2.2.2. Opioid Receptor Binding, Ligand Efficacy and Potency ([^35^S]GTPgS Functional Assay)

The binding affinities at the MOR and functional activity in the [^35^S]GTPgS assay (Table 3) were determined for two of the diastereomers in the first set of C9-vinyl compounds (**8** and **9**), and two from the second set of C9-propenyl diastereomers (**17** and **18**). These were chosen to compare the efficacies of full agonists (**8** and **18**) and partial agonists (**9** and **17**) in the cAMP assay to those in the GTP assay.

The two vinyl compounds **8** and **9** had high MOR affinity in the binding assay, with **8** showing subnanomolar MOR affinity (Table 3). The two propylene compounds **17** and **18** also had high MOR affinity, and **18** had subnanomolar MOR affinity. The diastereomeric compounds **8** and **18** were more potent than **9** and **17** in the [^35^S]GTPgS functional assay, in accordance with the binding assay. The rank order of the potencies for the four compounds in Table 3 in the [^35^S]GTPgS assay were in agreement with the data from the MOR binding assay. All four compounds were more potent than morphine. All of the compounds, with the exception of **18** which was morphine-like in efficacy, appeared to be partial agonists in the [^35^S]GTPgS assay. The lower potency and efficacy of compounds in the [^35^S]GTPgS assay, as compared with the cAMP assay, was expected due to the inherent differences between these assays [18]. The efficacies of **9** and **17** in both the cAMP (Table 2) and [^35^S]GTPgS (Table 3) assays were both in the range (efficacies < 20% and >10% in the [^35^S]GTPgS assay) that we wanted to explore to determine if those partial agonists might have fewer opioid-like side effects, as postulated by theory [19].

The efficacies of the compounds in the two functional assays were somewhat different in that in the cAMP assay both **8** and **18** appeared to be morphine-like agonists. Both functional assays indicated that **9** and 1**7** were partial agonists. If the activity of these compounds were only related to their stereochemistry, **9** and **18** with 1*R*,5*S*,9*S* stereochemistry should show similarities in MOR affinity, potency and/or efficacy. They did not appear to have that relationship. Unlike that pair, the other two stereochemically similar compounds, **8** and **17**, both with 1*R*,5*S*,9*R* stereochemistry, did appear to be somewhat similar in both the cAMP assay and in the GTP assay, although a difference was seen in their MOR binding affinity. The alkenes with 1*S*,5*R*,9*R* stereochemistry were not agonists. In all three sets of diasteromers that stereochemistry gave potent MOR antagonists. Obviously, within a set of C9-diastereomeric compounds, stereochemistry must be the dominant factor for their functional activity since all four compounds in a set had exactly the same 2-dimensional structure. Several compounds in the sets of diastereomers were examined by molecular modeling in an attempt to determine the differences between the C9-alkene MOR agonists and antagonists at the receptor level.

#### 2.2.3. Molecular Modeling Results and Discussion

From experimental results, significant functional differences were observed between stereoisomers. To understand the structural basis of these differences and the mechanism by which they bind to the MOR, we carried out induced-fit docking in combination with MM/GBSA calculations of representative pairs of stereoisomers. We first focused on two vinyl pairs in the (1*R*,5*S*) series, the 9*R*-vinyl **8** and 9*S*-vinyl **9**, and in the (1*S*,5*R*) series, 9*R*-vinyl **15** and 9*S*-vinyl **14** which are four diastereomers with the same C9-vinyl substituent in the 3-hydroxy-5-phenylmorphans.

##### Accommodation of the C9-Vinyl MOR Agonists 8, 9, and 14 in Both Active and Inactive Conformations of the MOR

Common to both inactive and active MOR models, our docking results of pairs of vinyl analogs show that their protonated and positively charged nitrogen of the 5-phenylmorphan forms an ionic interaction with D147^3.32^ (superscripts denote Ballesteros–Weinstein numbering [20], the *N*-phenethyl is oriented towards the intracellular side of the binding pocket, and the phenol points towards the extracellular vestibule of the transmembrane domain. However, by comparing the inactive (4DKL) and active (6DDF) MOR crystal structures, we found that the side-chain orientation of N150^3.35^ differs between the structures, which results in different shapes of the binding pocket in the active and inactive states. In the inactive structure 4DKL, N150^3.35^ protrudes into and occludes part of the binding site that is otherwise unblocked in the active structure 6DDF. This structural difference of the binding site causes the docked ligands to adopt different binding modes in each receptor.

**Figure 3:**
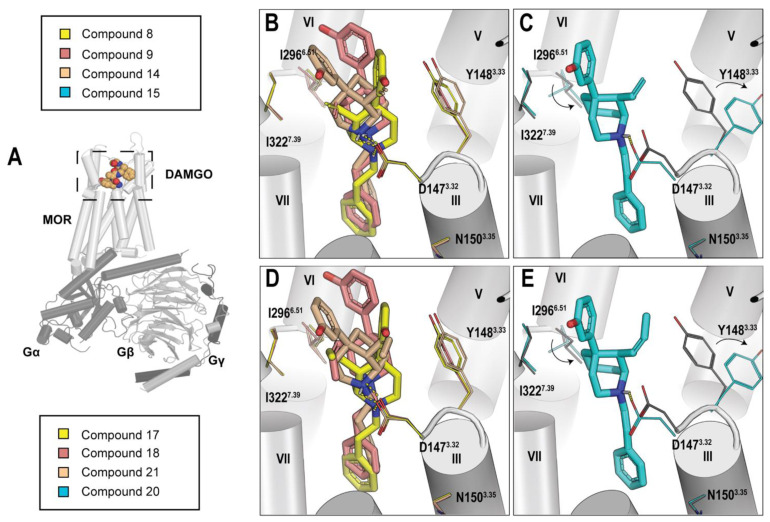
Representative docking poses of C9-substituted compounds in the active MOR. The C9-vinyl compounds **8**, **9**, **14**, and **15** are shown in (**B**,**C**) and the C9-propenyl compounds **17**, **18**, **21**, and **20** are shown in (**D**,**E**). A section of TM2 and TM3 were hidden to provide a better view of the binding site. (**A**) An overview of the DAMGO-bound MOR-Gi complex (6DDF). (**B**) The vinyl group of **8** (yellow) can form hydrophobic interactions with I322^7.39^. Due to the orientation of its vinyl group, **14** (tan) is unable to form this stabilizing interaction. (**C**) **15** (cyan) clashes with Y148^3.33^ and I296^6.51^ in the active cryo-EM receptor structure (gray), forcing these residues to adopt new configurations. Arrows show the movement of these residues from the initial active cryo-EM structure to the induced docking configuration. (**D**) The elongated substituent of **17** may form a stronger hydrophobic interaction with I322^7.39^. (**E**) The addition of a carbon to the vinyl group of the antagonist **20** (cyan) may make the clash with Y148^3.33^ more extreme in the active cryo-EM receptor structure (gray).

**Figure 4:**
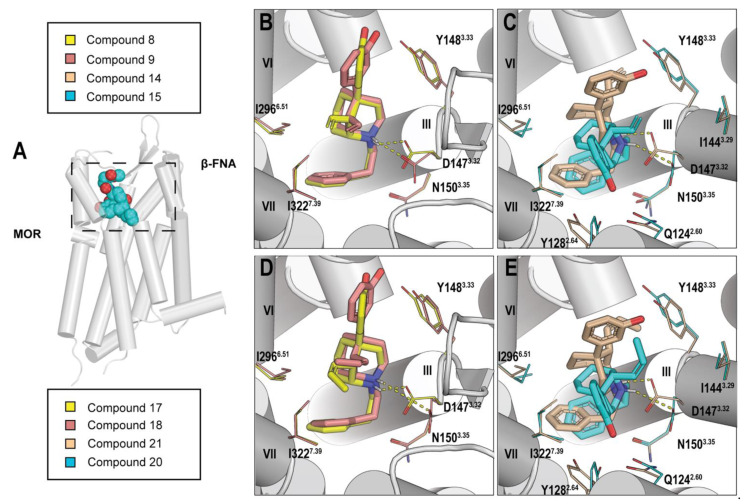
Representative docking poses of C9-substituted compounds in the inactive MOR. The binding poses of ligands in the inactive receptor adopt a more bent configuration, which is caused by the projection of N150^3.32^ into the binding site. The C9-vinyl compounds are shown in (**B**,**C**) and the C9-propenyl compounds are shown in (**D**,**E**). A section of TM2 and TM3 were hidden to generate a better view of the binding site. (**A**) An overview of the β-FNA-bound MOR (4DKL). (**B**) The vinyl group of **8** (yellow) may form weak interactions with I296^6.51^ and **9** (pink) may be stabilized by I322^7.39^. (**C**) **15** (cyan) does not clash with Y148^3.33^ and I296^6.51^ in the inactive receptor, and the central hexane group is stabilized by I322^7.39^. The phenol moiety forms hydrogen bonds with Q124^2.60^ and Y128^2.64^ (**D**) The elongated substituents of **18** (pink) and **17** (yellow) may be stabilized by I296^6.51^ and I322^7.39^, respectively. (**E**) The C9-propenyl antagonist **20** maintains stabilizing interactions with Q124^2.60^, Y128^2.64^, and I322^7.39^.

Specifically, the C9-vinyl agonists, **8**, **9**, and **14** can be easily accommodated in the binding pocket of the MOR in both active and inactive conformational states, as their docking poses do not reveal any clashes with binding site residues (Figure 3 and Figure 4).

In the active MOR model, these ligands adopt a more linear configuration where the *N*-phenethyl tail extends deeper into the binding pocket and is enclosed by A117^2.53^, M151^3.36^, W293^6.48^, and Y326^7.43^, whereas the phenol moiety points towards Q124^2.60^ (**14**), W318^7.35^ (**9**), or Y148^3.33^ (**8**) (Figure 3B). In the inactive model, the ligands are bent and shifted slightly, with the *N*-phenethyl tail in tight interactions only with W293^6.48^ and Y326^7.43^, and the phenol moiety oriented more towards TM5 compared to their poses in the active state (Figure 4B).

We then estimated the binding free energies of these docked ligands in the models by carrying out MM/GBSA calculations. In the active model, **8** has a lower binding free energy (−71.1 kcal/mol) than both **9** (−69.7 kcal/mol) and **14** (−65.3 kcal/mol) and is more favorably bound to the active receptor (Table 4). Between the active and inactive states of the MOR, the ΔΔG_active-inactive_ of **8**, **9**, and **14** are −10.3, −7.2, and −3.6 kcal/mol, respectively, suggesting that these agonists may favor the active state (Table 4).

##### C9-Vinyl Antagonist (15) Preference for the Inactive Conformation of the MOR

(1*S*,5*R*,9*R*)-**15**, which differs from (1*S*,*5R*,9*S*)-**14** only in the chirality of its C9-vinyl group, has been experimentally characterized as a MOR antagonist. In the same orientation as the agonists, the docked pose of **15** in the active model shows that its phenol moiety points towards Q124^2.60^. However, its vinyl and central hexane ring groups are not compatible with the original sidechain orientations of Y148^3.33^ and I296^6.51^, respectively, forcing these binding site residues to rotate away (Figure 3C). Additionally, the relatively high binding free energy of **15** (−47.3 kcal/mol) indicates that the antagonist does not bind favorably to the active state (Table 4). In contrast, this antagonist can be feasibly accommodated in the inactive MOR conformation, showing no clashes with binding site residues. Specifically, its vinyl group occupies a relatively open space near I144^3.29^, its phenyl ring has favorable aromatic interactions with W293^6.48^, while its phenol moiety engages in hydrogen bonds with both Q124^2.60^ and Y128^2.64^. In addition, the hydrophobic interactions between the central hexane ring and I322^7.39^ also stabilize the ligand in the inactive model (Figure 3C). MM/GBSA analysis reveals that **15** strongly favors the inactive state with a ΔΔG_active-inactive_ of 23.2 kcal/mol. Thus, **15** is not compatible with the active state binding site but can be well-accommodated by the inactive state of the MOR.

##### Corresponding Propenyl Analogs Observe a Similar Trend as Their C9-Vinyl Analogs

We then investigated a series of diastereomeric compounds, (1*S*,5*R*,9*R*)-**20**, (1*S*,5*R*,9*S*)-**21**, (1*R*,5*S*,9*S*)-**18**, and (1*R*,5*S*,9*R*)-**17**, which has an additional methyl added onto the vinyl group of compounds **15**, **14**, **9**, and **8**, respectively. Each compound of this series was treated as the Z-isomer in accordance with the X-ray spectroscopic analysis of **20** (Figure 4). We hypothesized that the extra methyl group worsens the clash of a MOR antagonist with Y148^3.33^. Indeed, the docked pose of **20** shows that the elongated substituent is more proximal to Y148^3.33^ (Figure 3E). MM/GBSA results show that **20** has a high binding free energy of −47.6 kcal/mol (Table 4). The (9*S*)-isoform of **20**, compound **21**, which is a MOR agonist, has a similar orientation to **20**; however, its propenyl tail is oriented away from Y148^3.33^ where adequate space can accommodate the extra methyl (Figure 3D). MM/GBSA results agree with these binding site differences, which show that **21** binds more favorably in the active model than **20** (Table 4).

Compound **18**, a propenyl analog of **9** in the vinyl series, acts as a potent agonist. Interestingly, the additional methyl of **18** overlaps with the space occupied by the vinyl group of **9**, the most favorably bound agonist of the vinyl series. As such, it may be important for the alkyl substituents of these agonists to protrude into this hydrophobic space, where they can interact with I322^7.39^ (Figure 3D). MM/GBSA results indicate that **18** is favored in the active model, with the lowest binding free energy of the series (−72.3 kcal/mol). Similarly, the propenyl agonist **17**, which had its C9-substituent modified from the vinyl agonist **8**, has its elongated propenyl substituent overlap with that of **18** and forms a strong hydrophobic interaction with I322^7.39^ as well.

Similar to the vinyl series, all of the propenyl analogs can be accommodated by the inactive model. However, the phenol moiety of the MOR antagonist **20** is able to form hydrogen bonds with Q124^2.60^ and Y128^2.64^. Additionally, the central hexane group remains located between TM6 and TM7 and engaged in hydrophobic interactions with I322^7.39^ (Figure 4E). MM/GBSA calculations show that **20** has a strong preference for the inactive state with a ΔΔG_active-inactive_ of 22.9 kcal/mol. In addition, our MM/GBSA results also indicate that **20** binds more favorably than the agonists in the inactive states.

### 2.3. In Vivo Data

#### Antinociceptive and Respiration Assays in Monkeys for Compounds **8**, **9** and **17**

**Figure 5:**
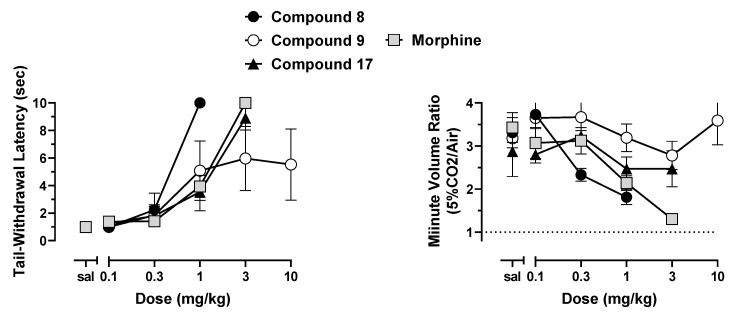
Effects of compounds **8**, **9**, and **17** (in comparison to morphine and saline) on tail withdrawal latency (**left panel**) and on ventilatory ratio (**right panel**) in squirrel monkeys. Compound **8** like morphine, significantly increased tail withdrawal latency and reduced the ability of 5% CO_2_ to stimulate increases in ventilation. Compound **17** increased tail withdrawal latency. without significantly altering ventilation, and Compound **9** had inconsistent effect of tail withdrawal latency and did not alter ventilation. Data are expressed as mean ± SEM (*n* = 3–5; results of statistical analysis are presented in text).

Compound **8**, a MOR agonist with high binding affinity for MOR (Ki = 0.5 nM) and with high efficacy in the cAMP assay (% E_max_ = 94.7), but not in the [^35^S]GTPgS assay (%E_max_ = 20.09), had antinociceptive effects (Figure 5) in nonhuman primates [F_(3,13)_ = 56.8, *p* < 0.01] and produced, with lesser maximal depression, morphine-like respiratory depressant effects [F_(3,12)_ = 17.5, *p* < 0.01], i.e., decreased the ratio of minute volumes in an atmosphere of 5% CO2 and room air; ventilatory ratio). Unlike **8**, compounds **9** and **17** were both partial agonists in the cAMP (% Emax = 67.3 and 89.5, respectively) and in the [^35^S]GTPgS assay (% E_max_ = 10.54 and 17.97, respectively). Their effects in vivo differed (Figure 5); compound **17** had consistent and significant antinociceptive effects [F_(4,15)_ = 17.2, *p* < 0.01] and limited effects on ventilatory ratio [F_(4,10)_ = 0.8, n.s.] whereas Compound **9** produced antinociceptive effects in some, but not all subjects [F_(5,19)_ = 2.1, n.s.], consistent with its quite low cAMP and [^35^S]GTPgS efficacy, and did not produce morphine-like decreases in ventilatory ratio [F_(4,14)_ = 0.8, n.s.]. Thus, both compounds **9** and **17** had less effect on respiratory depression than those observed with morphine, which is consistent with their designation as partial agonists with sufficient intrinsic efficacy for G-protein activation. Their intrinsic efficacy in our theoretically desirable range might be one of the causes [18] for their reduced effect on respiration.

## 3. Materials and Methods

### 3.1. General Information

Melting points were determined on a Mettler Toledo MP70 and are uncorrected. Proton and carbon nuclear magnetic resonance (^1^H and ^13^C NMR) spectra were recorded on a Varian Gemini-400 spectrometer in CDCl_3_ (unless otherwise noted) with the values given in ppm (TMS as internal standard) and J (Hz) assignments of ^1^H resonance coupling. The analyses were performed on the free base, unless otherwise noted. Mass spectra (HRMS) were recorded on a VG 7070E spectrometer or a JEOL SX102a mass spectrometer. The optical rotation data were obtained on a PerkinElmer polarimeter model 341. Thin layer chromatography (TLC) analyses were carried out on Analtech silica gel GHLF 0.25 mm plates using various gradients of CHCl_3_/MeOH containing 1% NH_4_OH or gradients of EtOAc/*n*-hexane. Visualization was accomplished under UV light or by staining in an iodine chamber. Flash column chromatography was performed with Fluka silica gel 60 (mesh 220 − 400). Flash column chromatography was performed using RediSep Rf normal phase silica gel cartridges. Robertson Microlit Laboratories, Ledgewood, NJ, USA, performed elemental analyses, and the results were within ±0.4% of the theoretical values.

### 3.2. Syntheses

*(1S,5S)-5-(3-Methoxyphenyl)-2-azabicyclo [3.3.1]nonan-9-one* (**2**): In an oven-dried flask, **1** (2.2 g, 8.3 mmol) and K_2_CO_3_ (2.3 g, 16.6 mmol) in 15 mL acetonitrile were treated with cyanogen bromide (3.3 mL, 16.6 mmol). The reaction mixture was stirred under a N_2_ atmosphere at room temperature for 2 h then heated to reflux. After 2 h, the reaction mixture was extracted with CHCl_3,_ and the organic phase was washed with brine and concentrated. The residue was taken up in a mixture of 21 mL 3 N HCl and 2.2 mL methanol and stirred at reflux for 17 h. Upon completion, the reaction mixture was cooled and quenched with 7 N NH_4_OH in methanol. The mixture was extracted with CHCl_3_ and washed with water, brine, dried with Na_2_SO_4_ and concentrated. Purification by flash column chromatography on silica gel (0–20% CMA in CHCl_3_) gave the red oil **2** (1.62 g, 80% yield). The ^1^H NMR of the product was identical to that of the known compound **2** [16].

*(1S,5S)-5-(3-Methoxyphenyl)-2-phenethyl-2-azabicyclo [3.3.1] nonan-9-one* (**3**): In an oven-dried flask, **2** (1.62 g, 6.6 mmol) and K_2_CO_3_ (1.82 g, 13.2 mmol) in 16 mL acetonitrile were treated with phenethyl bromide (1.34 mL, 9.9 mmol). The reaction mixture was stirred at reflux under a N_2_ atmosphere. After 16 h, the mixture was cooled, concentrated, and extracted with CHCl_3_. Purification by flash column chromatography on silica gel (0–75% EtOAc in Hexanes) gave a brown oil (1.78 g, 77% yield). The ^1^H NMR of the product **3** was identical to that of the known compound [16].

*(1S,5S)-9-(Methoxymethylene)-5-(3-methoxyphenyl)-2-phenethyl-2-azabicyclo [3.3.1] nonane* (**4**): An oven-dried flask charged with **3** (1.87 g, 5.1 mmol) and methoxy methylphosphonium chloride (5.25 g, 15.3 mmol) was evacuated and backfilled with N_2_ three times. 11 mL of THF was added and the reaction mixture was cooled to 0 °C. LHMDS (13.3 mL, 13.3 mmol) was added dropwise over 15 min. The reaction mixture was stirred at 0 °C for 30 min then allowed to warm to room temperature and stirred for an additional 22 h. Upon completion, the reaction mixture was cooled to 0 °C, quenched with methanol, concentrated, and extracted with CHCl_3,_ washed with water and brine, and dried with Na_2_SO_4_. Purification by flash column chromatography on silica gel (0–55% EtOAc in Hexanes) gave an orange oil (1.26 g, 65% yield, *E*:*Z* 1:4). The ^1^H NMR spectra of the product were identical to the known compound **4** [16].

*(1R,5S,9R)-5-(3-Methoxyphenyl)-2-phenethyl-9-vinyl-2-azabicyclo [3.3.1] nonane* (**6**): In an oven-dried flask, methoxy methylene **4** (650 mg, 1.56 mmol) was suspended in THF (7 mL) and treated with HCl (6 M, 12 mL) and the reaction mixture was stirred overnight at room temperature under argon. The reaction mixture was quenched with 7 N NH_4_OH in MeOH, extracted with CHCl_3_ and washed with water and brine. The organic layer was then dried with sodium sulfate, concentrated, and put under high vacuum for 2 h. An oven-dried round-bottom flask flushed with argon was charged with methyltriphenylphosphonium bromide (1.67 g, 3 equiv, 4.69 mmol) and potassium 2-methylpropan-2-olate (526 mg, 3 equiv, 4.69 mmol) and suspended in THF (15 mL). The reaction mixture was heated to 45 °C for 1 h and turned yellow when the ylide was formed. The dried aldehyde **5** was suspended in THF (10 mL) and was transferred to the ylide mixture. The reaction mixture was stirred at 45 °C for 2 h when it was complete by TLC, whereupon the reaction mixture was quenched with NH_4_OH in MeOH and extracted with EtOAc. The mixture was washed with water and brine, dried with sodium sulfate and concentrated. Purification by flash column chromatography on silica gel (0–100% EtOAc in hexanes) yielded a green oil (233 mg, 37% yield). Crystallization as the oxalate salt from acetone afforded **6** as a white solid, mp 170–175 ˚C. ^1^H-NMR (400 MHz; CD_3_OD): δ 7.33 (s, 4H), 7.33–7.20 (m, 3H), 6.97–6.91 (m, 2H), 6.76 (dd, *J* = 8.1, 1.2 Hz, 1H), 5.76–5.67 (m, 1H), 5.14 (m, 2H), 3.80–3.74 (m, 4H), 3.68–3.61 (m, 2H), 3.56–3.44 (m, 2H), 3.38 (dd, *J* = 5.5, 0.6 Hz, 1H), 3.14–3.11 (m, 2H), 2.41–1.93 (m, 8H). ^13^C NMR (101 MHz; CD_3_OD): δ 164.9, 159.8, 148.2, 136.5, 134.4, 129.0, 128.5, 126.8, 118.6, 117.8, 112.0, 110.9, 60.7, 55.7, 54.2, 49.4, 46.5, 37.8, 36.7, 30.4, 28.0, 19.6, 16.8; HRMS-ESI (*m*/*z*): [M + H^+^] calcd. for C_25_H_32_NO 362.2484; found 362; [a]^20^_D_ 18.2° (c 0.74, CHCl_3_).

*(1R,5S,9S)-5-(3-Methoxyphenyl)-2-phenethyl-9-vinyl-2-azabicyclo [3.3.1] nonane* (**7**): Methoxy methylene **4** was subjected to the same reaction conditions as with **6** to give **7**, which was isolated as a yellow oil (160 mg, 26% yield). Crystallization as the oxalate salt from acetone afforded **7** as a white solid, mp 180–184 ˚C. ^1^H NMR (400 MHz; CD_3_OD): δ 7.35–7.20 (m, 6H), 6.89–6.84 (m, 2H), 6.76 (dd, *J* = 8.2, 2.2 Hz, 1H), 5.73–5.64 (m, 1H), 5.35–5.24 (m, 2H), 3.94 (d, *J* = 8.6 Hz, 1H), 3.78–3.74 (m, 3H), 3.74–3.67 (m, 1H), 3.59–3.50 (m, 2H), 3.41–3.31 (m, 2H), 3.17–3.09 (m, 1H), 2.96–2.88 (m, 1H), 2.52–2.37 (m, 3H), 2.18–1.81 (m, 5H); ^13^C NMR (101 MHz; CD_3_OD): δ 166.2, 161.3, 149.7, 137.4, 136.3, 130.5, 130.05, 129.88, 128.4, 120.9, 119.0, 113.4, 112.2, 60.8, 56.6, 55.7, 51.7, 47.4, 41.8, 38.1, 31.5, 29.3, 24.1, 22.0; HRMS-ESI (*m*/*z*): [M + H^+^] calcd. for C_25_H_32_NO 362.2484; found 362.2485; [a]^20^_D_ –49.1° (c 0.2, CHCl_3_)

*3-((1R,5S,9R)-2-Phenethyl-9-vinyl-2-azabicyclo [3.3.1]nonan-5-yl)phenol* (**8**): In an oven-dried round-bottom flask, **6** (280 mg, 1 equiv, 360 µmol) was suspended in dichloromethane (6 mL) and the mixture was cooled to –78 °C. tribromoborane (147 µL, 3 equiv, 1.44 mmol) was added dropwise and the reaction was stirred at –78 °C. The reaction mixture was allowed to warm to room temperature and stirred overnight (16 h). Upon completion, the reaction mixture was cooled to 0 °C and quenched with MeOH and stirred for 30 min. 1N HCl (2 mL) was added, and the reaction mixture was distilled at 100 °C for 1 h. The reaction mixture was then cooled to 0 °C and made basic (>10.5) with NH_4_OH and extracted with 9:1 CHCl3: MeOH. The combined organic layers were washed with water and brine, dried with sodium sulfate and concentrated. Purification by flash column chromatography on silica gel (20–100% EtOAc in hexanes) gave a yellow oil (248 mg, 92% yield). The HCl salt of **8** was formed in *i*PrOH (1 mL) with 37% HCl (0.1 mL) and recrystallized from hot ethanol (3 mL) to give a white solid, mp 233–237 ˚C. ^1^H NMR (400 MHz; CD_3_OD): δ 7.38–7.33 (m, 4H), 7.28 (dq, *J* = 8.8, 4.3 Hz, 1H), 7.14 (t, *J* = 8.0 Hz, 1H), 6.88–6.83 (m, 2H), 6.63 (dd, *J* = 8.0, 1.9 Hz, 1H), 5.73 (tt, *J* = 10.4, 7.0 Hz, 1H), 5.22–5.14 (m, 2H), 3.78 (d, *J* = 0.2 Hz, 1H), 3.72–3.60 (m, 2H), 3.51 (dd, *J* = 10.0, 6.8 Hz, 2H), 3.39 (dd, *J* = 5.5, 0.4 Hz, 1H), 3.16 (dd, *J* = 10.4, 6.6 Hz, 2H), 2.38–1.93 (m, 8H); ^13^C NMR (101 MHz; CD_3_OD): δ 158.6, 149.4, 137.7, 135.8, 130.4, 129.99, 129.89, 128.3, 120.0, 118.0, 114.3, 114.1, 62.0, 57.0, 51.1, 48.0, 39.4, 38.0, 31.8, 29.4, 21.1, 18.1; HRMS-ESI (*m*/*z*): [M + H^+^] calcd. for C_24_H_30_NO 348.2327; found 348.2328; Anal. Calcd. for C_24_H_30_ClNO: C, 75.08%; H, 7.88%; N, 3.65%. Found C, 75.12%; H 7.84%; N, 3.64%; [a]^20^_D_ 22.5° (c 0.64, CHCl_3_).

*3-((1R,5S,9S)-2-Phenethyl-9-vinyl-2-azabicyclo [3.3.1]nonan-5-yl)phenol* (**9**): In an oven-dried round-bottom flask, **7** (280 mg, 1 equiv, 360 µmol) was suspended in dichloromethane (6 mL) and the mixture was cooled to –78 °C. tribromoborane (147 µL, 3 equiv, 1.44 mmol) was added dropwise and the reaction was stirred at –78 °C. The reaction mixture was allowed to warm to room temperature and stirred overnight (16 h). Upon completion, the reaction mixture was cooled to 0 °C and quenched with MeOH and stirred for 30 min. 1 N HCl (2 mL) was added, and the reaction mixture was distilled at 100 °C for 1 h. The reaction mixture was then cooled to 0 °C and made basic (pH > 10.5) with NH_4_OH and extracted with 9:1 CHCl_3_: MeOH. The combined organic layers were washed with water and brine, dried with sodium sulfate and concentrated. Purification by flash column chromatography on silica gel (20–00% EtOAc in hexanes) gave **9** as a yellow oil (248 mg, 92% yield). The HCl salt of **9** was formed in *i*PrOH (0.5 mL) with 37% HCl (0.05 mL) and recrystallized from hot ethanol (3 mL) to give a white solid (173 mg, 64% yield), mp 264–267 ˚C. ^1^H NMR (400 MHz; CD_3_OD): δ 7.38–7.26 (m, 5H), 7.13 (t, *J* = 8.0 Hz, 1H), 6.79 (d, *J* = 7.9 Hz, 1H), 6.75 (s, 1H), 6.63 (dd, *J* = 8.0, 1.7 Hz, 1H), 5.74 (ddd, *J* = 17.4, 10.7, 6.7 Hz, 1H), 5.33 (dd, *J* = 24.4, 14.0 Hz, 2H), 3.97 (d, *J* = 0.5 Hz, 1H), 3.77–3.69 (m, 1H), 3.57 (td, *J* = 11.9, 5.9 Hz, 2H), 3.38 (td, *J* = 12.1, 5.2 Hz, 1H), 3.31 (t, *J* = 1.5 Hz, 2H), 3.16 (td, *J* = 12.2, 5.6 Hz, 1H), 2.98–2.90 (m, 1H), 2.51–2.36 (m, 3H), 2.20–2.03 (m, 2H), 2.01–1.83 (m, 3H); ^13^C NMR (101 MHz; CD_3_OD): δ 158.6, 149.7, 137.4, 136.4, 130.5, 130.04, 129.9, 128.4, 120.9, 117.8, 114.2, 113.8, 60.5, 56.4, 51.9, 47.3, 41.8, 38.0, 31.5, 29.3, 24.0, 22.0. HRMS-ESI (*m*/*z*): [M + H^+^] calcd. for C_24_H_30_NO 348.2327; found 348.2328; Anal. Calcd. for C_24_H_30_ClNO·0.5 H_2_O: C, 73.36%; H, 7.95%; N, 3.56%. Found C, 73.11%; H, 7.68%; N, 3.55%; [a]^20^_D_ –35.8° (c 0.45, CHCl_3_).

*(1S,5R,9S)-5-(3-Methoxyphenyl)-2-phenethyl-9-vinyl-2-azabicyclo [3.3.1]nonane* (**12**). In an oven-dried flask, methoxy methylene **10** (590 mg, 1.56 mmol) [12] was suspended in THF (6 mL) and treated with HCl (6 M, 10 mL) and the reaction mixture was stirred overnight at room temperature under argon. The reaction mixture was quenched with 7 N NH_4_OH in MeOH, extracted with CHCl_3_ and washed with water and brine. The organic layer was then dried with sodium sulfate, concentrated, and put under high vacuum for 2 h. An oven-dried round-bottom flask flushed with argon was charged with methyltriphenylphosphonium bromide (1.67 g, 3 equiv, 4.69 mmol) and potassium 2-methylpropan-2-olate (526 mg, 3 equiv, 4.69 mmol) and suspended in THF (13 mL). The reaction mixture was heated to 45 °C for 1 h and turned yellow when the ylide was formed. The dried aldehyde (**11**) was suspended in THF (10 mL) and was transferred to the ylide mixture. The reaction mixture was stirred at 45 °C for 2 h when it was complete by TLC, whereupon the reaction mixture was quenched with NH_4_OH in MeOH and extracted with EtOAc. The mixture was washed with water and brine, dried with sodium sulfate and concentrated. Purification by flash column chromatography on silica gel (0–100% EtOAc in hexanes) gave **12** as a green oil (183 mg, 32% yield). δ 7.32–7.17 (m, 6H), 6.99–6.94 (m, 2H), 6.72–6.69 (m, 1H), 5.76 (ddd, *J* = 17.4, 10.4, 7.8 Hz, 1H), 5.09–4.98 (m, 2H), 3.81–3.76 (m, 3H), 3.08 (td, *J* = 13.8, 4.3 Hz, 4H), 2.91–2.78 (m, 4H), 2.21–1.77 (m, 7H), 1.60 (ddt, *J* = 17.1, 9.6, 4.4 Hz, 1H). ^13^C NMR (100 MHz; CDCl_3_): δ 159.3, 151.4, 140.5, 138.3, 128.9, 128.7, 128.4, 126.0, 118.4, 116.9, 112.4, 110.4, 58.4, 57.9, 55.1, 49.8, 49.4, 41.22, 38.1, 34.6, 29.7, 22.1, 19.3.

*(1S,5R,9R)-5-(3-Methoxyphenyl)-2-phenethyl-9-vinyl-2-azabicyclo [3.3.1]nonane* (**13**): Methoxy methylene **10** (590 mg, 1.56 mmol) was suspended in THF (6 mL) and treated with HCl (6 M, 10 mL) and the reaction mixture was stirred overnight at room temperature under argon. The reaction mixture was quenched with 7 N NH_4_OH in MeOH, extracted with CHCl_3_ and washed with water and brine. The organic layer was then dried with sodium sulfate and concentrated and dried under reduced pressure for 2 h. An oven-dried round-bottom flask flushed with argon was charged with methyltriphenylphosphonium bromide (1.67 g, 3 equiv, 4.69 mmol) and potassium 2-methylpropan-2-olate (526 mg, 3 equiv, 4.69 mmol). THF (13 mL) was added, and the reaction mixture was heated to 45 °C for 1 h. The solution turned yellow when the ylide was formed and the aldehyde mixture **11** was added and stirred at 45 °C for 2 h. Upon completion by TLC, the reaction mixture was quenched with NH_4_OH in MeOH and extracted with EtOAc. The mixture was washed with water and brine, dried with sodium sulfate and concentrated. Purification by flash column chromatography on silica gel (0–100% EtOAc in hexanes) gave **13** as a blue oil (138 mg, 24% yield). ^1^H NMR (400 MHz; CDCl_3_): δ 7.36–7.32 (m, 2H), 7.27–7.17 (m, 4H), 6.94–6.89 (m, 2H), 6.71 (dd, *J* = 8.1, 2.0 Hz, 1H), 6.01 (dt, *J* = 17.4, 8.9 Hz, 1H), 4.94 (t, *J* = 15.2 Hz, 2H), 3.84–3.79 (m, 3H), 3.16 (dq, *J* = 18.1, 5.9 Hz, 3H), 2.86–2.71 (m, 5H), 2.46–2.31 (m, 2H), 2.10–1.67 (m, 5H), 1.60–1.49 (m, 1H).

*3-((1S,5R,9S)-2-Phenethyl-9-vinyl-2-azabicyclo [3.3.1]nonan-5-yl)phenol* (**14**): In an oven-dried round-bottom flask, **12** (750 mg, 1 equiv, 360 µmol) was suspended in dichloromethane (3 mL) and the mixture was cooled to –78 °C. tribromoborane (136 µL, 3 equiv, 1.44 mmol) was added dropwise and the reaction was stirred at –78 °C. The reaction mixture was allowed to warm to room temperature and stirred overnight (16 h). Upon completion, the reaction mixture was cooled to 0 °C and quenched with MeOH and stirred for 30 min. 1 N HCl (2 mL) was added, and the reaction mixture was distilled at 100 °C for 1 h. The reaction mixture was then cooled to 0 °C and made basic (>10.5) with NH_4_OH and extracted with 9:1 CHCl_3_: MeOH. The combined organic layers were washed with water and brine, dried with sodium sulfate and concentrated. Purification by flash column chromatography on silica gel (20–100% EtOAc in hexanes) gave **14** as a yellow oil (558 mg, 77% yield). The HCl salt of **14** was formed in *i*PrOH (2 mL) with 37% HCl (0.2 mL) and recrystallized from hot ethanol (4 mL) and cooled, stirring 16 h to give a white solid, mp 231–235 °C. ^1^H-NMR (400 MHz; CD_3_OD): δ 7.36 (d, *J* = 13.0 Hz, 4H), 7.28 (dq, *J* = 8.5, 4.3 Hz, 1H), 7.13 (t, *J* = 8.0 Hz, 1H), 6.88–6.84 (m, 2H), 6.63 (dd, *J* = 8.0, 2.0 Hz, 1H), 5.77–5.68 (m, 1H), 5.22–5.13 (m, 2H), 3.78 (s, 1H), 3.71–3.58 (m, 2H), 3.51 (quintet, *J* = 8.9 Hz, 2H), 3.41 (d, *J* = 5.6 Hz, 1H), 3.17 (dd, *J* = 10.4, 6.5 Hz, 2H), 2.39–1.92 (m, 8H). ^13^C NMR (101 MHz; CD_3_OD): δ 158.6, 149.5, 137.8, 135.8, 130.4, 129.98, 129.91, 128.3, 120.0, 118.0, 114.3, 114.1, 62.0, 57.0, 51.0, 47.9, 39.3, 38.0, 31.8, 29.4, 21.1, 18.1. HRMS-ESI (*m*/*z*): [M + H^+^] calcd for C_25_H_32_NO 348.2327; found 348.2328; Anal. calcd. for C_24_H_30_ClNO: C, 75.08%; H, 7.88%; N, 3.65%. Found C, 75.00%; H, 7.86%; N, 3.64%; [a]^20^_D_ −24.3° (c 0.64, CHCl_3_).

*3-((1S,5R,9R)-2-Phenethyl-9-vinyl-2-azabicyclo [3.3.1]nonan-5-yl)pheno*l (**15**): In an oven-dried round-bottom flask, **13** (750 mg, 1 equiv, 360 µmol) was suspended in dichloromethane (3 mL) and the mixture was cooled to –78 °C. tribromoborane (136 µL, 3 equiv, 1.44 mmol) was added drop-wise and the reaction was stirred at –78 °C. The reaction mixture was allowed to warm to room temperature and stirred overnight (16 h). Upon completion, the reaction mixture was cooled to 0 °C and quenched with MeOH and stirred for 30 min. 1 N HCl (2 mL) was added, and the reaction mixture was distilled at 100 °C for 1 h. The reaction mixture was then cooled to 0 °C and made basic (>10.5) with NH_4_OH and extracted with 9:1 CHCl_3_: MeOH. The combined organic layers were washed with water and brine, dried with sodium sulfate and concentrated. Purification by flash column chromatography on silica gel (20–100% EtOAc in hexanes) gave **15** as a yellow oil (554 mg, 76% yield). The HCl salt of **15** was formed in *i*PrOH with 37% HCl (0.1 mL) and recrystallized from ethanol to give a white solid, mp 264–266 °C. ^1^H-NMR (400 MHz; CD_3_OD): δ 7.38–7.26 (m, 5H), 7.13 (t, *J* = 8.0 Hz, 1H), 6.79 (d, *J* = 7.9 Hz, 1H), 6.75 (t, *J* = 1.9 Hz, 1H), 6.63 (dd, *J* = 8.0, 2.3 Hz, 1H), 5.74 (ddd, *J* = 17.4, 10.7, 6.7 Hz, 1H), 5.33 (dd, *J* = 25.2, 14.0 Hz, 2H), 3.97 (t, *J* = 0.5 Hz, 1H), 3.73 (td, *J* = 13.3, 5.9 Hz, 1H), 3.61–3.54 (m, 2H), 3.38 (td, *J* = 12.1, 5.2 Hz, 1H), 3.16 (td, *J* = 12.2, 5.6 Hz, 1H), 2.94 (td, *J* = 12.1, 5.1 Hz, 1H), 2.51–2.36 (m, 3H), 2.20–1.83 (m, 5H); ^13^C NMR (101 MHz; CD_3_OD): δ 158.6, 149.7, 137.4, 136.4, 130.5, 130.04, 129.9, 128.4, 120.9, 117.8, 114.2, 113.8, 60.5, 56.4, 51.9, 47.3, 41.8, 38.0, 31.5, 29.3, 24.0, 22.0; HRMS-ESI (*m*/*z*): [M + H^+^] calcd. for C_24_H_30_NO 348.2327; found 348.2328; Anal. calcd. for C_24_H_30_ClNO: C, 75.08%; H, 7.88%; N, 3.65%. Found C, 74.78%; H, 7.48%; N, 3.61%; [a]^20^_D_ –35.6° (c 0.95, CHCl_3_).

*3-((1R,5S,9R)-2-Phenethyl-9-((Z)-prop-1-en-1-yl)-2-azabicyclo [3.3.1]nonan-5-yl)phenol* (**17**): In an oven-dried flask, **4** (1 g, 3 mmol) was suspended in THF (10 mL) and treated with HCl (6 M, 10 mL) and the reaction mixture was stirred overnight at room temperature under nitrogen. The reaction mixture was quenched with 7 N NH_4_OH in MeOH, extracted with CHCl_3_ and washed with water and brine. The organic layer was then dried with sodium sulfate, concentrated, to yield the aldehyde intermediate **5**. The aldehyde was treated with ethyltriphenylphosphonium iodide (3 g, 3 equiv, 8 mmol) and suspended in THF (10 mL). The reaction mixture was cooled to 0 °C and treated slowly with LiHMDS (1.0 M in THF) (7 mL, 2.6 equiv, 7 mmol). After 30 min, the reaction mixture was warmed to room temperature then the mixture was heated to 45 °C for 16 h. Upon completion by TLC, the reaction mixture was quenched with MeOH and extracted with CHCl_3_. The mixture was washed with water and brine, dried with sodium sulfate and concentrated. Purification by flash column chromatography on silica gel (0–50% EtOAc in hexanes) yielded a mixture of C9 epimers **16** which was used without further purification.

In an oven-dried round-bottom flask, **16** (500 mg, 1 equiv, 1.3 mmol) was suspended in dichloromethane (15 mL) and the mixture was cooled to –78 °C. Tribromoborane (667 mg, 253 µL, 2 equiv, 2.66 mmol) was added to drop-wise and the reaction was stirred at –78 °C for 15 min. The reaction mixture was allowed to warm to room temperature and stirred 2 h. Upon completion, the reaction mixture was cooled to 0 °C and quenched with 7 mL MeOH drop wise and stirred for 30 min. subsequently, 10 mL 1 N HCl was added, and the reaction mixture was distilled at 100 °C for 1 h. The reaction mixture was then cooled to 0 °C and made basic (>10.5) with NH_4_OH and extracted with 9:1 CHCl_3_: MeOH. The combined organic layers were washed with water and brine, dried with sodium sulfate and concentrated. Purification by silica gel column chromatography 0–60% EtOAc: Hexanes. **17** was isolated as a white foam (153 mg, 32% yield) as the more polar fraction. The HCl salt of **17** was formed in *i*PrOH with 37% HCl (0.1 mL) and recrystallized from ethanol to give a white solid: mp 271–275 °C; ^1^H NMR (400 MHz; CD_3_OD): δ 7.38–7.33 (m, 4H), 7.31–7.26 (m, 1H), 7.11 (t, *J* = 8.0 Hz, 1H), 6.84–6.79 (m, 2H), 6.61 (dd, *J* = 8.0, 2.2 Hz, 1H), 5.61–5.53 (m, 1H), 5.39 (ddd, *J* = 10.6, 8.8, 1.7 Hz, 1H), 3.68 (dt, *J* = 21.3, 7.7 Hz, 4H), 3.52–3.48 (m, 2H), 3.15 (dt, *J* = 10.3, 6.0 Hz, 2H), 2.34–2.19 (m, 3H), 2.19–1.91 (m, 5H), 1.76 (dd, *J* = 6.9, 1.6 Hz, 3H). ^13^C NMR (100 MHz; CD_3_OD): δ 158.5, 149.8, 137.7, 130.3, 130.00, 129.91, 129.5, 128.3, 127.4, 117.7, 114.2, 113.8, 61.1, 56.8, 51.1, 41.9, 39.9, 37.8, 31.8, 29.2, 21.0, 18.7, 13.8; HRMS-ESI (*m*/*z*): [M + H^+^] calcd. for C_25_H_32_NO 362.2484; found 362.2485; Anal. calcd. for C_25_H_32_ClNO·0.1 H_2_O: C, 75.11%; H, 8.12%; N, 3.5%. Found C, 75.00%; H, 7.87%; N, 3.42%; [a]^20^_D_ –14.1° (c 0.82, CHCl_3_).

*3-((1R,5S,9S)-2-Phenethyl-9-((Z)-prop-1-en-1-yl)-2-azabicyclo [3.3.1]nonan-5-yl)phenol* (**18**): From the same procedure as in **17**, **18** was isolated as a white foam (133 mg, 55% yield) as the less polar fraction. The HCl salt of **18** was formed in *i*PrOH with 37% HCl (0.1 mL) and recrystallized from ethanol to give a white solid: mp 260–264 °C. ^1^H NMR (400 MHz; CD_3_OD): δ 7.35–7.24 (m, 5H), 7.08 (t, *J* = 8.0 Hz, 1H), 6.73 (d, *J* = 8.0 Hz, 1H), 6.68 (d, *J* = 1.8 Hz, 1H), 6.59 (dd, *J* = 8.0, 1.8 Hz, 1H), 5.70–5.63 (m, 1H), 5.34–5.29 (m, 1H), 3.73–3.67 (m, 2H), 3.59–3.46 (m, 3H), 3.39–3.32 (m, 1H), 3.15–3.07 (m, 1H), 2.93–2.86 (m, 1H), 2.55–2.46 (m, 1H), 2.40–2.35 (m, 2H), 2.17–2.03 (m, 2H), 1.99–1.82 (m, 3H), 1.76 (dd, *J* = 7.0, 1.3 Hz, 3H). ^13^C NMR (100 MHz; CD_3_OD): δ 158.5, 150.0, 137.4, 131.0, 130.3, 130.03, 129.88, 128.4, 127.7, 117.5, 114.1, 113.6, 60.4, 56.5, 51.8, 42.35, 42.19, 37.8, 31.5, 29.1, 23.9, 22.1, 13.7; HRMS-ESI (*m*/*z*): [M + H^+^] calcd. for C_25_H_32_NO 362.2484; found 362.2486; C_25_H_32_ClNO·1.45 H_2_O calc. C: 70.8; H: 8.29; N: 3.3; found C: 70.5; H: 7.92; N: 3.3; [a]^20^_D_ –22.4° (c 1.0, CHCl_3_).

*3-((1S,5R,9R)-2-Phenethyl-9-((Z)-prop-1-en-1-yl)-2-azabicyclo [3.3.1]nonan-5-yl)phenol* (**20**): In an oven-dried flask, **10** (1 g, 3 mmol) was suspended in THF (10 mL) and treated with HCl (6 M, 10 mL) and the reaction mixture was stirred overnight at room temperature under nitrogen. The reaction mixture was quenched with 7 N NH_4_OH in MeOH, extracted with CHCl_3_ and washed with water and brine. The organic layer was then dried with sodium sulfate, concentrated, to yield the aldehyde intermediate. The aldehyde was treated with ethyltriphenylphosphonium iodide (3 g, 3 equiv, 8 mmol) and suspended in THF (10 mL). The reaction mixture was cooled to 0 °C and treated slowly with LiHMDS (1.0 M in THF) (7 mL, 2.6 equiv, 7 mmol). After 30 min, the reaction mixture was warmed to room temperature then the mixture was heated to 45 °C for 16 h. Upon completion by TLC, the reaction mixture was quenched with MeOH and extracted with CHCl_3_. The mixture was washed with water and brine, dried with sodium sulfate and concentrated. Purification by flash column chromatography on silica gel (0–50% EtOAc in hexanes) yielded a mixture of C9 epimers **19** which was used without further purification.

In an oven-dried round-bottom flask, **19** (700 mg, 1 equiv, 2 mmol) was suspended in dichloromethane (5 mL) and the mixture was cooled to –78 °C. Tribromoborane (900 mg, 400 µL, 2 equiv, 4 mmol) was added to drop-wise and the reaction was stirred at –78 °C for 15 min. The reaction mixture was allowed to warm to room temperature and stirred 2 h. Upon completion, the reaction mixture was cooled to 0 °C and quenched with 7 mL MeOH drop wise and stirred for 30 min. subsequently, 10 mL 1 N HCl was added, and the reaction mixture was distilled at 100 °C for 1 h. The reaction mixture was then cooled to 0 °C and made basic (>10.5) with NH_4_OH and extracted with 9:1 CHCl_3_: MeOH. The combined organic layers were washed with water and brine, dried with sodium sulfate and concentrated. Purification by silica gel column chromatography 0–60% EtOAc: Hexanes. **20** was isolated as a white foam (175 mg, 30% yield) as the less polar fraction. The HCl salt of **20** was formed in *i*PrOH with 37% HCl (0.1 mL) and recrystallized from ethanol to give a white solid: mp 269–274; ^1^H NMR (400 MHz; CD_3_OD): δ 7.35–7.24 (m, 5H), 7.10–7.06 (m, 1H), 6.77–6.68 (m, 2H), 6.61–6.57 (m, 1H), 5.71–5.63 (m, 1H), 5.36–5.30 (m, 1H), 3.73–3.66 (m, 2H), 3.59–3.47 (m, 3H), 3.36 (td, *J* = 12.0, 5.3 Hz, 1H), 3.11 (td, *J* = 12.0, 5.5 Hz, 1H), 2.94–2.87 (m, 1H), 2.55–2.47 (m, 1H), 2.44–2.35 (m, 2H), 2.18–2.02 (m, 2H), 1.99–1.80 (m, 3H), 1.81–1.74 (m, 3H). ^13^C NMR (101 MHz; CD_3_OD): δ 158.5, 150.0, 137.4, 131.0, 130.3, 130.03, 129.9, 128.4, 127.7, 117.5, 114.1, 113.6, 60.4, 56.5, 51.8, 42.4, 42.2, 37.8, 31.5, 29.1, 23.9, 22.1, 13.7. HRMS-ESI (*m*/*z*): [M + H^+^] calcd. for C_25_H_32_NO 362.2484; found 362.2487. Anal. calcd. for C_25_H_32_ClNO·0.15 H_2_O: C, 74.94%; H, 8.13%; N, 3.5%. Found C, 74.8%; H, 7.85%; N, 3.36%; [a]^20^_D_ 22.3° (c 1.0, CHCl_3_).

*3-((1S,5R,9S)-2-Phenethyl-9-((Z)-prop-1-en-1-yl)-2-azabicyclo [3.3.1]nonan-5-yl)phenol* (**21**): From the same reaction as **20**. Alkene **21** isolated as a light green foam (157 mg, 20% yield) as the more polar fraction. The HCl salt of **21** was formed in *i*PrOH with 37% HCl (0.1 mL) and recrystallized from ethanol to give a white solid: mp 277–281; ^1^H-NMR (400 MHz; CD_3_OD): δ 7.38–7.33 (m, 4H), 7.29 (tt, *J* = 9.1, 4.7 Hz, 1H), 7.12–7.09 (m, 1H), 6.84–6.80 (m, 1H), 6.61 (dd, *J* = 8.0, 2.1 Hz, 1H), 5.61–5.53 (m, 1H), 5.41–5.36 (m, 1H), 3.72–3.63 (m, 4H), 3.50 (dd, *J* = 10.4, 6.5 Hz, 2H), 3.17–3.13 (m, 2H), 2.34–2.21 (m, 3H), 2.19–1.91 (m, 5H), 1.76 (dd, *J* = 6.9, 1.1 Hz, 3H); ^13^C NMR (101 MHz; CD_3_OD): δ 158.5, 149.8, 137.7, 130.3, 130.00, 129.91, 129.5, 128.3, 127.4, 117.8, 114.2, 113.8, 61.1, 56.8, 51.1, 41.9, 39.8, 37.8, 31.8, 29.2, 21.1, 18.7, 13.8; HRMS-ESI (*m*/*z*): [M + H^+^] calcd. for C_25_H_32_NO 362.2484; found 362.2484; Anal. calcd. for C_25_H_32_ClNO: C, 75.45%; H, 8.7%; N, 3.52%. Found C, 75.27%; H, 7.73%; N, 3.44%; [a]^20^_D_ 15.0° (c 0.86, CHCl_3_).

*3-((1R,5S,9R)-9-((Z)-But-1-en-1-yl)-2-phenethyl-2-azabicyclo [3.3.1]nonan-5-yl)phenol* (**23**): In an oven-dried flask, **4** (1.2 g, 3.2 mmol) was suspended in THF (12 mL) and treated with HCl (6 M, 12 mL) and the reaction mixture was stirred overnight at room temperature under nitrogen. The reaction mixture was quenched with 7 N NH_4_OH in MeOH, extracted with CHCl_3_ and washed with water and brine. The organic layer was then dried with sodium sulfate, concentrated, to yield an aldehyde intermediate. The aldehyde intermediate was treated with propyltriphenylphosphonium bromide (3.7 g, 3 equiv, 9.5 mmol) and suspended in THF (12 mL). The reaction mixture was cooled to 0 °C and treated slowly with LiHMDS (1.0 M in THF) (8.3 mL, 2.6 equiv, 8.3 mmol). After 30 min, the reaction mixture was warmed to room temperature then the mixture was heated to 45 °C for 16 h. Upon completion by TLC, the reaction mixture was quenched with MeOH and extracted with CHCl_3_. The mixture was washed with water and brine, dried with sodium sulfate and concentrated. Purification by flash column chromatography on silica gel (0–50% EtOAc in hexanes) yielded a mixture of C9 epimers **22** which were used in the next reaction without further purification or characterization.

In an oven-dried round-bottom flask, **22** (800 mg, 1 equiv, 2.05 mmol) was suspended in dichloromethane (20 mL) and the mixture was cooled to –78 °C. Tribromoborane (1.03 g, 390 µL, 2 equiv, 4.11 mmol) was added to drop-wise and the reaction was stirred at –78 °C for 15 min. The reaction mixture was allowed to warm to room temperature and stirred 2 h. Upon completion, the reaction mixture was cooled to 0 °C and quenched with 10 mL MeOH drop wise and stirred for 30 min. subsequently, 15 mL 1 N HCl was added, and the reaction mixture was distilled at 100 °C for 1 h. The reaction mixture was then cooled to 0 °C and made basic (>10.5) with NH_4_OH and extracted with 9:1 CHCl_3_: MeOH. The combined organic layers were washed with water and brine, dried with sodium sulfate and concentrated. Purification by silica gel column chromatography 0–60% EtOAc: Hexanes. **23** was isolated as a white foam (305 mg, 40% yield) as the more polar fraction. The HCl salt of **23** was formed in *i*PrOH (1.5 mL) with 37% HCl (0.15 mL) and recrystallized from hot ethanol (5 mL) to give a white solid: mp 265–268 °C. ^1^H-NMR (400 MHz; CD_3_OD): δ 7.33 (d, *J* = 4.3 Hz, 4H), 7.29–7.23 (m, 1H), 7.08 (t, *J* = 7.9 Hz, 1H), 6.82–6.78 (m, 2H), 6.59 (dd, *J* = 7.9, 1.7 Hz, 1H), 5.45–5.38 (m, 1H), 5.32–5.27 (m, 1H), 3.65–3.56 (m, 4H), 3.49–3.45 (m, 2H), 3.12 (dd, *J* = 10.6, 5.9 Hz, 2H), 2.35–1.90 (m, 11H), 0.95 (t, *J* = 7.5 Hz, 3H). ^13^C NMR (100 MHz; CD_3_OD): δ 158.5, 149.8, 137.7, 137.1, 130.2, 129.99, 129.91, 128.3, 125.6, 117.8, 114.2, 113.9, 61.6, 56.8, 51.1, 42.3, 39.7, 37.8, 31.8, 29.1, 22.2, 21.1, 18.6, 14.3. HRMS-ESI (*m*/*z*): [M + H^+^] calcd. for C_26_H_34_NO 376.2640; found 376.2642; Anal. calcd. for C_26_H_34_ClNO·0.05 C_2_H_6_O: calc. C: 75.66%; H: 8.34%; N: 3.38%; found C: 75.65%; H: 8.31%; N: 3.34%; [a]^20^_D_° −4.1 (c 0.96, CHCl_3_).

*3-((1R,5S,9S)-9-((Z)-But-1-en-1-yl)-2-phenethyl-2-azabicyclo [3.3.1]nonan-5-yl)phenol* (**24**): From the same reaction as **23**, alkene **24** was isolated as a white foam (250 mg, 32% yield) as the less polar fraction. The HCl salt of **24** was formed in *i*PrOH (1.5 mL) with 37% HCl (0.15 mL) and recrystallized from hot ethanol (5 mL) to give a white solid: mp 262–265 °C. ^1^H NMR (400 MHz; CDCl_3_): δ 7.38–7.25 (m, 5H), 7.14–7.07 (m, 1H), 6.79–6.74 (m, 1H), 6.73–6.69 (m, 1H), 6.64–6.60 (m, 1H), 5.60–5.54 (m, 1H), 5.36–5.27 (m, 1H), 3.77–3.67 (m, 2H), 3.62–3.52 (m, 2H), 3.52–3.45 (m, 1H), 3.43–3.33 (m, 1H), 3.17–3.09 (m, 1H), 2.96–2.87 (m, 1H), 2.59–2.49 (m, 1H), 2.46–2.34 (m, 2H), 2.30–1.82 (m, 8H), 1.09–1.00 (m, 3H). ^13^C NMR (101 MHz; CD_3_OD): δ 158.5, 150.0, 138.5, 137.4, 130.3, 130.0, 129.9, 128.4, 125.9, 117.6, 114.1, 113.7, 60.9, 56.5, 51.8, 42.7, 42.1, 37.8, 31.5, 29.0, 23.9, 22.2, 22.1, 14.2. HRMS-ESI (*m*/*z*): [M + H^+^] calcd. for C_26_H_34_NO 376.2640; found 376.2641; Anal. calcd. for C_26_H_34_ClNO·0.1 H_2_O·0.1 C_2_H_6_O: C, 75.21%; H, 8.38%; N, 3.35%. Found C, 75.23%; H, 8.4%; N, 3.38%; [a]^20^_D_° −14.2 (c 0.8, CHCl_3_).

*3-((1S,5R,9S)-9-((Z)-But-1-en-1-yl)-2-phenethyl-2-azabicyclo [3.3.1]nonan-5-yl)phenol* (**26**): In an oven-dried flask, **10** (2 g, 5.3 mmol) was suspended in THF (20 mL) and treated with HCl (6 M, 20 mL) and the reaction mixture was stirred overnight at room temperature under nitrogen. The reaction mixture was quenched with 7 N NH_4_OH in MeOH, extracted with CHCl_3_ and washed with water and brine. The organic layer was then dried with sodium sulfate, concentrated, to yield the aldehyde intermediate. The aldehyde was treated with propyltriphenylphosphonium bromide (6.1 g, 3 equiv, 16 mmol) and suspended in THF (20 mL). The reaction mixture was cooled to 0 °C and treated slowly with LiHMDS (1.0 M in THF) (14 mL, 2.6 equiv, 14 mmol). After 30 min, the reaction mixture was warmed to room temperature then the mixture was heated to 45 °C for 16 h. Upon completion by TLC, the reaction mixture was quenched with MeOH and extracted with CHCl_3_. The mixture was washed with water and brine, dried with sodium sulfate and concentrated. Purification by flash column chromatography on silica gel (0–50% EtOAc in hexanes) yielded a mixture of C9 epimers **25** which was used without further purification.

In an oven-dried round-bottom flask, **25** (1.09 g, 1 equiv, 2.8 mmol) was suspended in dichloromethane (15 mL) and the mixture was cooled to –78 °C. Tribromoborane (1.4 g, 531 µL, 2 equiv, 5.6 mmol) was added to drop-wise and the reaction was stirred at –78 °C for 15 min. The reaction mixture was allowed to warm to room temperature and stirred 2 h. Upon completion, the reaction mixture was cooled to 0 °C and quenched with 7 mL MeOH drop wise and stirred for 30 min. subsequently, 10 mL 1 N HCl was added, and the reaction mixture was distilled at 100 °C for 1 h. The reaction mixture was then cooled to 0 °C and made basic (>10.5) with NH_4_OH and extracted with 9:1 CHCl_3_: MeOH. The combined organic layers were washed with water and brine, dried with sodium sulfate and concentrated. Purification by silica gel column chromatography 0–60% EtOAc: Hexanes. **26** was isolated as a white foam (347 mg, 33% yield) as the more polar fraction. The HCl salt of **26** was formed in *i*PrOH (1 mL) with 37% HCl (0.1 mL) and recrystallized from hot ethanol (4 mL) to give a white solid: mp 259–262 °C. ^1^H-NMR (400 MHz; CD_3_OD): δ 7.35 (t, *J* = 6.5 Hz, 4H), 7.28 (dq, *J* = 8.6, 4.3 Hz, 1H), 7.10 (t, *J* = 7.9 Hz, 1H), 6.84–6.81 (m, 2H), 6.61 (dd, *J* = 8.0, 2.3 Hz, 1H), 5.43 (dt, *J* = 10.9, 7.2 Hz, 1H), 5.32 (dd, *J* = 10.7, 9.3 Hz, 1H), 3.68–3.62 (m, 3H), 3.59 (d, *J* = 13.4 Hz, 1H), 3.49 (t, *J* = 8.4 Hz, 2H), 3.15 (dd, *J* = 10.3, 5.8 Hz, 2H), 2.38–1.93 (m, 10H), 0.97 (t, *J* = 7.5 Hz, 3H). ^13^C NMR (101 MHz; CD_3_OD): δ 158.5, 149.8, 137.7, 137.1, 130.2, 129.9, 129.9, 128.3, 125.6, 117.8, 114.2, 113.9, 61.6, 56.8, 51.1, 42.3, 39.7, 37.8, 31.7, 29.1, 22.2, 21.1, 18.6, 14.3. HRMS-ESI (*m*/*z*): [M + H^+^] calcd. for C_26_H_34_NO 376.2640; found 376.2642; Anal. calcd. for C_26_H_34_ClNO: C, 75.79%; H, 8.32%, N, 3.4%. Found C_26_H_34_ClNO: C, 75.89%; H, 8.18%; N, 3.47%; [a]^20^_D_° 4.1 (c 1.08, CHCl_3_).

*3-((1S,5R,9R)-9-((Z)-But-1-en-1-yl)-2-phenethyl-2-azabicyclo [3.3.1]nonan-5-yl)phenol* (**27**). From the same reaction as **26**, **27** was isolated as a white foam (294 mg, 28% yield) as the less polar fraction. The HCl salt of **27** was formed in *i*PrOH (1 mL) with 37% HCl (0.1 mL) and recrystallized from hot ethanol (4 mL) to give a white solid: mp 264–267 °C. ^1^H-NMR (400 MHz; CD_3_OD): δ 7.37–7.26 (m, 5H), 7.10 (t, *J* = 8.0 Hz, 1H), 6.75 (d, *J* = 7.9 Hz, 1H), 6.71 (s, 1H), 6.61 (dd, *J* = 8.0, 2.1 Hz, 1H), 5.59–5.53 (m, 1H), 5.30 (dd, *J* = 10.7, 9.4 Hz, 1H), 3.76–3.68 (m, 2H), 3.56 (dt, *J* = 13.6, 7.0 Hz, 2H), 3.49–3.47 (m, 1H), 3.38 (td, *J* = 12.1, 5.2 Hz, 1H), 3.13 (td, *J* = 12.1, 5.5 Hz, 1H), 2.92 (ddd, *J* = 12.3, 11.6, 5.3 Hz, 1H), 2.60–2.51 (m, 1H), 2.42–2.35 (m, 2H), 2.28–1.83 (m, 7H), 1.04 (t, *J* = 7.5 Hz, 3H). ^13^C NMR (101 MHz; CD_3_OD): δ 158.5, 150.0, 138.5, 137.4, 130.3, 130.0, 129.9, 128.4, 125.9, 117.6, 114.1, 113.7, 60.9, 56.5, 51.8, 42.7, 42.1, 37.8, 31.5, 29.0, 23.9, 22.16, 22.08, 14.2. HRMS-ESI (*m*/*z*): [M + H^+^] calcd. for C_26_H_34_NO 376.2640; found 376.2644; Anal. calcd. for C_26_H_34_ClNO·0.1 H_2_O: C, 75.46%; H, 8.33%; N, 3.38%; Found C, 75.47%; H, 8.2%; N, 3.28%; [a]^20^_D_° 12.9 (c 0.79, CHCl_3_).

### 3.3. Molecular Modeling of the Mouse MOR with C9-Alkenyl Compounds

Both active (in complex with Gi protein and peptide agonist DAMGO, PDB 6DDF [21] and inactive (in complex with morphinan antagonist β-funaltrexamine, PDB 4DKL [22] µ-opioid receptor (MOR) structures were obtained from Protein Data Bank. Compounds **8**, **9**, **14** and **15** (Table 2) were prepared using Ligprep (Release 2021-3, Schrodinger, LLC, New York, NY, USA) and the nitrogen of the piperidine ring was protonated. 4DKL and 6DDF were processed using the Protein Preparation Wizard (Release 2021-3, Schrodinger, LLC, New York, NY, USA). All water molecules were removed from the system. Hydrogen bonds were assigned using PROPKA at pH 7.0, and D114^2.50^ and D164^3.49^ were protonated according to previous studies [23]. After the receptors were processed, they were minimized with Prime using the OPLS4 force field.

The prepared receptor structures were used to dock ligands via induced-fit docking (IFD) implemented in Schrodinger (Release 2021-3, Schrodinger, LLC, New York, NY, USA). The centroid of the docking box was determined by the position of the bound ligand in each structure. The standard precision protocol of IFD was used, and up to 20 poses were generated for each ligand. An additional hydrogen-bond constraint was added to ensure that the docking poses maintained a conserved ionic interaction with D147^3.32^. For each ligand, the resulting docking poses were clustered using K-means clustering, and the representative pose was selected from among the best scored poses of the largest cluster.

For the C9-propenyl compounds **17**, **18**, **20**, and **21** compounds, an additional methyl moiety was grafted onto the vinyl group to generate a propenyl group in the *Z*-isoform of the **8**, **9**, **15** and **14** representative poses, respectively

#### MM/GBSA Calculations

Molecular mechanics generalized Born surface area calculations (MM/GBSA) were performed using a VSGB solvation model with internal and solvent dielectric constants of 1.0 and 80.0, respectively. The energy minimization and MM/GBSA calculation for each system were calculated with Prime of Schrodinger (Release 2021-3, Schrodinger, LLC, New York, NY, USA).

### 3.4. In Vivo Activity in Nonhuman Primates

#### 3.4.1. Antinociception: Warm-Water Squirrel Tail-Withdrawal Method

Male squirrel monkeys (Saimiri sciureus) were housed in a climate-controlled vivarium with a 12 h light/dark cycle (7 AM–7 PM) in the McLean Hospital Animal Care Facility (licensed by the U.S. Department of Agriculture and compliant with guidelines provided by the Committee on Care and Use of Laboratory Animals of the Institute of Laboratory Animals Resources, Commission on Life Sciences, National Research Council; 2011). Tail withdrawal latencies were assessed as described previously [24]. Briefly, monkeys were seated in customized Plexiglas chairs that allowed their tails to hang freely. Tail withdrawal latencies were measured by immersing the subject’s tail in water held at 35 or 52 °C (temperatures were presented in a randomized order during successive test components). After obtaining a baseline tail withdrawal latency, complete dose response curves were generated in each subject using standard cumulative dosing procedures. Briefly, every 15 min after an injection tail-withdrawal latencies at each temperature were redetermined and subjects were injected with the next dose, such that the total (cumulative) dose was increased by ½ log_10_ units in each successive cycle. This procedure was repeated until either (a) the tail-withdrawal latency from 52 °C water reached the maximum allowable latency (10 sec), or (b) tail-withdrawal latency no longer increased with increases in dose of the test drug.

#### 3.4.2. Respiratory Depression: Ventilatory Response to Hypercapnia (5% CO_2_ in Air)

Ventilation measures were assessed as described previously [25]. Briefly, squirrel monkeys were acclimated to a customized acrylic chamber (10″ d × 10″ w × 10″ h) that served as a whole-body plethysmograph (EMKA Technologies, Montreal, PQ, Canada). Gas (either air or a 5% CO_2_ in air mixture) was introduced to and extracted from the chamber at a constant flow rate of 5 L/min. Experimental sessions consisted of 4–6 consecutive 30 min cycles, each comprising a 20 min exposure to air followed by a 10 min exposure to 5% CO_2_. Drug effects were determined using cumulative dosing procedures, and injections were administered following each exposure to 5% CO_2_. Respiratory rate and tidal volume (mL/breath) were recorded over 1 min periods and were multiplied to provide minute volumes. Data from the last three minutes of each exposure to air or CO_2_ were averaged and used for analysis of drug effects on ventilation.

#### 3.4.3. Data Analysis

All statistical analyses and graphic representations were completed with GraphPad Prism version 9.3.0 (GraphPad Software, San Diego, CA, USA) using log transformed values of doses. Group means ± SEM tail withdrawal latencies (in sec) and minute volume ratios are plotted as a function of drug dose. Data were analyzed using One-way ANOVA with significance set at *p* < 0.05, followed by Dunnett’s multiple comparison test. Animals that did not receive all doses of a drug in tail withdrawal studies because they attained a maximum effect at less than the highest dose were assigned 10 s latencies for all doses higher than the last dose tested.

### 3.5. In Vitro Assays

In vitro binding assays were performed using monocloned mouse mu opioid receptor expressing Chinese hamster ovary (CHO) cells (mMOR-CHO). mMOR-CHO cell culture and membrane homogenate preparation were performed as previously described [26]. All assays were duplicated and repeated at least three times.

#### 3.5.1. Competition Radioligand Binding Assay

Competition binding assays were performed as previously described [26,27]. Briefly, mMOR-CHO membrane homogenates containing 20 µg membrane protein were incubated with 1.4 nM [^3^H]naloxone in the presence and absence of varying concentrations of test compounds in TME buffer (50 mM Tris, 3 mM MgCl_2_, and 0.2 mM EGTA, pH 7.7) for 1.5 h at 30 °C. Bound radioactive ligand was isolated by filtration through GF/B glass fiber filters and rinsed three times with ice-cold wash buffer (50 mM Tris-HCl, pH 7.2). Bound radioactivity was determined via liquid scintillation ounting. Specific binding was determined as the difference in binding obtained in the absence and presence of 5 µM naltrexone. The IC_50_ values were determined by nonlinear regression analysis using GraphPad Prism 9 (GraphPad Software, San Diego, CA, USA) and converted to K_i_ values using the Cheng–Prusoff equation.

#### 3.5.2. [^35^S]GTPγS Functional Assay

[^35^S]GTPγS functional assays were performed as described [26,27]. Briefly, mMOR-CHO membrane homogenates containing 14 µg of membrane protein were incubated in TME buffer with 100 mM NaCl, 20 µM GDP, 0.1 nM [^35^S]GTPγS with and without varying concentrations of test compounds in a final volume of 500 µL for 1.5 h at 30 °C. In addition, 3 µM of DAMGO was included as a reference point for a maximally effective concentration of a full MOR agonist. Bound [^35^S]GTPγS was isolated by filtration as described above, and radioactivity was determined via scintillation counting. Basal binding was determined in the absence of agonist and non-specific binding was determined with 10 µM GTPγS. Net-stimulated [^35^S]GTPγS binding was defined as specific agonist-stimulated minus specific basal binding. Data were normalized as % of maximal DAMGO stimulation, defined as (net-stimulated binding by ligand/net-stimulated binding by 3 µM DAMGO) ×100%. Concentration-effect data were fit by non-linear regression to determine E_max_ and EC_50_ values using GraphPad Prism 9 (GraphPad Software, San Diego, CA, USA).

#### 3.5.3. Forskolin-Induced cAMP Accumulation Assays

Cell lines and cell culture: cAMP Hunter^TM^ Chinese hamster ovary cells (CHO-K1) that express human μ-opioid receptor (OPRM1), human κ-opioid receptor (OPRMK1), and human δ-receptor (OPRMD1) were purchased from Eurofins DiscoverX (Fremont, CA) and used for the forskolin-induced cAMP accumulation assays [28].

Briefly, cells were plated at 10,000 cells/well density in a 384-well tissue culture plate and incubated overnight at 37 °C in 5% CO_2_. Stock solutions of compound were made in 100% DMSO at a 5 mM concentration. A serial dilution of 10 concentrations was made using 100% DMSO, creating 100× solutions of the compound for treatment. The 100× solutions were then diluted to 5× solutions using assay buffer consisting of Hank’s Buffered Salt Solution, HEPES, and forskolin. For the agonist assay, cells were incubated at 37 °C with compounds for 30 min at a 1× concentration. For the antagonist assay, cells were incubated at 37 °C with compounds for 15 min before 30-min incubation at 37 °C with selected agonist at their EC_50_ or EC_90_ dose. The HitHunter cAMP Assay for Small Molecules by DiscoverX was then used according to manufacturer’s directions and the BioTek Synergy H1 hybrid plate reader (BioTek, Winooski, VT, USA) and Gen5 Software version 2.01 (were used to quantify luminescence (BioTek, Winooski, VT, USA) [17].

## 4. Conclusions

Three MOR antagonists were found to be as or more potent than naltrexone and, unlike naltrexone, none of them had MOR, KOR, or DOR agonist activity. Several potent MOR full agonists were obtained, and, of particular interest partial agonists were found that exhibited less respiratory depression than that caused by morphine. The effect of stereochemistry and the length of the C9-alkenyl chain was also explored using molecular modeling. The MOR antagonists were found to interact with the inactive (4DKL) MOR crystal structures and agonists were found to interact with the active (6DDF) MOR crystal structures. The agonists and antagonists could, thus, be differentiated through molecular modeling.

## 5. Patents

A patent application (US Patent Application Serial No. 63/393,035, filed 28 July 2022, “Selective Opioid Receptor Agonists and Antagonists”) has been filed by K. C. Rice. A. E. Jacobson, A. Sulima, and D. C. Chambers.

## Figures and Tables

**Table 1:** Varying concentration and time of acid hydrolysis to give different ratios of epimeric products, as determined from NMR.

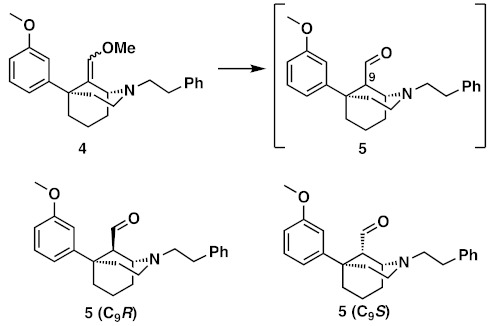		
Acid.	Time	C_9_*R*:C_9_*S* Ratio (via NMR)
3NHCl	17 h	1:9
6NHCl	4 h	1:2
6NHCl	17 h	1:1.3

**Table 2:** Opioid Receptor Activity Measured in the Forskolin-induced cAMP Accumulation Assay ^a^.

		MOR		DOR		KOR	
		Agonist	Antagonist ^b^	Agonist	Antagonist ^c^	Agonist	Antagonist ^d^
Name	Structure	EC_50_ ± SEM (nM) (%E_max_ ± SEM)	IC_50_ ± SEM (nM) (%I_max_ ± SEM)	EC_50_ ± SEM (nM) (%E_max_ ± SEM)	IC_50_ ± SEM (nM) (%I_max_ ± SEM)	EC_50_ ± SEM (nM) (%E_max_ ± SEM)	IC_50_ ± SEM (nM) (%I_max_ ± SEM)
	**Set 1- C9-Vinyl**						
DC-01-0076.2 **8**	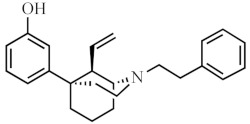	1.44 ± 0.48 (94.7 ± 3.1%)	N/D	>10,000	112.9 ± 43.6 (119.1 ± 15.5%)	>10,000	74.0 ± 30.5 (97.1 ± 9.2%)
DC-01-0076.1 **9**	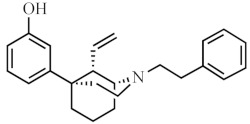	2.12 ± 0.45 (67.3 ± 6.8%)	45.6 ± 17.3 (18.8 ± 3.8%)	57.0 ± 21.4 (22.6 ± 3.3%)	N/D	>10,000	19.8 ± 9.7 (101.8 ± 19.1%)
DC-01-0102.2 **14**	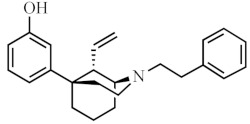	3.94 ± 0.85 (46.6 ± 9.8%)	50.6 ± 12.1 (70.6 ± 8.0%)	121.8 ± 47.2 (40.6 ± 6.4%)	N/D	>10,000	128.1 ± 48.2 (105.6 ± 4.6%)
DC-01-0102.1 **15**	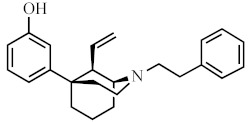	>10,000	3.58 ± 0.72 (130.8 ± 4.5)	>10,000	143.7 ± 41.0 (201.4 ± 35.9%)	>10,000	28.4 ± 4.2 (104.9 ± 11.2%)
	**Set 2—C9-Propylene**						
DC-01-0090.2 **17**	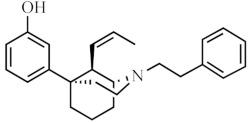	2.61 ± 0.64 (89.5 ± 2.9%)	N/D	>10,000	113.2 ± 19.8 (143.3 ± 11.9%)	>10,000	35.0 ± 9.7 (111.6 ± 7.9%)
DC-01-0128.1 **18**	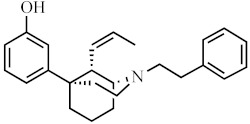	0.07 ± 0.02 (101 ± 0.2%)	N/D	9.69 ± 2.23 (74.5 ± 2.3%)	N/D	>10,000	237.7 ± 52.6 (114.8 ± 11.2%)
DC-01-0155 **20**	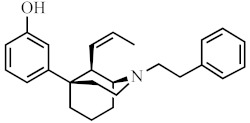	>10,000	2.34 ± 0.20 (157.1 ± 4.0%)	>10,000	7.52 ± 2.93 (142.5 ± 23.7%)	>10,000	4.02 ± 0.25 (126.7 ± 2.6%)
DC-01-0095 **21**	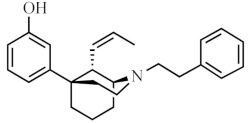	4.66 ± 0.68 (57.5 ± 7.9%)	45.6 ± 6.7 (39.8 ± 3.9%)	60.5 ± 3.7 (66.7 ± 4.4%)	N/D	>10,000	162.6 ± 37.0 (111.8 ± 2.2%)
	**Set 3—C9-Butylene**						
DC-01-0130.2 **23**	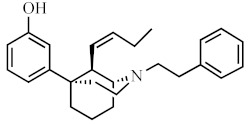	11.6 ± 1.8 (95.8 ± 1.6%)	N/D	>10,000	69.0 ± 4.8 (166.6 ± 40.0%)	>10,000	328.3 ± 96.9 (109.5 ± 8.3%)
DC-01-0116.1 **24**	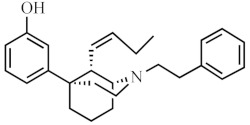	14.7 ± 3.6 (89.8 ± 1.9%)	N/D	>10,000	3224± 572 (182.3 ± 56.4%)	>10,000	282.7 ± 46.4 (103.8 ± 9.2%)
DC-01-0104.2 **26**	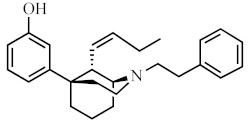	>10,000	20.0 ± 10.7 (129.4 ± 3.4%)	>10,000	520.4 ± 143.9 (226.1 ± 14.0%)	>10,000	12.2 ± 2.5 (96.2 ± 0.9%)
DC-01-0104.1 **27**	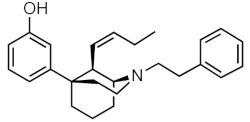	>10,000	2.37 ± 0.47 (167.8 ± 10.6%)	>10,000	8.22 ± 2.44 (211.5 ± 24.7%)	>10,000	0.79 ± 0.28 (98.2 ± 2.3%)
	**Miscellaneous—Methoxy**						
DC-01-0125 **13**	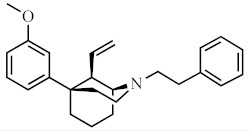	>10,000	2716 ± 1098 (128.5 ± 16.6%)	>10,000	>10,000	>10,000	>10,000
	**Standards**						
	Morphine	6.28 ± 0.43 (102.1 ± 0.2%)					
	Naltrexone	2.14 ± 1.2 (29.6 ± 6.4%)	10.8 ± 1.0 (103.5 ± 0.6%)	>10,000	295.1 ± 47.5 (99.4 ± 1.1%)	0.64 ± 0.32 (56.5 ± 7.2%)	5.53 ± 1.02 (41.3 ± 6.8%)

^a^ Inhibition of forskolin-induced cAMP accumulation; cAMP Hunter^TM^ Chinese hamster ovary cells (CHO-K1) that express human μ-opioid receptor (OPRM1), human κ-opioid receptor (OPRK1), and human δ-opioid receptor (OPRD1) were used for the forskolin-induced cAMP accumulation assay to determine potency and efficacy of the compounds following the previously established methods; [17] to determine % efficacy in forskolin-induced cAMP assays, data were blank subtracted with the vehicle control, followed by normalization to the forskolin control. Data were then analyzed in GraphPad Prism 8 (GraphPad, LaJolla, CA, USA) using nonlinear regression; values are expressed as the mean ± SEM of at least three independent experiments; N/D = not determined. ^b^ MOR Antagonist potency (IC_50_) determined versus EC90 of fentanyl; Degree of antagonism (I_max_) normalized to naltrexone. ^c^ DOR Antagonist potency (IC_50_) determined versus EC50 of SNC80; degree of antagonism (I_max_) normalized to naltrexone. ^d^ KOR Antagonist potency (IC_50_) determined versus EC90 of U50488H; degree of antagonism (Imax) normalized to *nor*-BNI.

**Table 3:** MOR radioligand binding assay (K_i_, nM) and MOR [^35^S]GTPgS functional assay ^a^.

Compounds	Molecular Structure	[^3^H]NLX Binding K_i_ (nM)	GTPgS % E_max_ of (DAMGO)	GTPgS EC_50_ (nM)
DC-01-0076.2 **8**	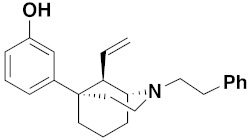	0.50 ± 0.05	20.09 ± 0.78	6.73 ± 1.35
DC-01-0076.1 **9**	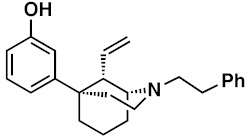	1.91 ± 0.13	10.54 ± 0.82	36.97 ± 15.47
DC-01-0090.2 **17**	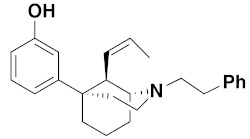	1.83 ± 0.24	17.97 ± 1.45	9.64 ± 1.70
DC-01-0128.1 **18**	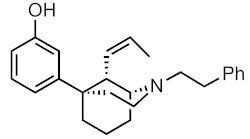	0.56 ± 0.07	75.35 ± 3.83	8.38 ± 0.77
Morphine		1.20 ± 0.16	88.30 ± 4.86	123.0 ± 23.56

^a^ The values are the mean ± SEM of three independent experiments. Membranes for all radioligand binding assays were prepared from mMOR-CHO cells.

**Table 4:** MM/GBSA calculations for C9-substituted compounds.

Ligand	Ligand Type	ΔG_Bind_ (A) (kcal/mol)	ΔG_Bind_ (I) (kcal/mol)	ΔΔG_Bind_ (A − I) (kcal/mol)
**8**	Agonist	−71.1	−60.8	−10.3
**9**	Agonist	−69.7	−62.5	−7.2
**14**	Agonist	−65.3	−61.7	−3.6
**15**	Antagonist	−47.3	−70.5	23.2
**17**	Agonist	−70.7	−61.9	−8.8
**18**	Agonist	−72.3	−66.1	−6.2
**21**	Agonist	−68.8	−60.2	−8.6
**20**	Antagonist	−47.6	−70.5	22.9

ΔΔG_Bind_ measures the difference between ΔG_Bind_ in the active (A) model and ΔG_Bind_ in the inactive (I) model. The antagonists bind most favorably to the inactive receptor, and the agonists bind most favorably to the active receptor.

## Data Availability

The publicly available Protein Data Bank was used to obtain opioid receptor structures for molecular modeling, and the publicly available Cambridge Structural Database was used to store the crystal structures for compounds **8** and **20**.

## Supplementary Materials

The following are available at https://www.mdpi.com/article/10.3390/molecules27196455/s1, ^1^H and ^13^C-NMR spectra of novel compounds, crystal data, atomic coordinates, etc., for compounds **8** and **20**.

## Abbreviations

MOR, μ	Mu-opioid receptor
DOR, δ	Delta-opioid receptor
KOR, κ	Kappa-opioid receptor
cAMP	cyclic adenosine monophosphate
DAMGO, [D-Ala2, N-Me-Phe4, Gly5-ol]	enkephalin
GTPγS	guanosine-5′-O-thio-triphosphate
CHO	Chinese hamster ovary
PDB	Protein Data Base
