# Supplementary material for: A Journey through Diastereomeric Space: The Design, Synthesis, In Vitro and In Vivo Pharmacological Activity, and Molecular Modeling of Novel Potent Diastereomeric MOR Agonists and Antagonists
Source: Molecules. 2022 Sep 30;27(19):6455. doi: 10.3390/molecules27196455 (PMC9570967; doi:10.3390/molecules27196455)
Supplement: Supplementary file 1 [file molecules-27-06455-s001.zip › molecules-1917474-supplementary.pdf]

SUPPLEMENTARY MATERIALS

A Journey Through Diastereomeric Space: The Design, Synthesis, In Vitro and In Vivo Pharmacological Activity, and Molecular Modeling of Novel Potent Diastereomeric MOR Agonists and Antagonists

Dana R. Chambers [1], Agnieszka Sulima [1], Dan Luo [2], Thomas E. Prisinzano [2], Alexander Goldberg [3], Bing Xie [3], Lei Shi [3], Carol A. Paronis [4], Jack Bergman [4], Nima Nassehi [5], Dana E. Selley [5], Gregory H. Imler [6], Arthur E. Jacobson [1,*] and Kenner C. Rice [1,*]

[1] Drug Design and Synthesis Section, Molecular Targets and Medications Discovery Branch, Intramural Research Program, National Institute on Drug Abuse and the National Institute on Alcohol Abuse and Alcoholism, National Institutes of Health, Department of Health and Human Services,
9800 Medical Center Drive, Bethesda, MD 20892, USA

[2] Department of Pharmaceutical Sciences, College of Pharmacy, University of Kentucky, 789 S. Limestone Street, Lexington, KY 40536, USA

[3] Computational Chemistry and Molecular Biophysics Section, Molecular Targets and Medications Discovery Branch, Intramural Research Program, National Institute on Drug Abuse, National Institutes of Health, Department of Health and Human Services, 333 Cassell Drive, Baltimore, MD 21224, USA

[4] McLean Hospital, Harvard Medical School, 115 Mill Street, Belmont MA 02478, USA;

[5] Department of Pharmacology and Toxicology, Virginia Commonwealth University, 1112 East Clay Street, Richmond, VA 23298, USA

[6] Naval Research Laboratory, Center for Biomolecular Science and Engineering, Washington, DC 20375, USA;

* Correspondence: arthurj@nida.nih.gov (A.E.J.); kennerr@nida.nih.gov (K.C.R.); Tel.: +1-301-451-5028 (A.E.J.); +1-301-451-4799 (K.C.R.)

Table Sof Contents **Page #**

1) ^{1}H (top of page) & ^{13}C NMR (bottom of page)	3–18
2) Tables S1 – S7. Crystal data, atomic coordinates, etc, for compound **8**	19–27
3) Tables S1 – S7. Crystal data, atomic coordinates, etc, for compound **20**	28–36

Compound **6**.Oxalate

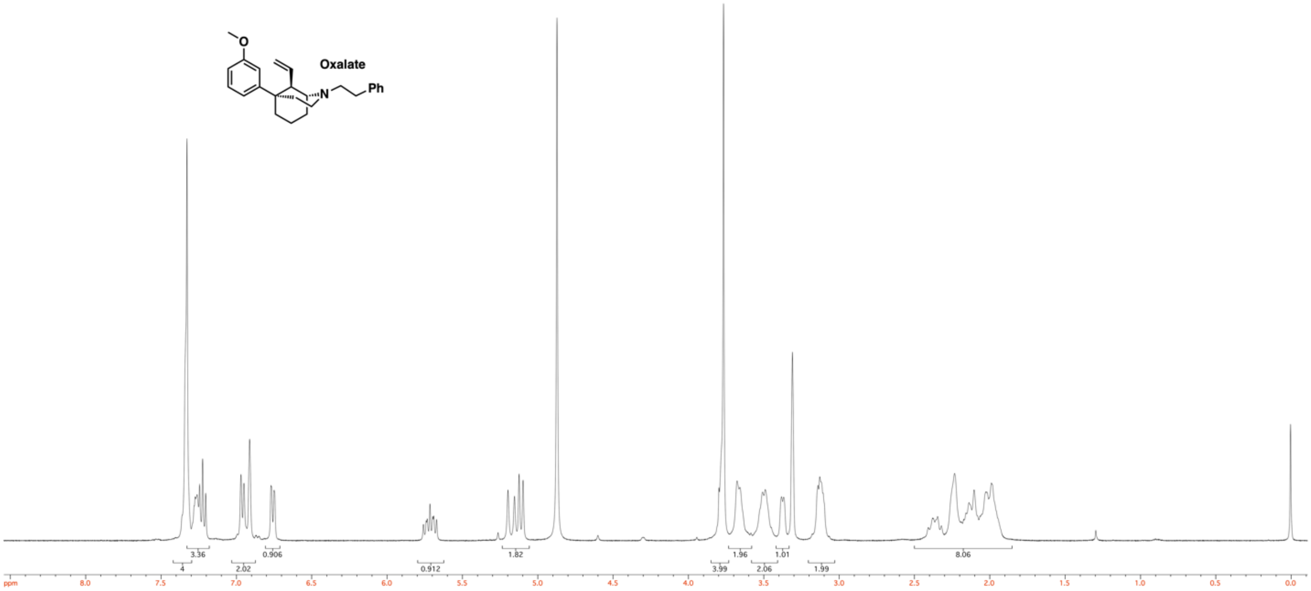

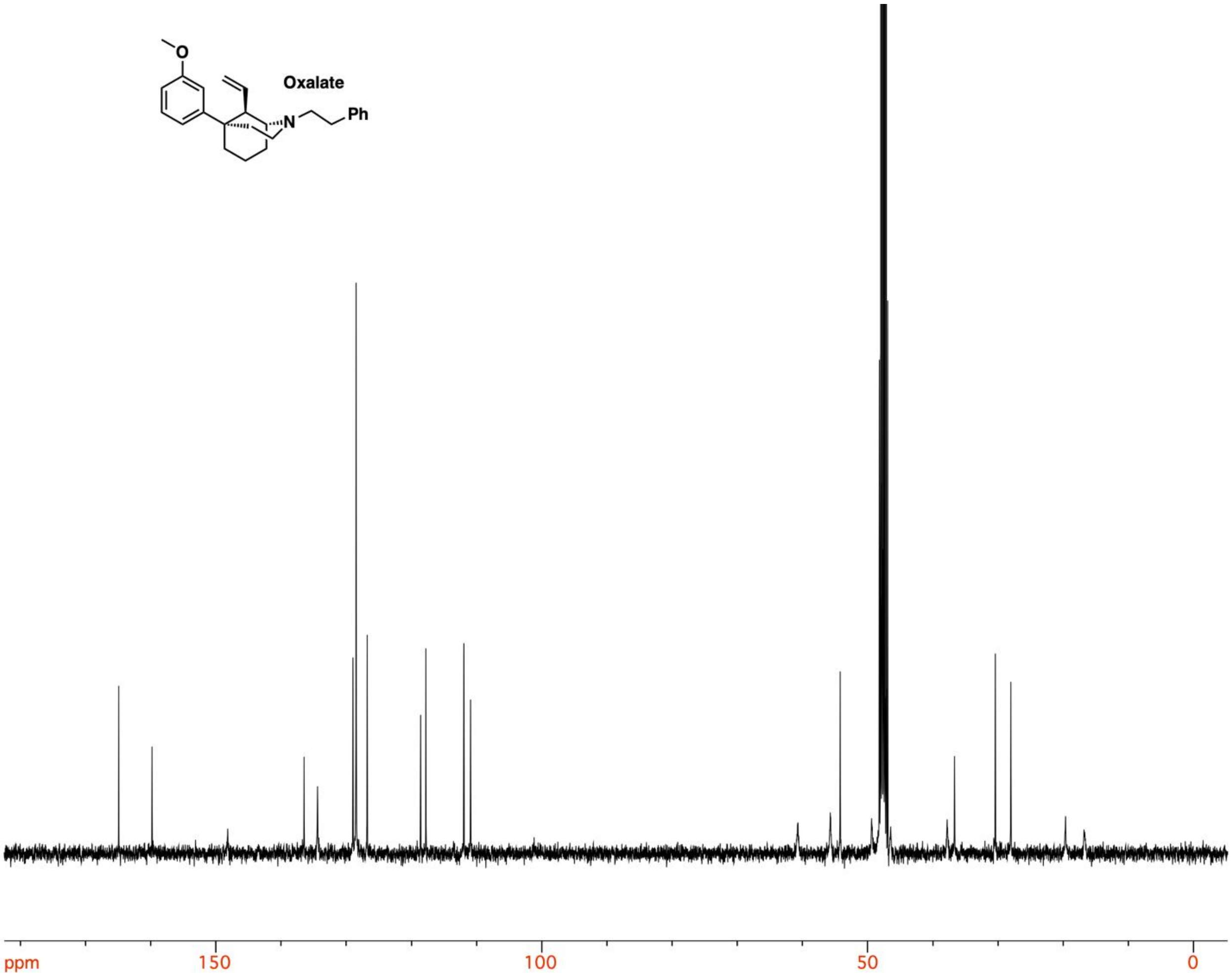

Compound **7**.oxalate

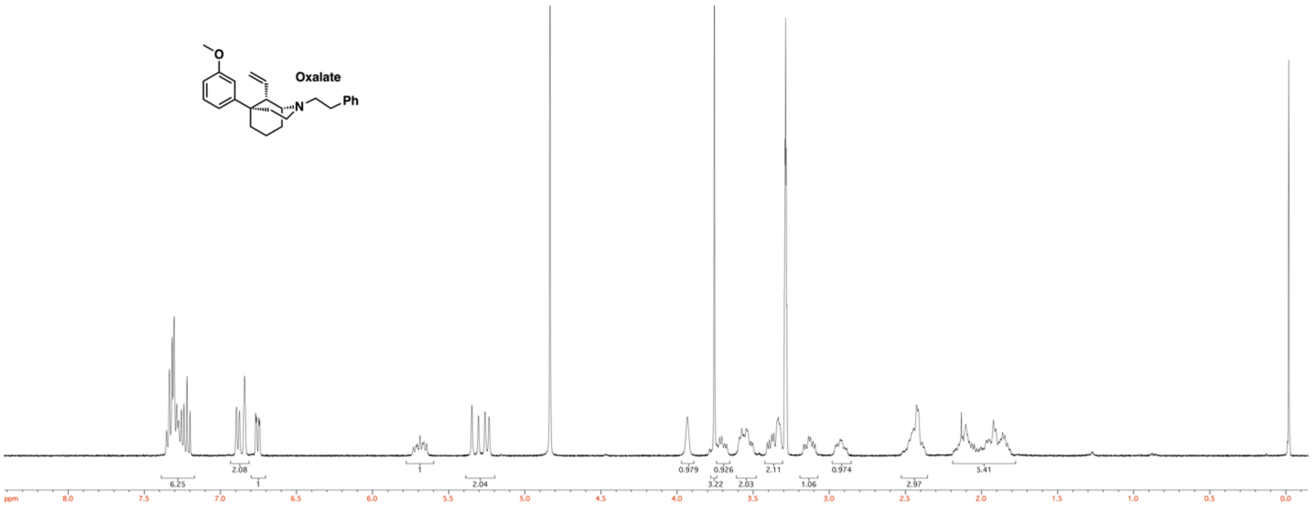

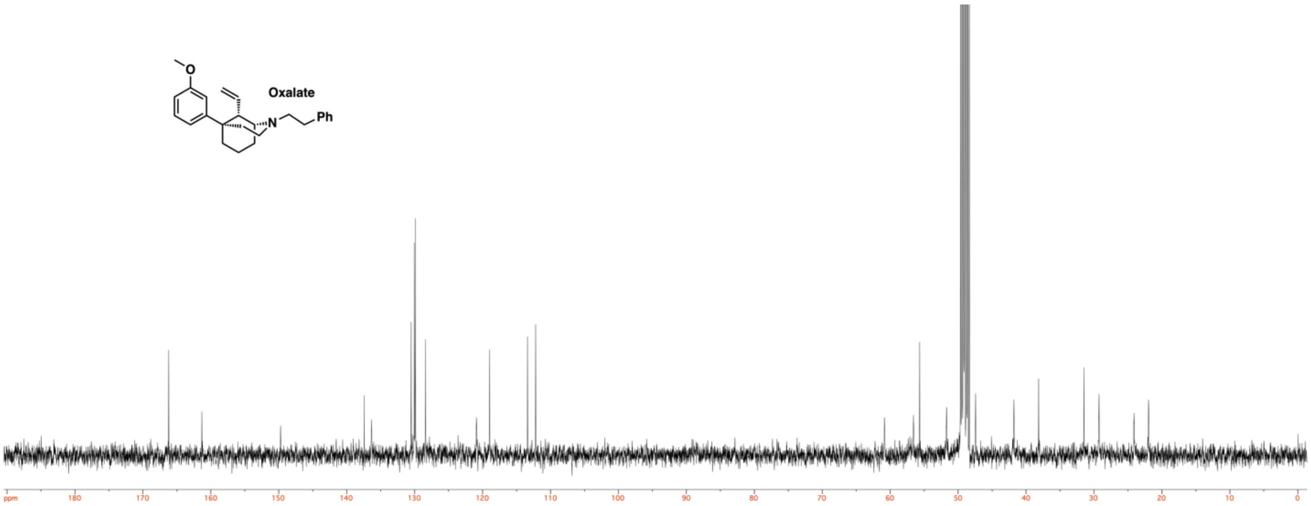

Compound **8**

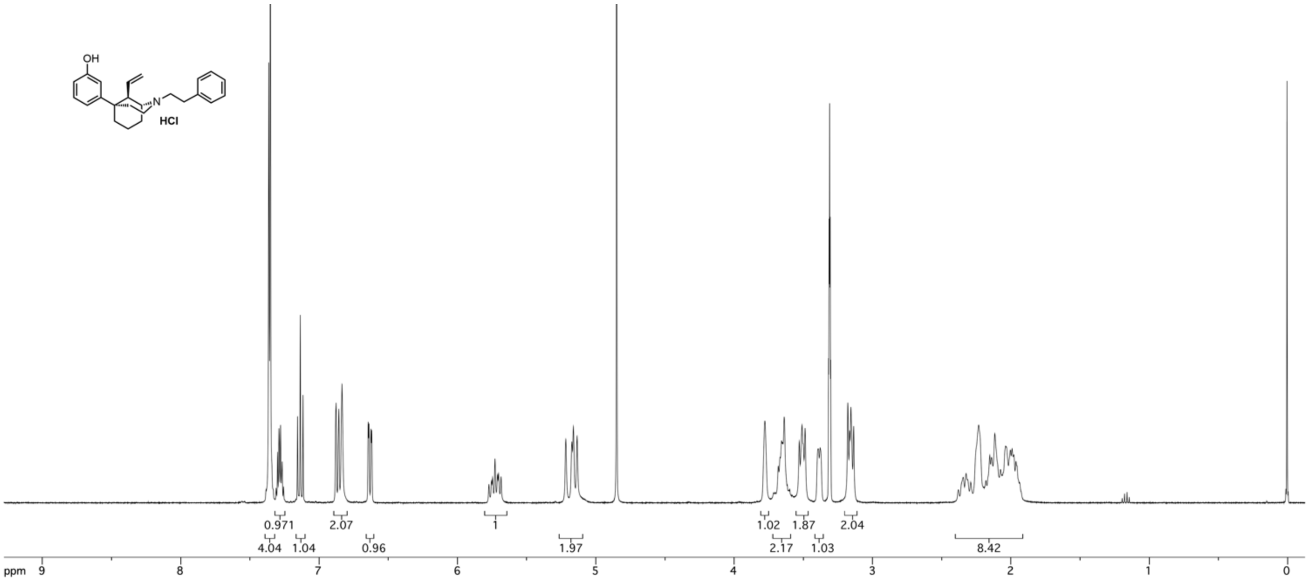
OH
N
HCl
0.971
2.07
1
1.02
1.87
2.04
4.04
1.04
0.96
1.97
2.17
1.03
8.42
ppm 9 8 7 6 5 4 3 2 1 0

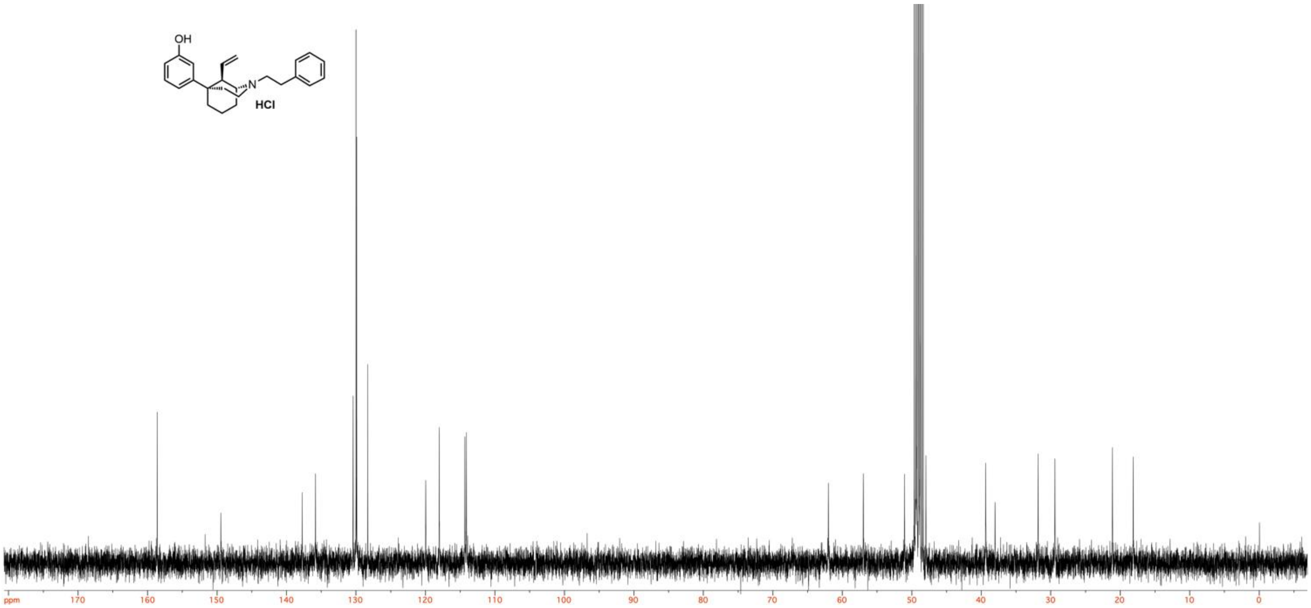
OH
N
HCl
ppm 170 160 150 140 130 120 110 100 90 80 70 60 50 40 30 20 10 0

Compound **9**.

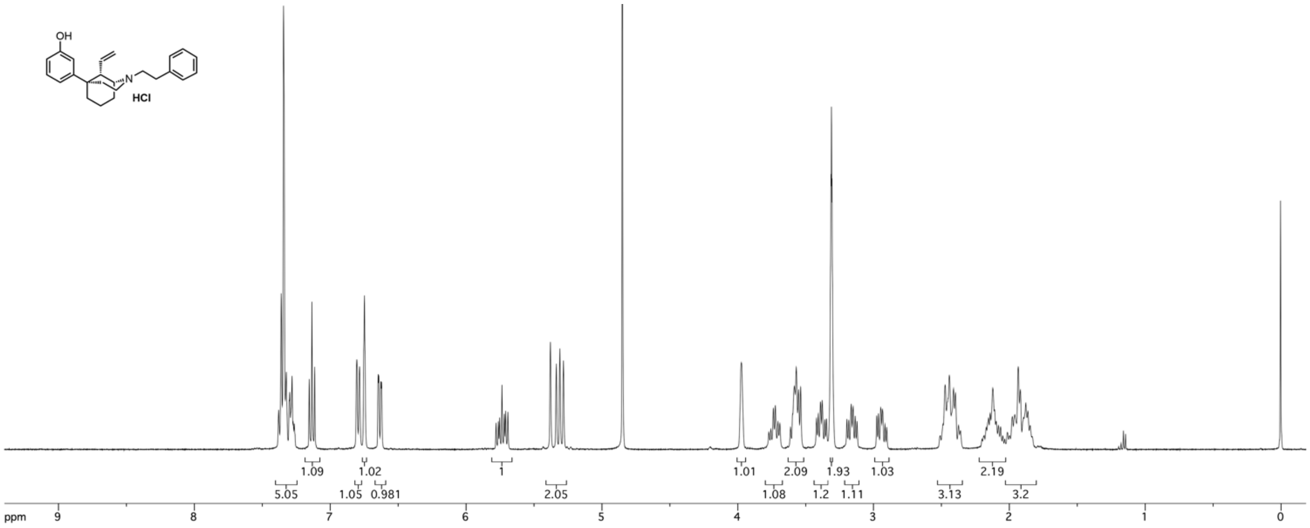
OH
N
HCl
1.09
1.02
1
1.01
2.09
1.93
1.03
2.19
5.05
1.05
0.981
2.05
1.08
1.2
1.11
3.13
3.2
ppm
9
8
7
6
5
4
3
2
1
0

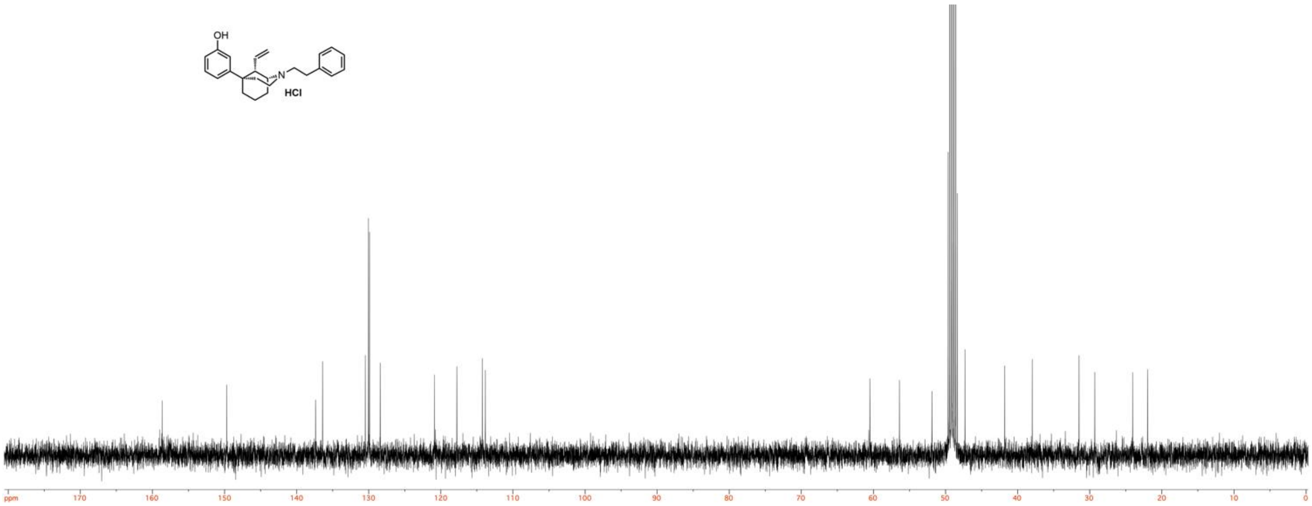
OH
N
HCl

Compound **12.oxalate**

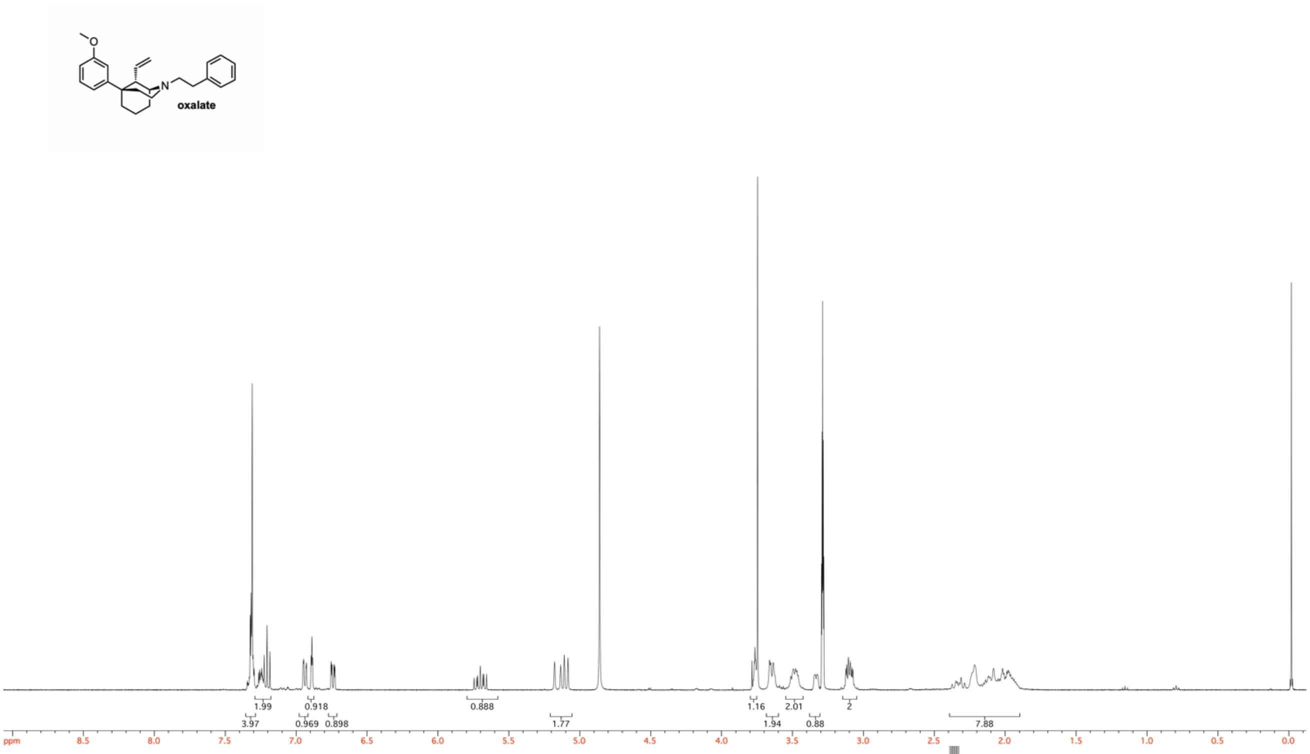

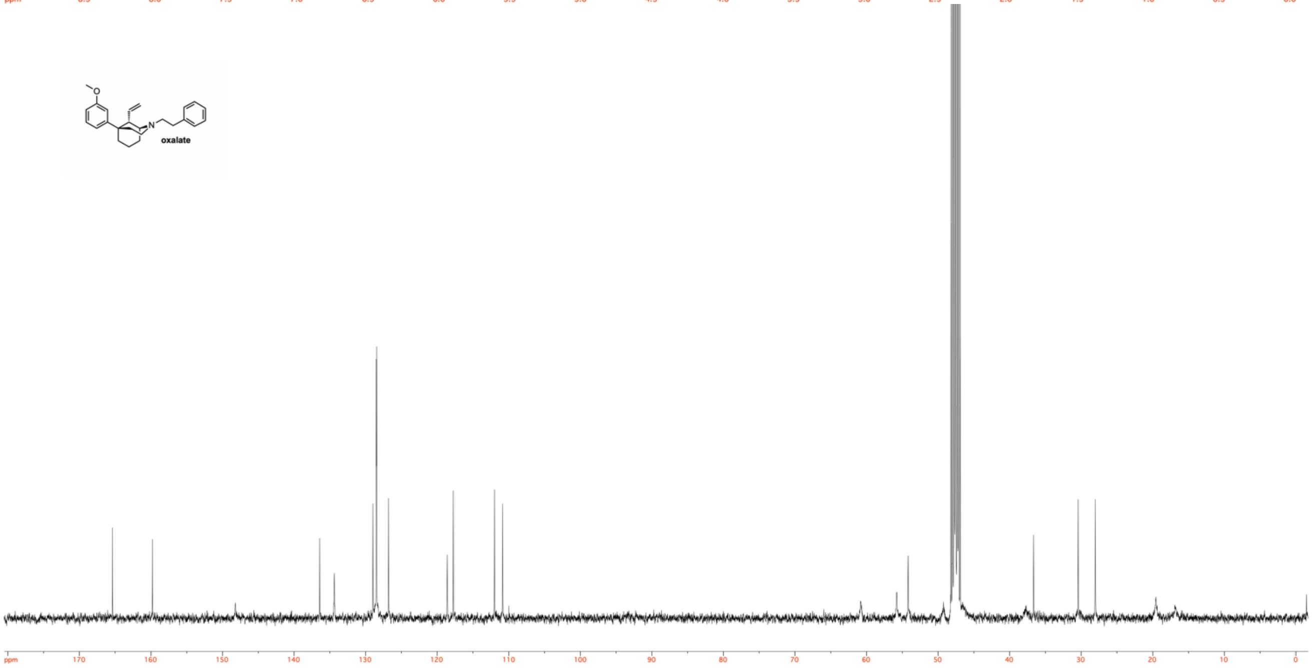

Compound **13.oxalate**

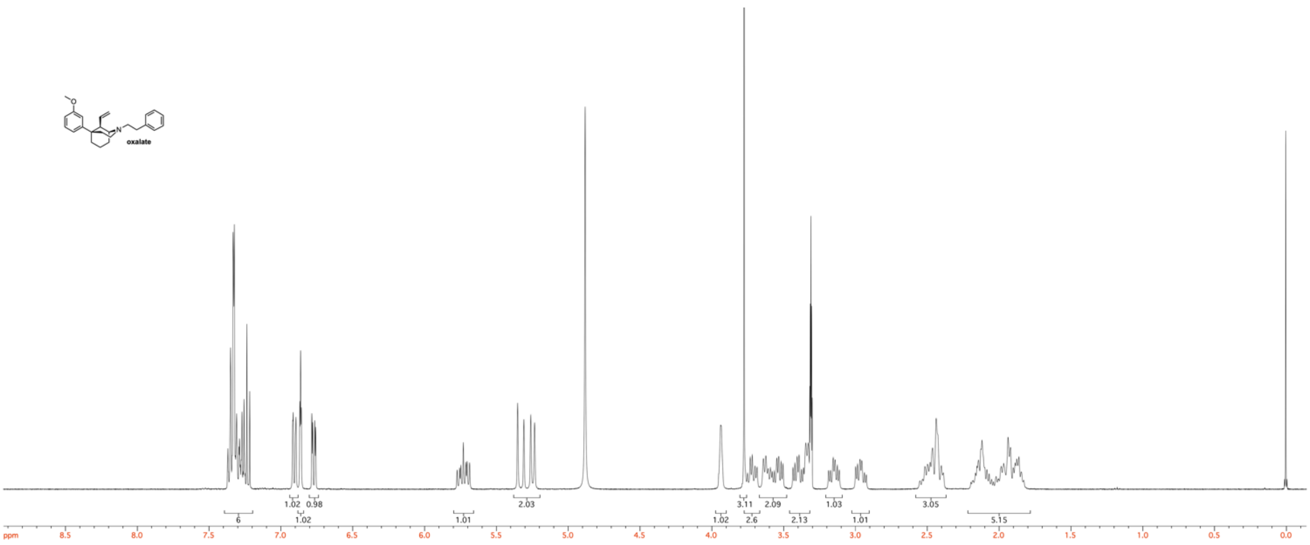

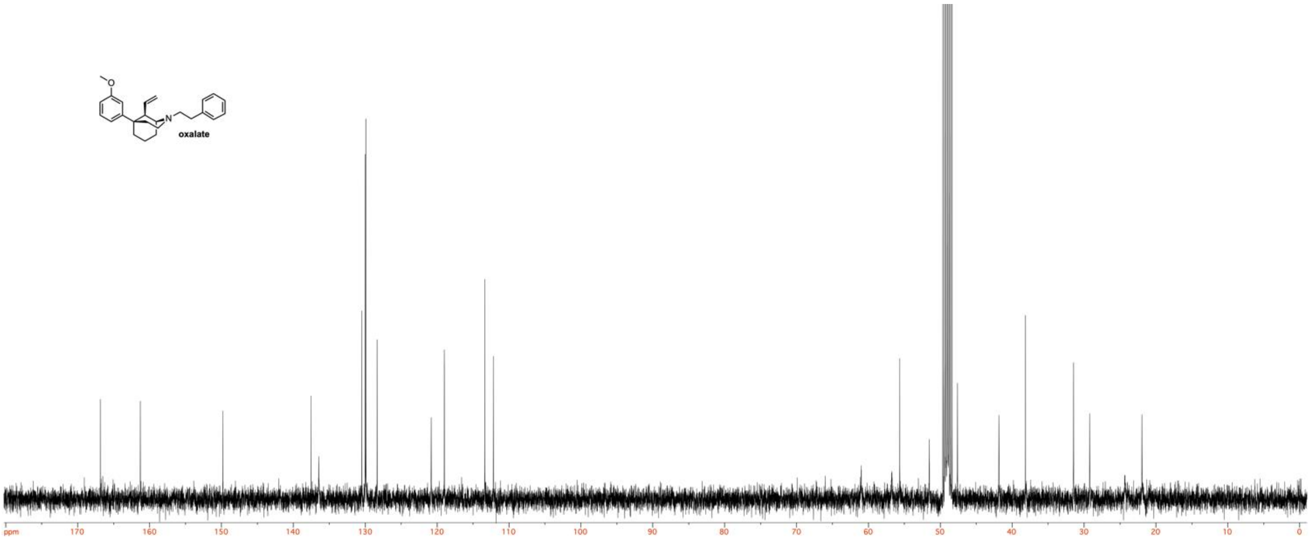

Compound **14**

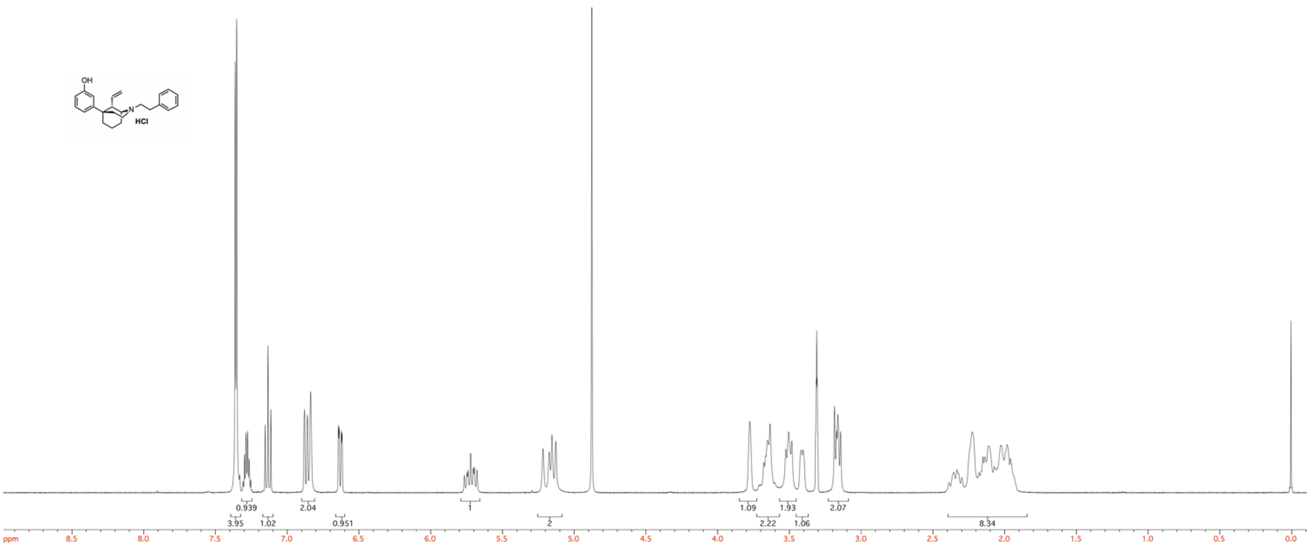
OH
N
HCl
0.939
2.04
1
1.09
1.93
2.07
3.95
1.02
0.951
2
2.22
1.06
8.34
ppm
8.5
8.0
7.5
7.0
6.5
6.0
5.5
5.0
4.5
4.0
3.5
3.0
2.5
2.0
1.5
1.0
0.5
0.0

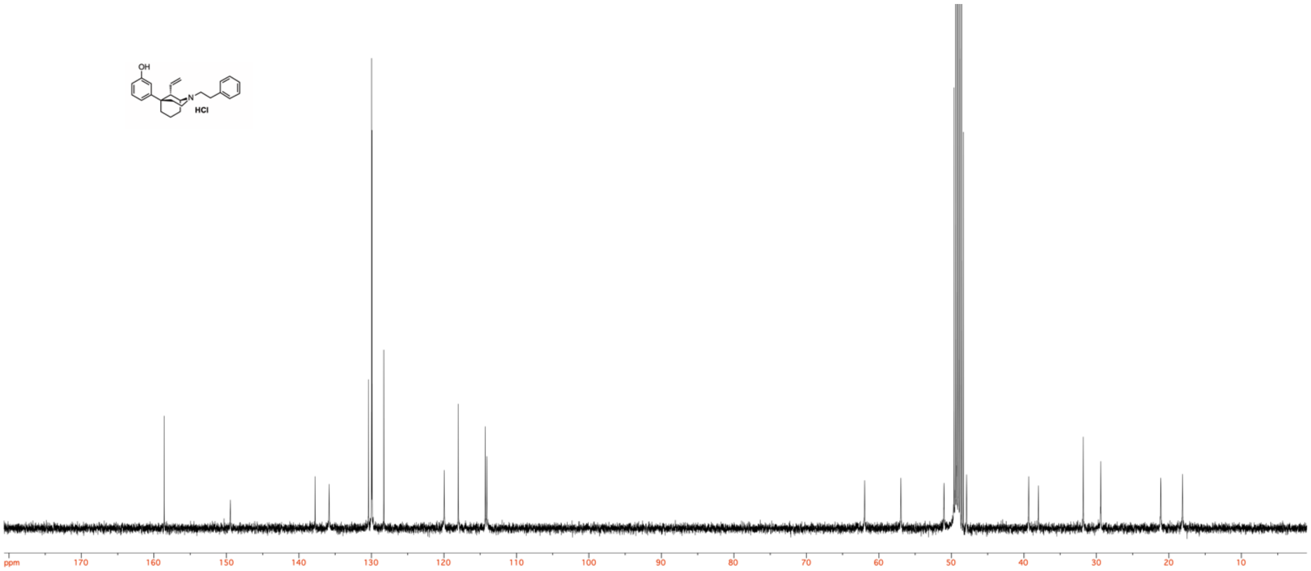
OH
N
HCl
ppm
170
160
150
140
130
120
110
100
90
80
70
60
50
40
30
20
10

Compound **15**

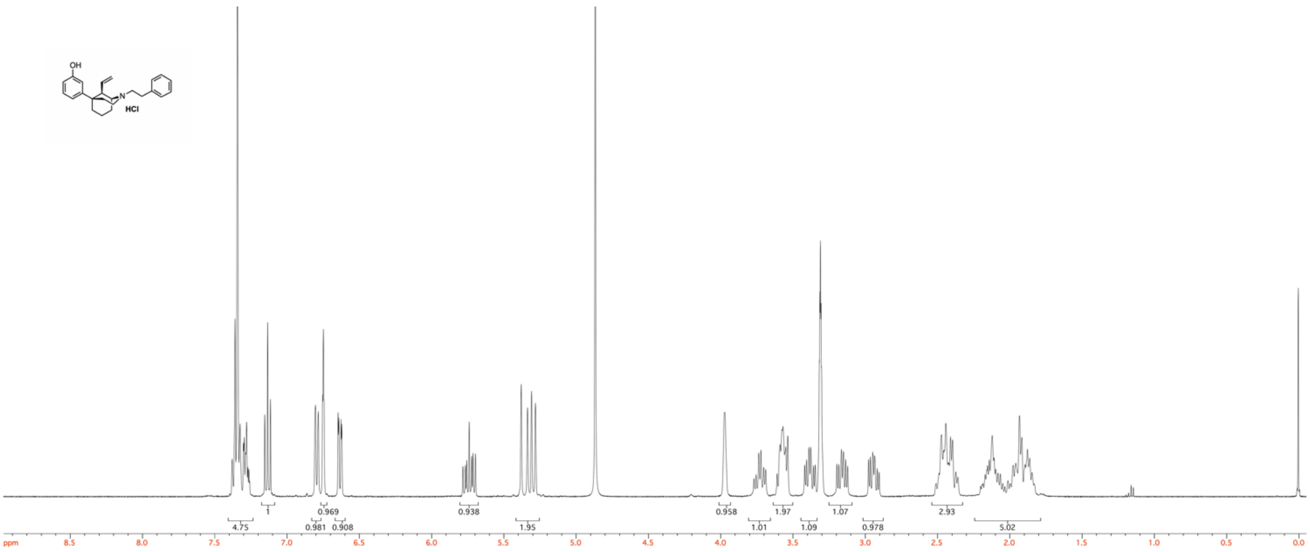

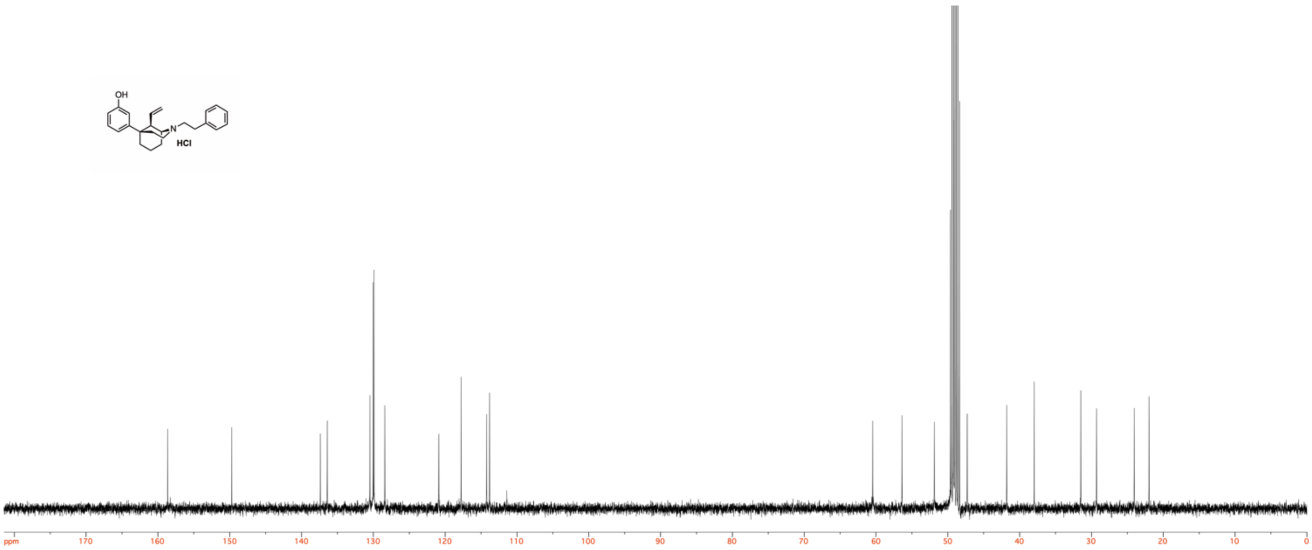

Compound **17**

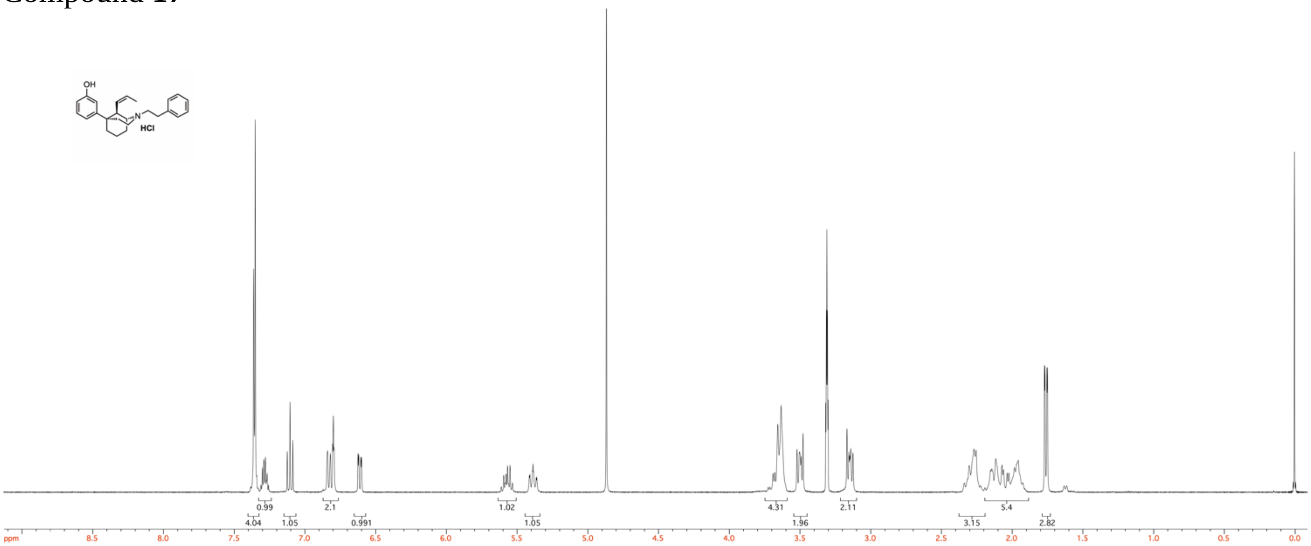
OH
N
HCl
0.99
4.04
1.05
2.1
0.991
1.02
1.05
4.31
1.96
2.11
3.15
5.4
2.82
ppm
8.5
8.0
7.5
7.0
6.5
6.0
5.5
5.0
4.5
4.0
3.5
3.0
2.5
2.0
1.5
1.0
0.5
0.0

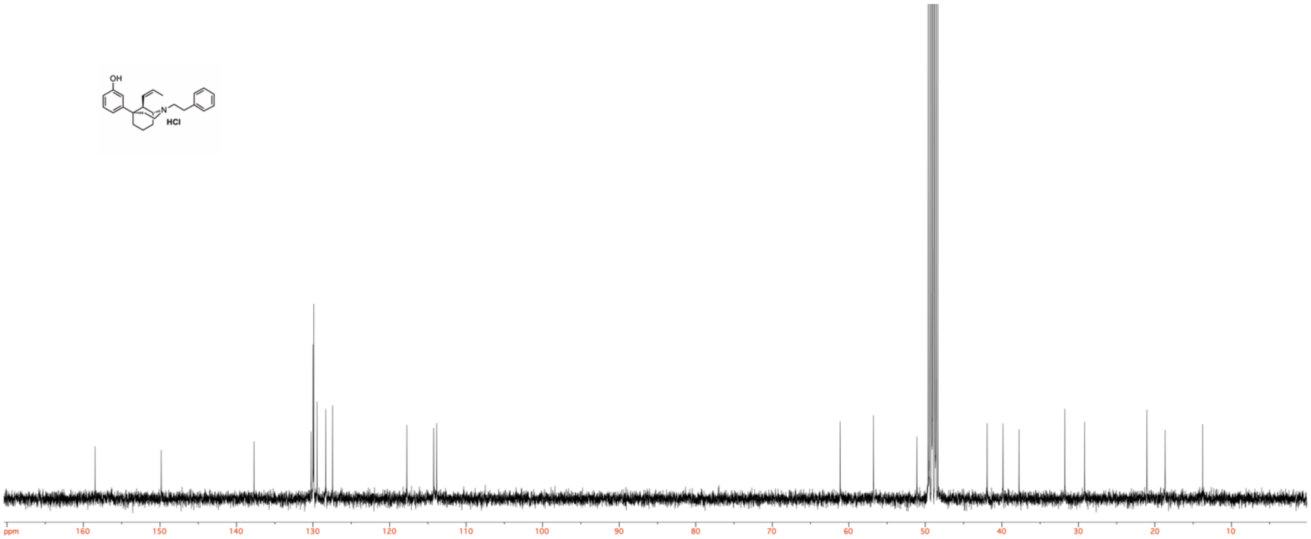
OH
N
HCl
ppm
160
150
140
130
120
110
100
90
80
70
60
50
40
30
20
10

Compound **18**

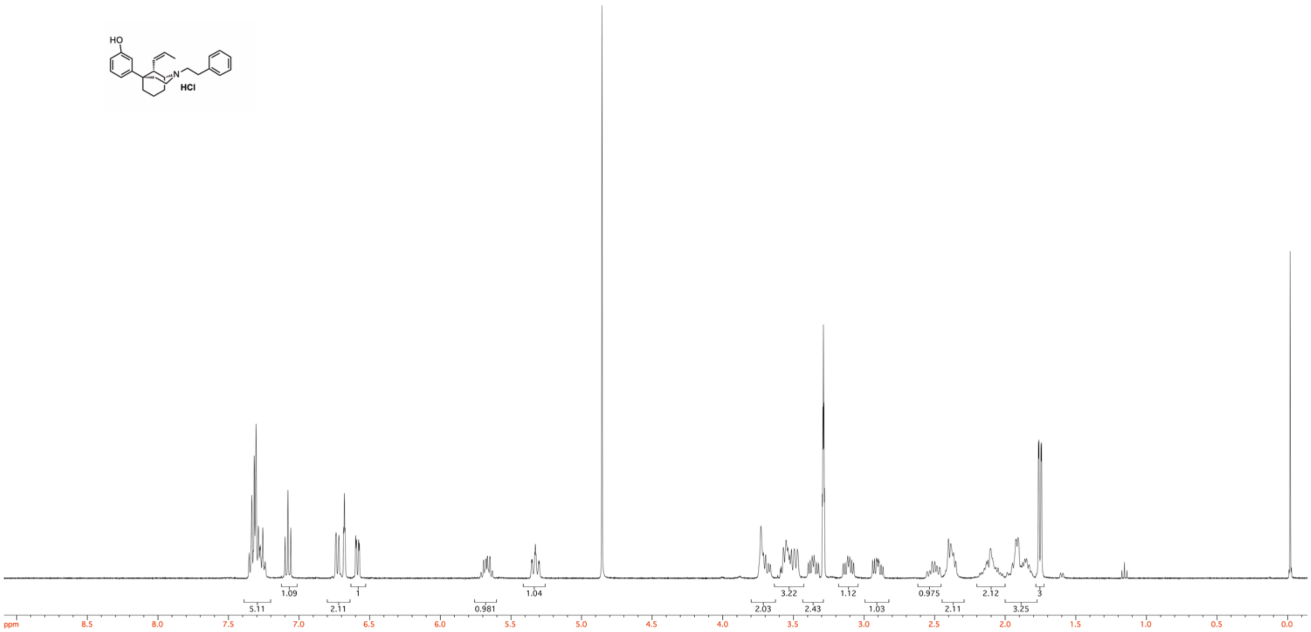

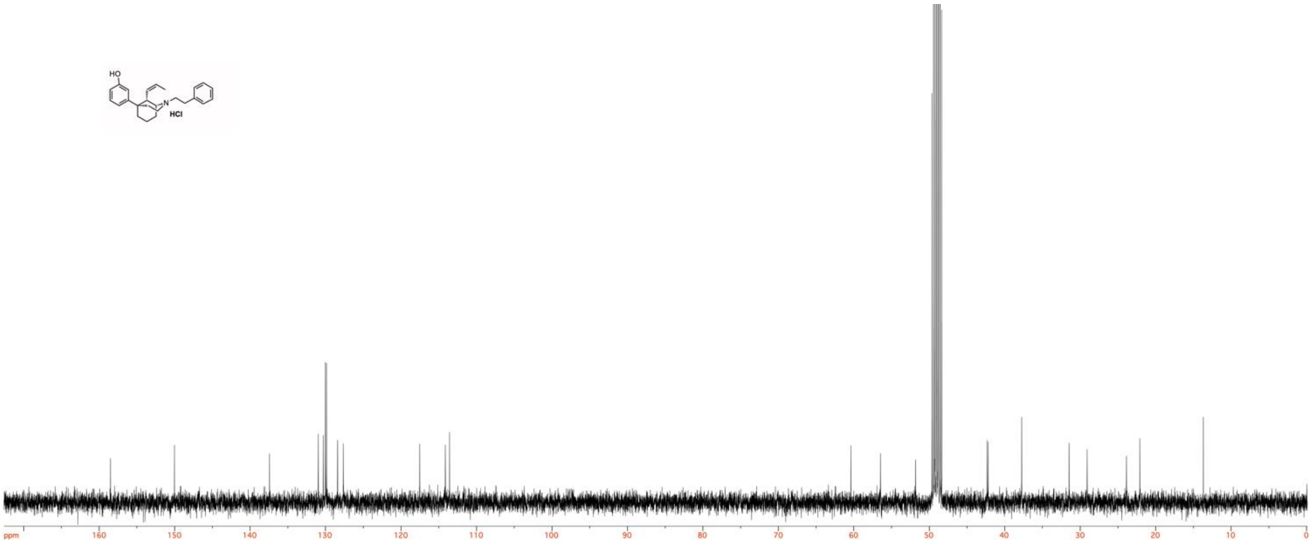

Compound **20**

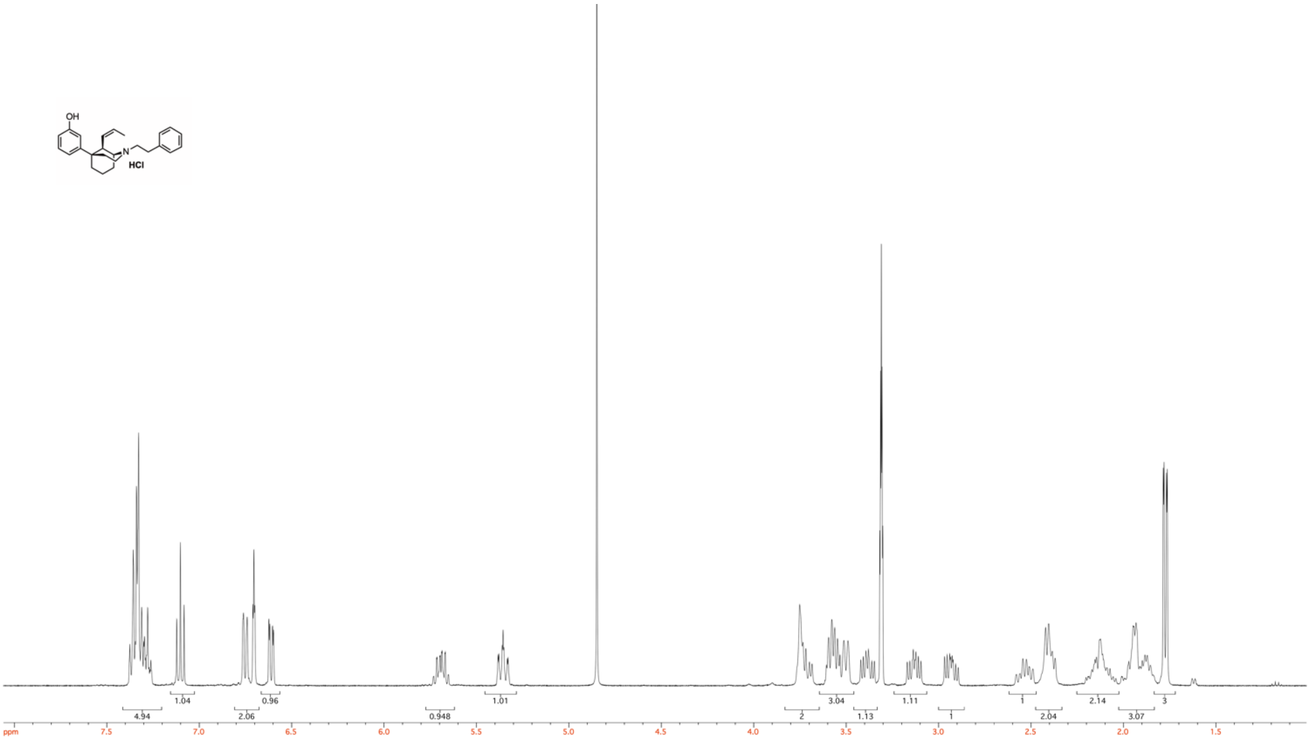
OH
N
HCl
4.94
1.04
2.06
0.96
0.948
1.01
2
3.04
1.13
1.11
1
1
2.04
2.14
3.07
3
ppm
7.5
7.0
6.5
6.0
5.5
5.0
4.5
4.0
3.5
3.0
2.5
2.0
1.5

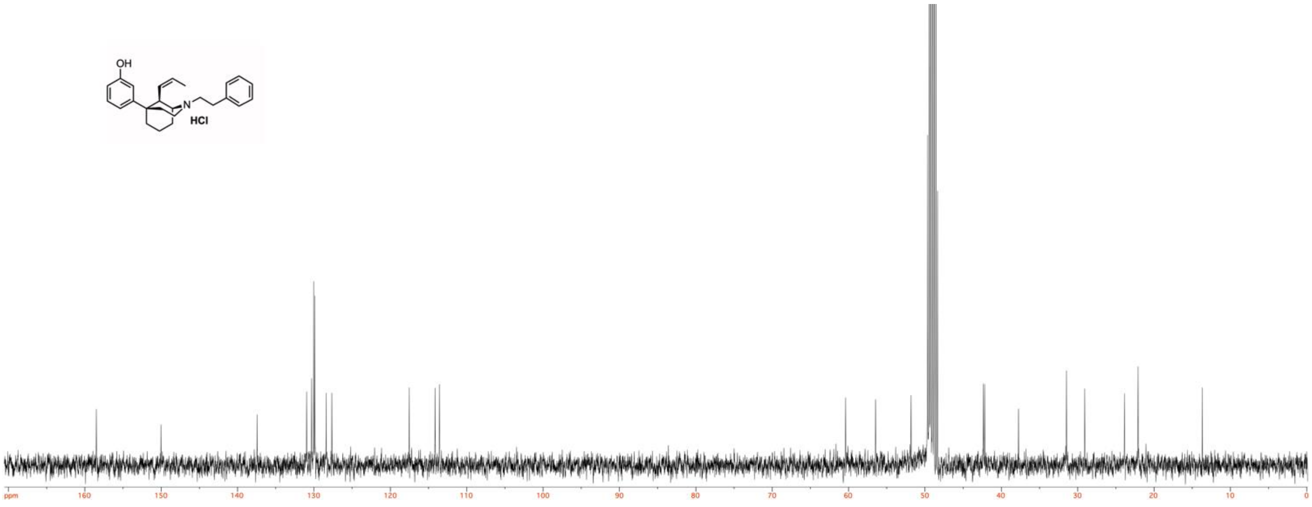
OH
N
HCl
ppm
160
150
140
130
120
110
100
90
80
70
60
50
40
30
20
10
0

Compound **21**

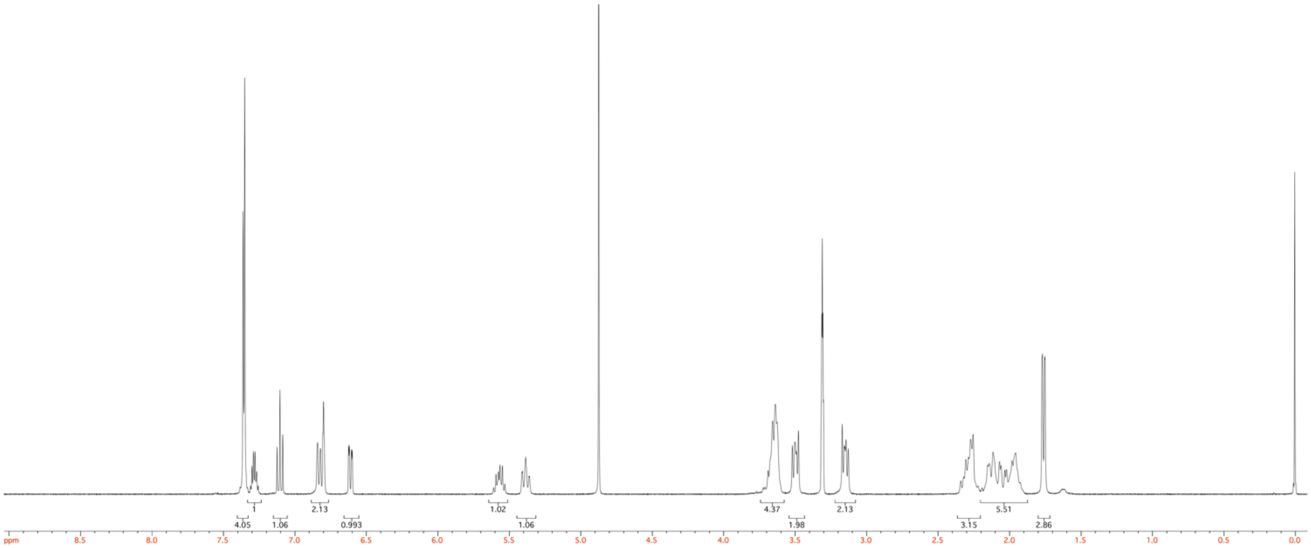

4.05
1
1.06
2.13
0.993
1.02
1.06
4.37
1.98
2.13
3.15
5.51
2.86
ppm 8.5 8.0 7.5 7.0 6.5 6.0 5.5 5.0 4.5 4.0 3.5 3.0 2.5 2.0 1.5 1.0 0.5 0.0

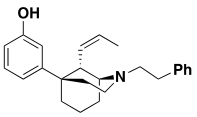

OH
N
Ph

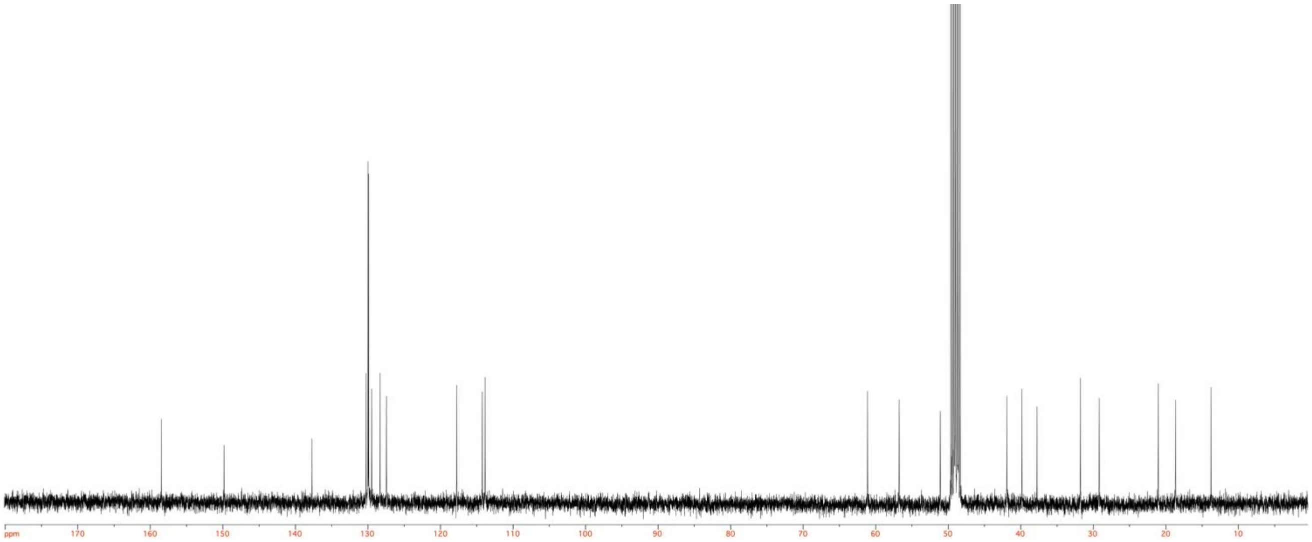

ppm 170 160 150 140 130 120 110 100 90 80 70 60 50 40 30 20 10

Compound **23**

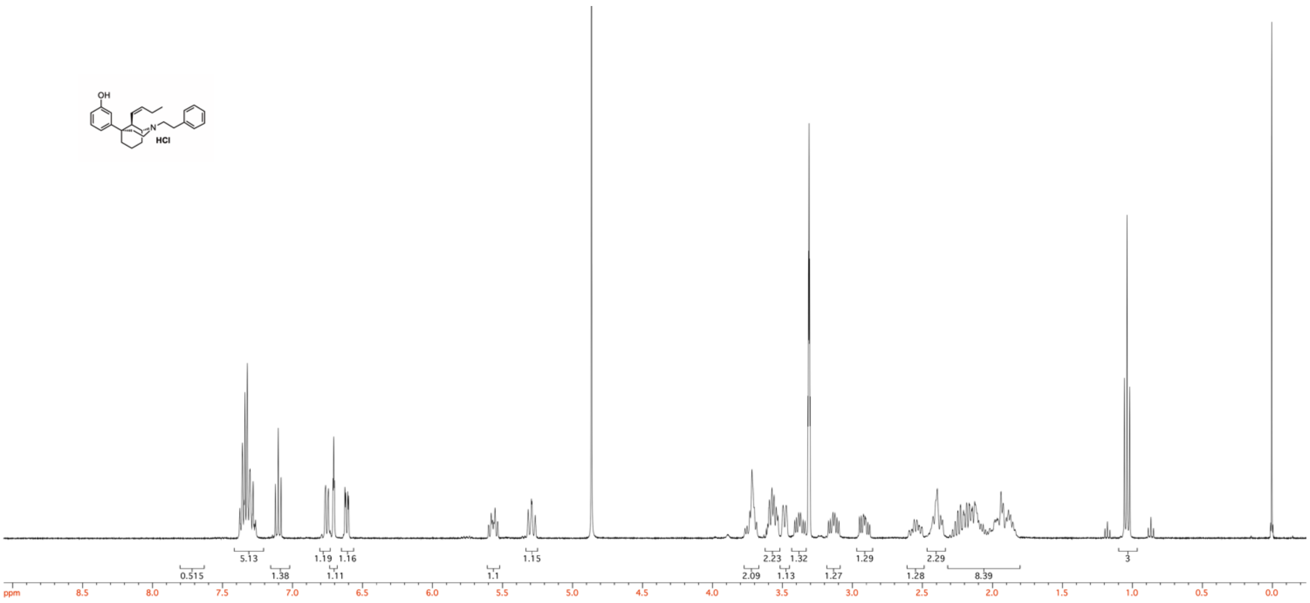

OH
N
HCl
5.13
1.19
1.16
0.515
1.38
1.11
1.15
1.1
2.23
1.32
2.09
1.13
1.29
1.27
2.29
1.28
8.39
3
ppm
8.5
8.0
7.5
7.0
6.5
6.0
5.5
5.0
4.5
4.0
3.5
3.0
2.5
2.0
1.5
1.0
0.5
0.0

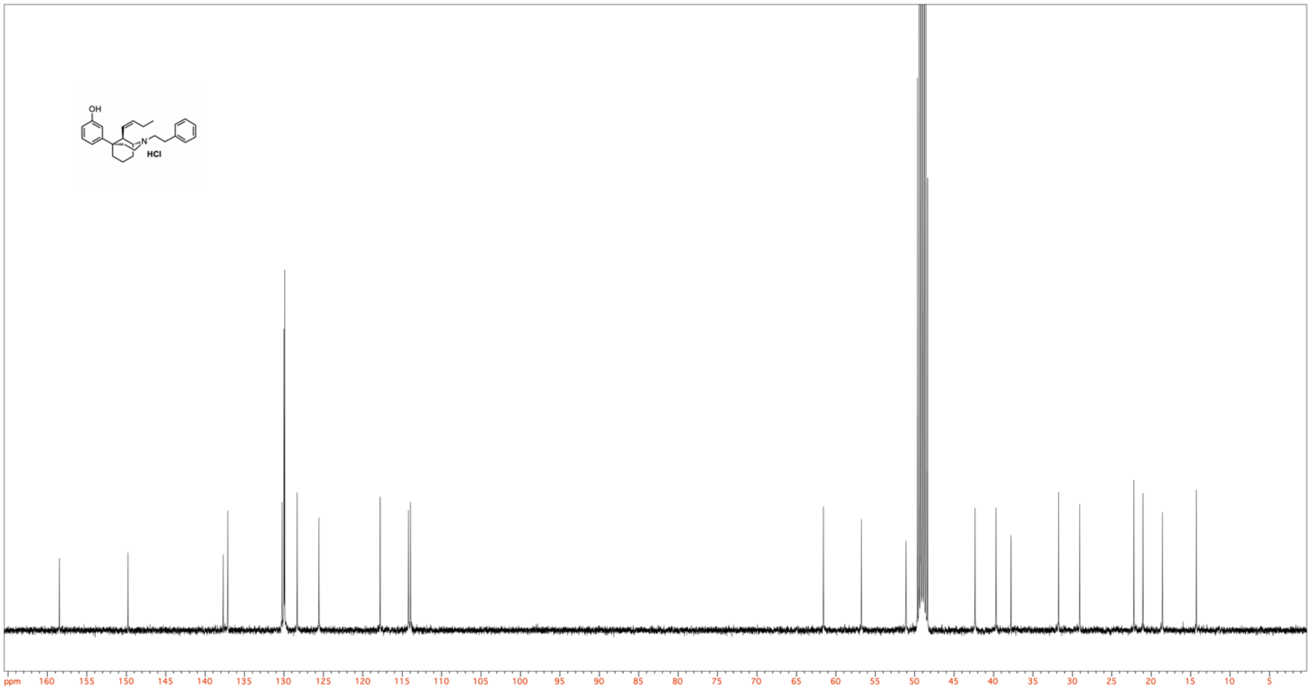

Compound **24**

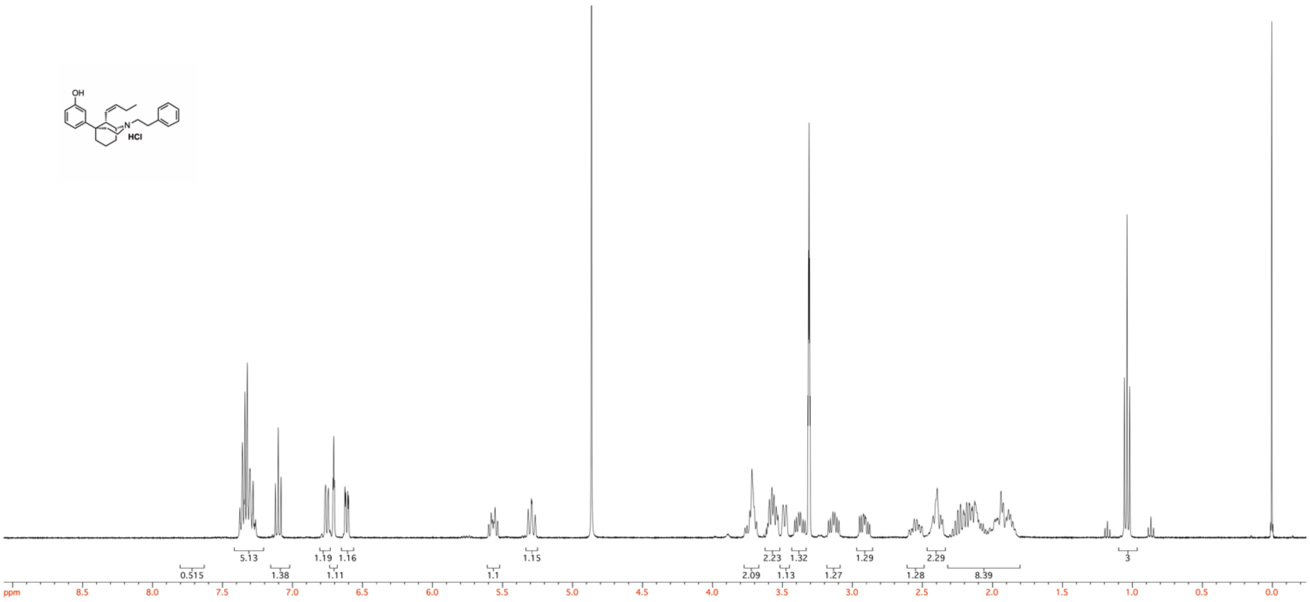

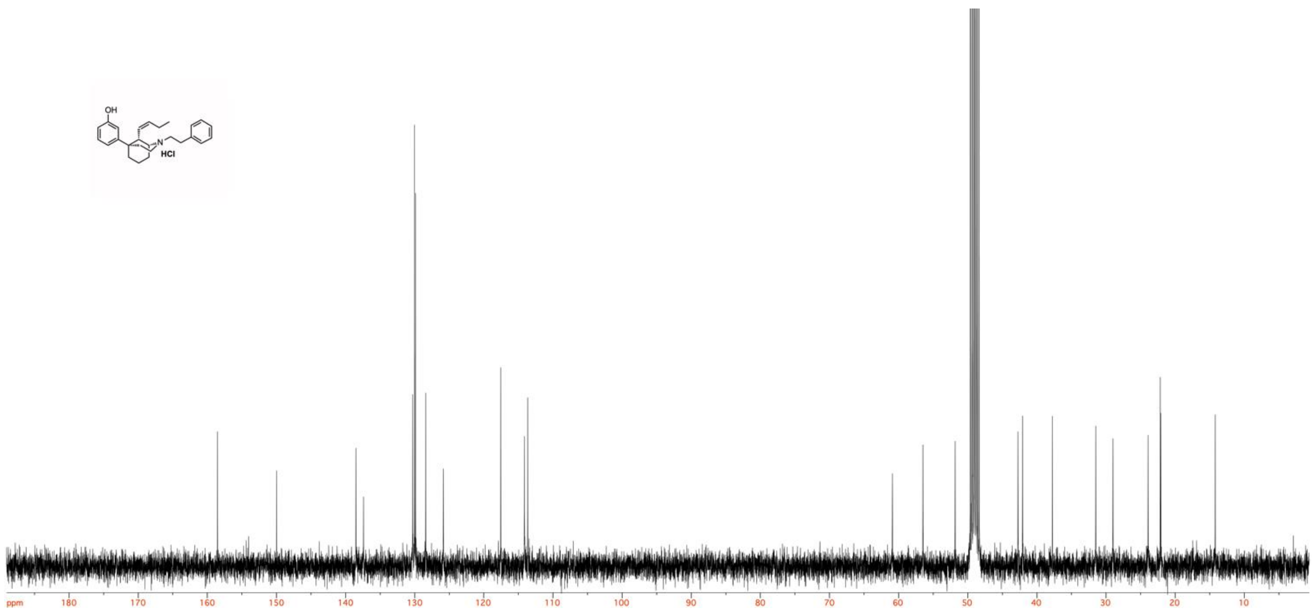
OH
N
HCl
ppm
180
170
160
150
140
130
120
110
100
90
80
70
60
50
40
30
20
10

Compound **26**

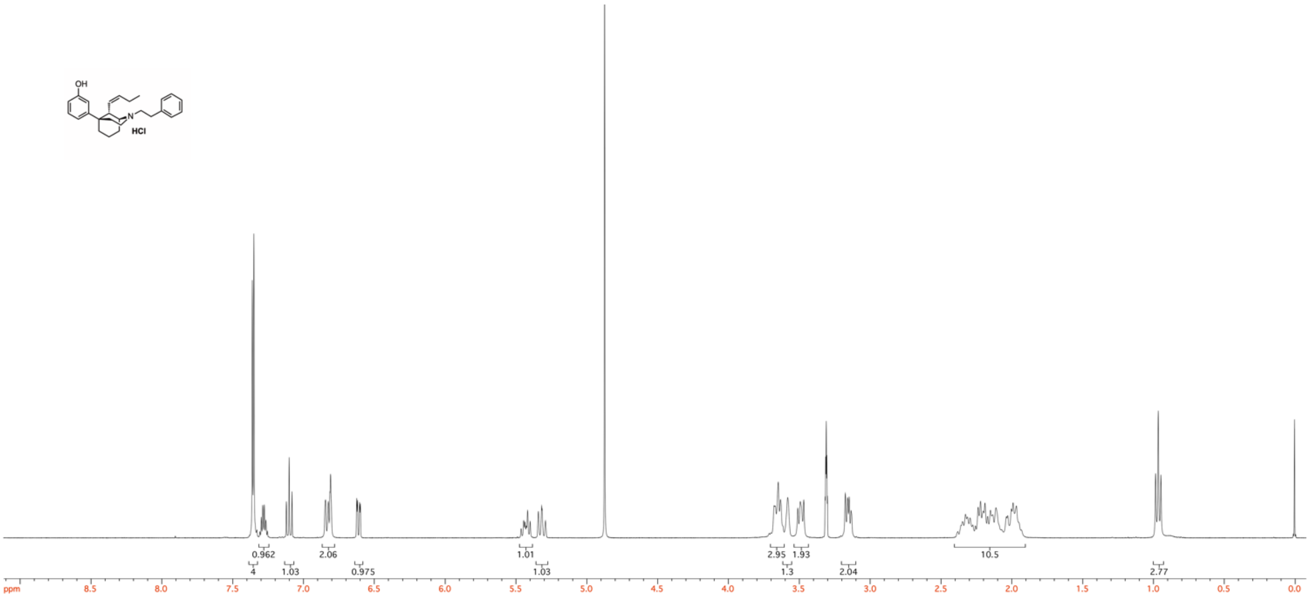
OH
N
HCl
0.962
4
1.03
2.06
0.975
1.01
1.03
2.95
1.3
1.93
2.04
10.5
2.77
ppm 8.5 8.0 7.5 7.0 6.5 6.0 5.5 5.0 4.5 4.0 3.5 3.0 2.5 2.0 1.5 1.0 0.5 0.0

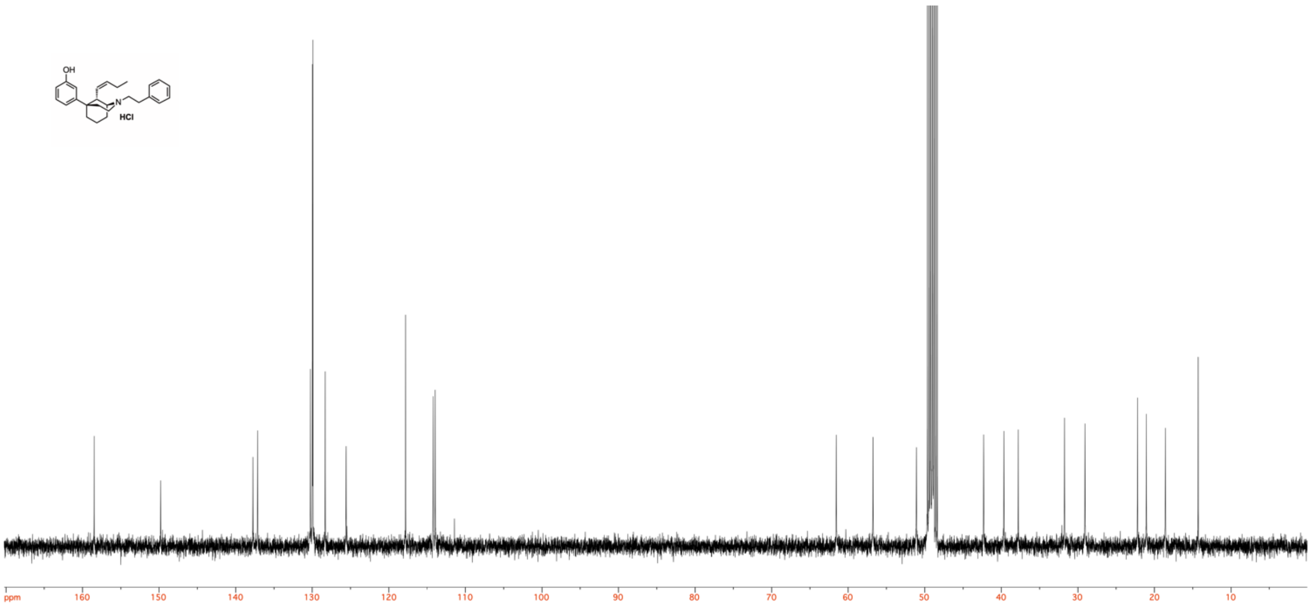
OH
N
HCl
ppm 160 150 140 130 120 110 100 90 80 70 60 50 40 30 20 10

Compound **27**

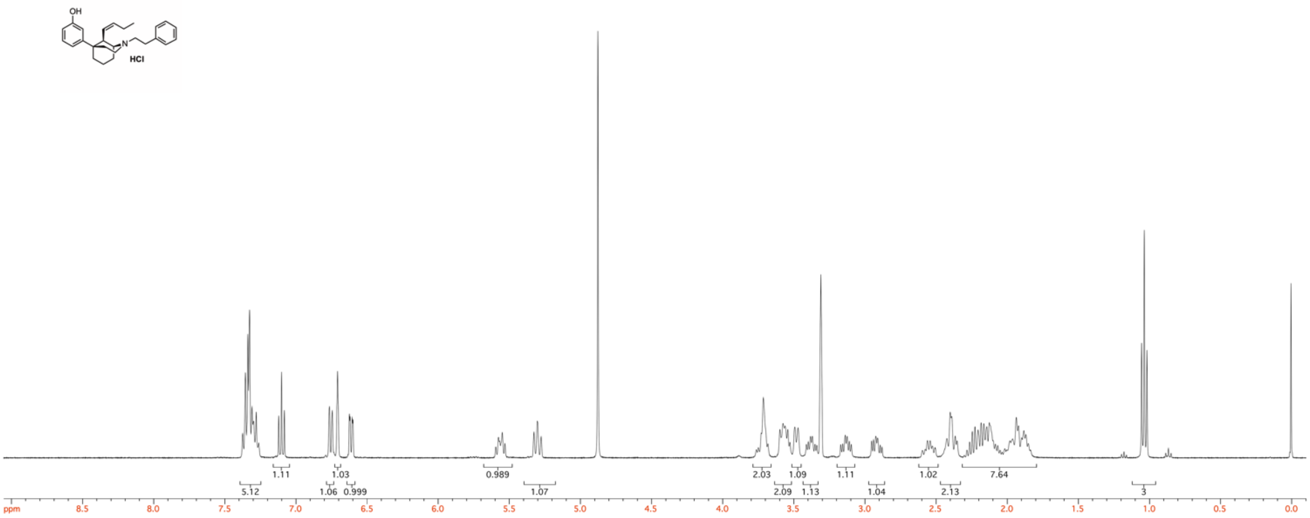
OH
N
HCl
5.12
1.11
1.06
1.03
0.999
0.989
1.07
2.03
2.09
1.09
1.13
1.11
1.04
1.02
2.13
7.64
3
ppm 8.5 8.0 7.5 7.0 6.5 6.0 5.5 5.0 4.5 4.0 3.5 3.0 2.5 2.0 1.5 1.0 0.5 0.0

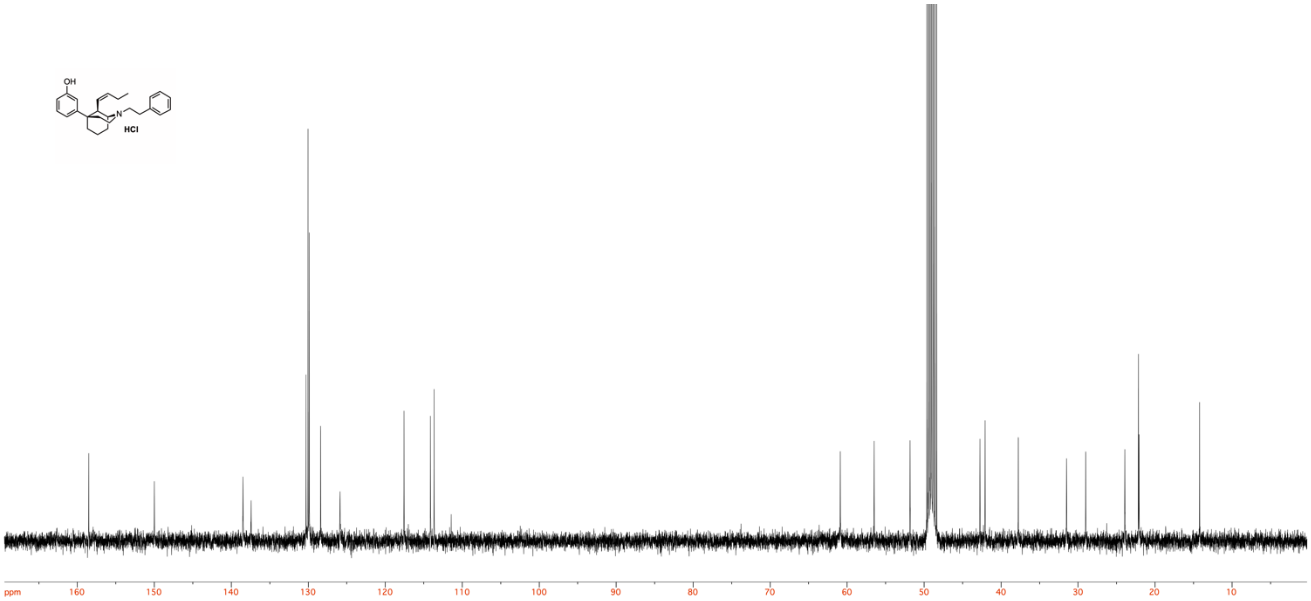
OH
N
HCl
ppm 160 150 140 130 120 110 100 90 80 70 60 50 40 30 20 10

Table S1. Crystal data and structure refinement for Compound **8**

Identification code	knih132	
Empirical formula	$C_{24}H_{30}ClNO$	
Formula weight	383.94	
Temperature	293(2) K	
Wavelength	1.54178 Å	
Crystal system	Monoclinic	
Space group	$P2_1$	
Unit cell dimensions	a = 9.0396(2) Å	〈= 90°.
	b = 9.0396(2) Å	®= 9.0396(2) °.
	c = 9.0396(2) Å	© = 90°.
Volume	9.0396(2) $Å^3$	
Z	2	
Density (20°C)	1.214 Mg/m^3	
Absorption coefficient	1.694 mm^{-1}	
F(000)	412	
Crystal size	0.205 x 0.168 x 0.060 mm^3	
Theta range for data collection	4.713 to 74.487°.	
Index ranges	-11<=h<=11, -13<=k<=14, -11<=l<=11	
Reflections collected	21280	
Independent reflections	4103 [R_{int} = 0.0197]	
Completeness to theta = 67.679°	99.4 %	
Absorption correction	Semi-empirical from equivalents	
Max. and min. transmission	0.7538 and 0.6809	
Refinement method	Full-matrix least-squares on F^2	
Data / restraints / parameters	4103 / 1 / 251	
Goodness-of-fit on F^2	1.050	
Final R indices [I>2sigma(I)]	R_1 = 0.0277, wR_2 = 0.0764	
R indices (all data)	R_1 = 0.0281, wR_2 = 0.0768	
Absolute structure parameter	0.038(3)	
Largest diff. peak and hole	0.295 and -0.240 e.$Å^{-3}$	

Table S2. Atomic coordinates (x 10^4) and equivalent isotropic displacement parameters (Å^{2}x 10^3) for DC-01-00-76.1. U(eq) is defined as one third of the trace of the orthogonalized U^{ij} tensor.

	x	y	z	U(eq)
C(1)	4831(2)	5236(2)	7119(2)	34(1)
N(2)	4632(2)	4657(1)	8521(2)	34(1)
C(3)	3367(2)	5084(2)	9355(2)	38(1)
C(4)	1964(2)	5265(2)	8441(2)	39(1)
C(5)	2151(2)	5792(1)	6964(2)	32(1)
C(6)	2635(2)	6981(2)	7072(2)	41(1)
C(7)	4158(2)	7175(2)	7793(2)	45(1)
C(8)	5340(2)	6402(2)	7287(2)	43(1)
C(9)	3372(2)	5128(1)	6234(2)	33(1)
C(10)	3624(2)	5434(2)	4706(2)	45(1)
C(11)	3706(3)	4742(3)	3687(3)	73(1)
C(12)	6004(2)	4597(2)	9471(2)	40(1)
C(13)	7325(2)	4157(2)	8697(2)	49(1)
C(14)	8596(2)	3826(2)	9687(2)	44(1)
C(15)	9432(3)	2924(2)	9368(3)	59(1)
C(16)	10656(3)	2637(2)	10215(4)	70(1)
C(17)	11054(2)	3233(3)	11387(3)	66(1)
C(18)	10240(3)	4130(3)	11731(3)	59(1)
C(19)	9014(2)	4420(2)	10885(2)	50(1)
C(20)	671(2)	5678(2)	6114(2)	34(1)
C(21)	61(2)	4658(2)	5865(2)	41(1)
C(22)	-1267(2)	4548(2)	5094(2)	46(1)
C(23)	-2017(2)	5433(2)	4532(2)	43(1)
O(24)	-2126(2)	7361(2)	4325(3)	79(1)
C(24)	-1431(2)	6444(2)	4786(2)	47(1)
C(25)	-107(2)	6563(2)	5573(2)	45(1)
Cl(26)	5708(1)	7177(1)	1751(1)	61(1)

Table S3. Bond lengths [Å] and angles [°] for DC-01-00-76.1.

C(1)-N(2)	1.515(2)	C(1)-C(8)	1.522(3)
C(1)-C(9)	1.539(2)	C(1)-H(1)	0.9800
N(2)-C(12)	1.503(2)	N(2)-C(3)	1.505(2)
N(2)-H(2)	0.93(3)	C(3)-C(4)	1.523(2)
C(3)-H(3A)	0.9700	C(3)-H(3B)	0.9700
C(4)-C(5)	1.548(2)	C(4)-H(4A)	0.9700
C(4)-H(4B)	0.9700	C(5)-C(6)	1.538(2)
C(5)-C(20)	1.540(2)	C(5)-C(9)	1.554(2)
C(6)-C(7)	1.532(2)	C(6)-H(6A)	0.9700
C(6)-H(6B)	0.9700	C(7)-C(8)	1.523(3)
C(7)-H(7A)	0.9700	C(7)-H(7B)	0.9700
C(8)-H(8A)	0.9700	C(8)-H(8B)	0.9700
C(9)-C(10)	1.509(2)	C(9)-H(9A)	0.9800
C(10)-C(11)	1.288(4)	C(10)-H(10)	0.9300
C(11)-H(11A)	0.9300	C(11)-H(11B)	0.9300
C(12)-C(13)	1.520(3)	C(12)-H(12A)	0.9700
C(12)-H(12B)	0.9700	C(13)-C(14)	1.509(3)
C(13)-H(13A)	0.9700	C(13)-H(13B)	0.9700
C(14)-C(19)	1.385(3)	C(14)-C(15)	1.387(3)
C(15)-C(16)	1.386(4)	C(15)-H(15)	0.9300
C(16)-C(17)	1.363(5)	C(16)-H(16)	0.9300
C(17)-C(18)	1.377(4)	C(17)-H(17)	0.9300
C(18)-C(19)	1.388(3)	C(18)-H(18)	0.9300
C(19)-H(19)	0.9300	C(20)-C(25)	1.388(3)
C(20)-C(21)	1.395(3)	C(21)-C(22)	1.386(3)
C(21)-H(21)	0.9300	C(22)-C(23)	1.383(3)
C(22)-H(22)	0.9300	C(23)-C(24)	1.377(3)
C(23)-H(23)	0.9300	O(24)-C(24)	1.361(3)
O(24)-H(24)	0.88(4)	C(24)-C(25)	1.393(3)
C(25)-H(25)	0.9300		
N(2)-C(1)-C(8)	113.82(15)	N(2)-C(1)-C(9)	107.59(13)
C(8)-C(1)-C(9)	112.82(15)	N(2)-C(1)-H(1)	107.4
C(8)-C(1)-H(1)	107.4	C(9)-C(1)-H(1)	107.4
C(12)-N(2)-C(3)	109.56(14)	C(12)-N(2)-C(1)	114.72(13)
C(3)-N(2)-C(1)	113.64(14)	C(12)-N(2)-H(2)	104.7(16)
C(3)-N(2)-H(2)	109.9(16)	C(1)-N(2)-H(2)	103.7(16)
N(2)-C(3)-C(4)	113.10(14)	N(2)-C(3)-H(3A)	109.0
C(4)-C(3)-H(3A)	109.0	N(2)-C(3)-H(3B)	109.0
C(4)-C(3)-H(3B)	109.0	H(3A)-C(3)-H(3B)	107.8
C(3)-C(4)-C(5)	116.86(14)	C(3)-C(4)-H(4A)	108.1
C(5)-C(4)-H(4A)	108.1	C(3)-C(4)-H(4B)	108.1
C(5)-C(4)-H(4B)	108.1	H(4A)-C(4)-H(4B)	107.3
C(6)-C(5)-C(20)	111.20(14)	C(6)-C(5)-C(4)	112.59(15)

Table S3. (continued).

C(20)-C(5)-C(4)	107.78(14)	C(6)-C(5)-C(9)	109.31(14)
C(20)-C(5)-C(9)	109.93(14)	C(4)-C(5)-C(9)	105.88(14)
C(7)-C(6)-C(5)	115.36(16)	C(7)-C(6)-H(6A)	108.4
C(5)-C(6)-H(6A)	108.4	C(7)-C(6)-H(6B)	108.4
C(5)-C(6)-H(6B)	108.4	H(6A)-C(6)-H(6B)	107.5
C(8)-C(7)-C(6)	113.16(16)	C(8)-C(7)-H(7A)	108.9
C(6)-C(7)-H(7A)	108.9	C(8)-C(7)-H(7B)	108.9
C(6)-C(7)-H(7B)	108.9	H(7A)-C(7)-H(7B)	107.8
C(1)-C(8)-C(7)	114.57(15)	C(1)-C(8)-H(8A)	108.6
C(7)-C(8)-H(8A)	108.6	C(1)-C(8)-H(8B)	108.6
C(7)-C(8)-H(8B)	108.6	H(8A)-C(8)-H(8B)	107.6
C(10)-C(9)-C(1)	109.63(14)	C(10)-C(9)-C(5)	114.83(15)
C(1)-C(9)-C(5)	108.96(14)	C(10)-C(9)-H(9A)	107.7
C(1)-C(9)-H(9A)	107.7	C(5)-C(9)-H(9A)	107.7
C(11)-C(10)-C(9)	123.7(2)	C(11)-C(10)-H(10)	118.2
C(9)-C(10)-H(10)	118.2	C(10)-C(11)-H(11A)	120.0
C(10)-C(11)-H(11B)	120.0	H(11A)-C(11)-H(11B)	120.0
N(2)-C(12)-C(13)	112.29(15)	N(2)-C(12)-H(12A)	109.1
C(13)-C(12)-H(12A)	109.1	N(2)-C(12)-H(12B)	109.1
C(13)-C(12)-H(12B)	109.1	H(12A)-C(12)-H(12B)	107.9
C(14)-C(13)-C(12)	113.36(16)	C(14)-C(13)-H(13A)	108.9
C(12)-C(13)-H(13A)	108.9	C(14)-C(13)-H(13B)	108.9
C(12)-C(13)-H(13B)	108.9	H(13A)-C(13)-H(13B)	107.7
C(19)-C(14)-C(15)	117.9(2)	C(19)-C(14)-C(13)	122.4(2)
C(15)-C(14)-C(13)	119.6(2)	C(16)-C(15)-C(14)	120.8(3)
C(16)-C(15)-H(15)	119.6	C(14)-C(15)-H(15)	119.6
C(17)-C(16)-C(15)	120.4(3)	C(17)-C(16)-H(16)	119.8
C(15)-C(16)-H(16)	119.8	C(16)-C(17)-C(18)	119.9(2)
C(16)-C(17)-H(17)	120.1	C(18)-C(17)-H(17)	120.1
C(17)-C(18)-C(19)	119.8(3)	C(17)-C(18)-H(18)	120.1
C(19)-C(18)-H(18)	120.1	C(14)-C(19)-C(18)	121.1(2)
C(14)-C(19)-H(19)	119.4	C(18)-C(19)-H(19)	119.4
C(25)-C(20)-C(21)	117.51(17)	C(25)-C(20)-C(5)	122.45(16)
C(21)-C(20)-C(5)	120.04(16)	C(22)-C(21)-C(20)	120.40(19)
C(22)-C(21)-H(21)	119.8	C(20)-C(21)-H(21)	119.8
C(23)-C(22)-C(21)	121.67(19)	C(23)-C(22)-H(22)	119.2
C(21)-C(22)-H(22)	119.2	C(24)-C(23)-C(22)	118.31(18)
C(24)-C(23)-H(23)	120.8	C(22)-C(23)-H(23)	120.8
C(24)-O(24)-H(24)	109(4)	O(24)-C(24)-C(23)	122.24(18)
O(24)-C(24)-C(25)	117.30(19)	C(23)-C(24)-C(25)	120.43(19)
C(20)-C(25)-C(24)	121.65(19)	C(20)-C(25)-H(25)	119.2
C(24)-C(25)-H(25)	119.2		

Table S4. Anisotropic displacement parameters ($Å^2$x 10^3) for DC-01-00-76.1. The anisotropic displacement factor exponent takes the form: $-2\square^2[h^2a^{*2}U^{11} + ... + 2 h k a^* b^* U^{12}]$

	U^{11}	U^{22}	U^{33}	U^{23}	U^{13}	U^{12}
C(1)	31(1)	40(1)	31(1)	4(1)	2(1)	2(1)
N(2)	34(1)	35(1)	33(1)	4(1)	-2(1)	0(1)
C(3)	37(1)	46(1)	31(1)	4(1)	4(1)	1(1)
C(4)	33(1)	46(1)	38(1)	3(1)	4(1)	1(1)
C(5)	32(1)	30(1)	35(1)	0(1)	-1(1)	1(1)
C(6)	41(1)	31(1)	49(1)	-3(1)	-6(1)	0(1)
C(7)	48(1)	33(1)	53(1)	1(1)	-10(1)	-10(1)
C(8)	35(1)	45(1)	50(1)	11(1)	-5(1)	-10(1)
C(9)	33(1)	32(1)	33(1)	1(1)	-1(1)	0(1)
C(10)	41(1)	59(1)	34(1)	6(1)	0(1)	4(1)
C(11)	85(2)	95(2)	38(1)	-11(1)	7(1)	-3(2)
C(12)	37(1)	47(1)	35(1)	4(1)	-6(1)	2(1)
C(13)	40(1)	64(1)	42(1)	1(1)	-1(1)	5(1)
C(14)	35(1)	49(1)	49(1)	11(1)	2(1)	0(1)
C(15)	50(1)	53(1)	75(2)	4(1)	4(1)	1(1)
C(16)	47(1)	56(2)	105(2)	15(2)	5(1)	13(1)
C(17)	37(1)	83(2)	79(2)	30(2)	-5(1)	3(1)
C(18)	42(1)	84(2)	52(1)	15(1)	-3(1)	-8(1)
C(19)	40(1)	61(2)	48(1)	8(1)	-2(1)	1(1)
C(20)	32(1)	32(1)	38(1)	-2(1)	1(1)	0(1)
C(21)	39(1)	31(1)	53(1)	-2(1)	-2(1)	2(1)
C(22)	41(1)	36(1)	62(1)	-10(1)	-3(1)	-6(1)
C(23)	32(1)	46(1)	52(1)	-10(1)	-6(1)	-1(1)
O(24)	72(1)	42(1)	118(2)	8(1)	-52(1)	3(1)
C(24)	41(1)	38(1)	60(1)	1(1)	-11(1)	2(1)
C(25)	43(1)	32(1)	60(1)	0(1)	-13(1)	-2(1)
Cl(26)	70(1)	42(1)	69(1)	5(1)	-23(1)	0(1)

Table S5. Hydrogen coordinates (x 10^4) and isotropic displacement parameters ($Å^2$ x 10^3) for DC-01-00-76.1.

	x	y	z	U(eq)
H(1)	5595	4848	6609	41
H(2)	4440(30)	3950(30)	8250(30)	52(7)
H(3A)	3662	5762	9798	45
H(3B)	3156	4577	10109	45
H(4A)	1301	5715	8975	47
H(4B)	1481	4572	8296	47
H(6A)	2637	7282	6118	49
H(6B)	1902	7374	7597	49
H(7A)	4465	7910	7604	54
H(7B)	4078	7098	8815	54
H(8A)	5680	6654	6377	52
H(8B)	6177	6423	7961	52
H(9A)	3076	4367	6249	39
H(10)	3727	6162	4487	54
H(11A)	3608	4009	3875	87
H(11B)	3864	4976	2763	87
H(12A)	5813	4136	10280	48
H(12B)	6239	5313	9832	48
H(13A)	7009	3536	8133	59
H(13B)	7667	4704	8047	59
H(15)	9167	2507	8576	71
H(16)	11211	2034	9981	84
H(17)	11873	3034	11954	80
H(18)	10511	4541	12527	71
H(19)	8463	5023	11126	60
H(21)	548	4048	6218	50
H(22)	-1664	3862	4952	55
H(23)	-2894	5347	3996	52
H(24)	-2870(50)	7190(50)	3730(50)	118
H(25)	267	7253	5741	54

Table S6. Torsion angles [°] for DC-01-00-76.1.

C(8)-C(1)-N(2)-C(12)	-59.4(2)	C(9)-C(1)-N(2)-C(12)	174.85(15)
C(8)-C(1)-N(2)-C(3)	67.78(19)	C(9)-C(1)-N(2)-C(3)	-58.00(19)
C(12)-N(2)-C(3)-C(4)	174.93(16)	C(1)-N(2)-C(3)-C(4)	45.1(2)
N(2)-C(3)-C(4)-C(5)	-42.6(2)	C(3)-C(4)-C(5)-C(6)	-68.7(2)
C(3)-C(4)-C(5)-C(20)	168.27(16)	C(3)-C(4)-C(5)-C(9)	50.7(2)
C(20)-C(5)-C(6)-C(7)	-175.21(15)	C(4)-C(5)-C(6)-C(7)	63.7(2)
C(9)-C(5)-C(6)-C(7)	-53.6(2)	C(5)-C(6)-C(7)-C(8)	44.8(2)
N(2)-C(1)-C(8)-C(7)	-72.7(2)	C(9)-C(1)-C(8)-C(7)	50.3(2)
C(6)-C(7)-C(8)-C(1)	-42.0(2)	N(2)-C(1)-C(9)-C(10)	-165.60(16)
C(8)-C(1)-C(9)-C(10)	68.0(2)	N(2)-C(1)-C(9)-C(5)	67.96(18)
C(8)-C(1)-C(9)-C(5)	-58.42(18)	C(6)-C(5)-C(9)-C(10)	-64.6(2)
C(20)-C(5)-C(9)-C(10)	57.8(2)	C(4)-C(5)-C(9)-C(10)	173.91(16)
C(6)-C(5)-C(9)-C(1)	58.83(18)	C(20)-C(5)-C(9)-C(1)	-178.85(14)
C(4)-C(5)-C(9)-C(1)	-62.69(18)	C(1)-C(9)-C(10)-C(11)	104.9(3)
C(5)-C(9)-C(10)-C(11)	-132.1(2)	C(3)-N(2)-C(12)-C(13)	179.31(17)
C(1)-N(2)-C(12)-C(13)	-51.5(2)	N(2)-C(12)-C(13)-C(14)	-165.59(18)
C(12)-C(13)-C(14)-C(19)	-39.9(3)	C(12)-C(13)-C(14)-C(15)	143.2(2)
C(19)-C(14)-C(15)-C(16)	-0.8(3)	C(13)-C(14)-C(15)-C(16)	176.3(2)
C(14)-C(15)-C(16)-C(17)	0.6(4)	C(15)-C(16)-C(17)-C(18)	-0.4(4)
C(16)-C(17)-C(18)-C(19)	0.3(4)	C(15)-C(14)-C(19)-C(18)	0.7(3)
C(13)-C(14)-C(19)-C(18)	-176.3(2)	C(17)-C(18)-C(19)-C(14)	-0.5(3)
C(6)-C(5)-C(20)-C(25)	-1.8(2)	C(4)-C(5)-C(20)-C(25)	122.07(19)
C(9)-C(5)-C(20)-C(25)	-122.97(18)	C(6)-C(5)-C(20)-C(21)	178.40(17)
C(4)-C(5)-C(20)-C(21)	-57.7(2)	C(9)-C(5)-C(20)-C(21)	57.2(2)
C(25)-C(20)-C(21)-C(22)	0.5(3)	C(5)-C(20)-C(21)-C(22)	-179.67(17)
C(20)-C(21)-C(22)-C(23)	1.0(3)	C(21)-C(22)-C(23)-C(24)	-1.7(3)
C(22)-C(23)-C(24)-O(24)	-177.2(2)	C(22)-C(23)-C(24)-C(25)	0.9(3)
C(21)-C(20)-C(25)-C(24)	-1.3(3)	C(5)-C(20)-C(25)-C(24)	178.89(18)
O(24)-C(24)-C(25)-C(20)	178.8(2)	C(23)-C(24)-C(25)-C(20)	0.6(3)

Table S7. Hydrogen bonds for DC-01-00-76.1 [Å and °].

D-H...A	d(D-H)	d(H...A)	d(D...A)	<(DHA)
N(2)-H(2)...Cl(26)#1	0.93(3)	2.20(3)	3.0954(19)	163(2)
C(12)-H(12B)...Cl(26)#2	0.97	2.98	3.860(2)	151.9
C(13)-H(13A)...Cl(26)#1	0.97	2.98	3.691(3)	130.8
O(24)-H(24)...Cl(26)#3	0.88(4)	2.22(4)	3.0638(19)	159(4)

Symmetry transformations used to generate equivalent atoms:
#1 -x+1,y-1/2,-z+1 #2 x,y,z+1 #3 x-1,y,z

Table S1. Crystal data and structure refinement for Compound **20.**

Identification code	knih136	
Empirical formula	$C_{25}H_{32}ClNO$	
Formula weight	397.96	
Temperature	293(2) K	
Wavelength	1.54178 Å	
Crystal system	Monoclinic	
Space group	$P2_1$	
Unit cell dimensions	a = 10.847(2) Å	〈= 90°.
	b = 7.4852(15) Å	®= 107.022(7)°.
	c = 13.670(2) Å	© = 90°.
Volume	1061.3(4) Å^3	
Z	2	
Density (calculated)	1.245 Mg/m^3	
Absorption coefficient	1.693 mm^{-1}	
F(000)	428	
Crystal size	0.174 x 0.063 x 0.060 mm^3	
Theta range for data collection	3.381 to 74.381°.	
Index ranges	-13<=h<=13, -9<=k<=9, -17<=l<=16	
Reflections collected	18404	
Independent reflections	4315 [R_{int} = 0.0263]	
Completeness to theta = 67.679°	99.9 %	
Absorption correction	Semi-empirical from equivalents	
Max. and min. transmission	0.7538 and 0.6568	
Refinement method	Full-matrix least-squares on F^2	
Data / restraints / parameters	4315 / 1 / 261	
Goodness-of-fit on F^2	1.010	
Final R indices [I>2sigma(I)]	R_1 = 0.0273, wR_2 = 0.0709	
R indices (all data)	R_1 = 0.0294, wR_2 = 0.0727	
Absolute structure parameter	0.040(5)	
Largest diff. peak and hole	0.243 and -0.183 e.Å^{-3}	

Table S2. Atomic coordinates (x 10^4) and equivalent isotropic displacement parameters (Å^{2}x 10^3) for DC-01-0155. U(eq) is defined as one third of the trace of the orthogonalized U^{ij} tensor.

	x	y	z	U(eq)
C(1)	5486(2)	6025(2)	2802(1)	28(1)
N(2)	6355(2)	5356(2)	2187(1)	29(1)
C(3)	5693(2)	5323(3)	1056(1)	37(1)
C(4)	4381(2)	4412(3)	799(1)	36(1)
C(5)	3499(2)	5001(3)	1445(1)	29(1)
C(7)	4105(2)	8390(3)	1579(2)	43(1)
C(6)	3049(2)	6969(3)	1236(2)	39(1)
C(8)	5097(2)	7983(3)	2611(2)	37(1)
C(9)	4279(2)	4843(2)	2596(1)	27(1)
C(10)	4590(2)	2959(3)	2981(1)	31(1)
C(11)	4280(2)	2233(3)	3757(2)	43(1)
C(12)	3530(3)	3051(4)	4396(2)	63(1)
C(13)	2300(2)	3804(3)	1234(1)	33(1)
C(14)	1522(2)	3925(3)	1882(2)	38(1)
C(15)	457(2)	2822(3)	1763(2)	42(1)
O(16)	-211(2)	3011(3)	2462(2)	68(1)
C(17)	132(2)	1589(3)	975(2)	50(1)
C(18)	874(2)	1502(4)	309(2)	58(1)
C(19)	1948(2)	2590(3)	434(2)	48(1)
C(20)	7629(2)	6326(3)	2406(1)	34(1)
C(21)	8378(2)	6283(4)	3527(2)	49(1)
C(22)	9760(2)	6927(3)	3782(2)	39(1)
C(23)	10193(2)	8073(4)	3159(2)	49(1)
C(24)	11467(2)	8668(4)	3452(2)	56(1)
C(25)	12301(2)	8122(4)	4359(2)	56(1)
C(26)	11881(2)	6994(4)	4990(2)	57(1)
C(27)	10619(2)	6399(4)	4701(2)	50(1)
Cl(28)	-2869(1)	1341(1)	1922(1)	46(1)

Table S3. Bond lengths [Å] and angles [°] for DC-01-0155.

C(1)-N(2)	1.520(2)	C(1)-C(8)	1.527(3)
C(1)-C(9)	1.536(2)	C(1)-H(1)	0.9800
N(2)-C(3)	1.503(2)	N(2)-C(20)	1.512(2)
N(2)-H(2)	0.88(3)	C(3)-C(4)	1.523(3)
C(3)-H(3A)	0.9700	C(3)-H(3B)	0.9700
C(4)-C(5)	1.545(3)	C(4)-H(4A)	0.9700
C(4)-H(4B)	0.9700	C(5)-C(13)	1.535(3)
C(5)-C(6)	1.552(3)	C(5)-C(9)	1.557(2)
C(7)-C(6)	1.532(3)	C(7)-C(8)	1.533(3)
C(7)-H(7A)	0.9700	C(7)-H(7B)	0.9700
C(6)-H(6A)	0.9700	C(6)-H(6B)	0.9700
C(8)-H(8A)	0.9700	C(8)-H(8B)	0.9700
C(9)-C(10)	1.508(3)	C(9)-H(9)	0.9800
C(10)-C(11)	1.320(3)	C(10)-H(10)	0.9300
C(11)-C(12)	1.488(4)	C(11)-H(11)	0.9300
C(12)-H(12A)	0.9600	C(12)-H(12B)	0.9600
C(12)-H(12C)	0.9600	C(13)-C(19)	1.387(3)
C(13)-C(14)	1.394(3)	C(15)-O(16)	1.365(3)
C(15)-C(17)	1.384(3)	C(15)-C(14)	1.390(3)
O(16)-H(16)	0.89(4)	C(17)-C(18)	1.382(4)
C(17)-H(17)	0.9300	C(18)-C(19)	1.390(3)
C(18)-H(18)	0.9300	C(19)-H(19)	0.9300
C(20)-C(21)	1.510(3)	C(20)-H(20A)	0.9700
C(20)-H(20B)	0.9700	C(21)-C(22)	1.515(3)
C(21)-H(21A)	0.9700	C(21)-H(21B)	0.9700
C(22)-C(23)	1.384(3)	C(22)-C(27)	1.385(3)
C(23)-C(24)	1.395(3)	C(23)-H(23)	0.9300
C(24)-C(25)	1.366(4)	C(24)-H(24)	0.9300
C(25)-C(26)	1.377(4)	C(25)-H(25)	0.9300
C(26)-C(27)	1.383(3)	C(26)-H(26)	0.9300
C(27)-H(27)	0.9300	C(14)-H(14)	0.9300
N(2)-C(1)-C(8)	113.99(14)	N(2)-C(1)-C(9)	110.04(13)
C(8)-C(1)-C(9)	110.13(15)	N(2)-C(1)-H(1)	107.5
C(8)-C(1)-H(1)	107.5	C(9)-C(1)-H(1)	107.5
C(3)-N(2)-C(20)	110.24(14)	C(3)-N(2)-C(1)	112.76(14)
C(20)-N(2)-C(1)	114.33(14)	C(3)-N(2)-H(2)	103.5(15)
C(20)-N(2)-H(2)	104.6(16)	C(1)-N(2)-H(2)	110.5(15)
N(2)-C(3)-C(4)	112.21(15)	N(2)-C(3)-H(3A)	109.2
C(4)-C(3)-H(3A)	109.2	N(2)-C(3)-H(3B)	109.2
C(4)-C(3)-H(3B)	109.2	H(3A)-C(3)-H(3B)	107.9
C(3)-C(4)-C(5)	115.70(16)	C(3)-C(4)-H(4A)	108.4
C(5)-C(4)-H(4A)	108.4	C(3)-C(4)-H(4B)	108.4
C(5)-C(4)-H(4B)	108.4	H(4A)-C(4)-H(4B)	107.4

Table S3. (continued).

C(13)-C(5)-C(4)	111.01(15)	C(13)-C(5)-C(6)	108.44(15)
C(4)-C(5)-C(6)	112.28(16)	C(13)-C(5)-C(9)	109.00(14
C(4)-C(5)-C(9)	108.15(15)	C(6)-C(5)-C(9)	107.86(14)
C(6)-C(7)-C(8)	113.81(16)	C(6)-C(7)-H(7A)	108.8
C(8)-C(7)-H(7A)	108.8	C(6)-C(7)-H(7B)	108.8
C(8)-C(7)-H(7B)	108.8	H(7A)-C(7)-H(7B)	107.7
C(7)-C(6)-C(5)	115.88(17)	C(7)-C(6)-H(6A)	108.3
C(5)-C(6)-H(6A)	108.3	C(7)-C(6)-H(6B)	108.3
C(5)-C(6)-H(6B)	108.3	H(6A)-C(6)-H(6B)	107.4
C(1)-C(8)-C(7)	115.61(15)	C(1)-C(8)-H(8A)	108.4
C(7)-C(8)-H(8A)	108.4	C(1)-C(8)-H(8B)	108.4
C(7)-C(8)-H(8B)	108.4	H(8A)-C(8)-H(8B)	107.4
C(10)-C(9)-C(1)	112.52(15)	C(10)-C(9)-C(5)	115.05(14)
C(1)-C(9)-C(5)	108.86(14)	C(10)-C(9)-H(9)	106.6
C(1)-C(9)-H(9)	106.6	C(5)-C(9)-H(9)	106.6
C(11)-C(10)-C(9)	125.53(18)	C(11)-C(10)-H(10)	117.2
C(9)-C(10)-H(10)	117.2	C(10)-C(11)-C(12)	127.9(2)
C(10)-C(11)-H(11)	116.0	C(12)-C(11)-H(11)	116.0
C(11)-C(12)-H(12A)	109.5	C(11)-C(12)-H(12B)	109.5
H(12A)-C(12)-H(12B)	109.5	C(11)-C(12)-H(12C)	109.5
H(12A)-C(12)-H(12C)	109.5	H(12B)-C(12)-H(12C)	109.5
C(19)-C(13)-C(14)	117.74(19)	C(19)-C(13)-C(5)	123.57(18)
C(14)-C(13)-C(5)	118.69(17)	O(16)-C(15)-C(17)	123.3(2)
O(16)-C(15)-C(14)	116.7(2)	C(17)-C(15)-C(14)	120.0(2)
C(15)-O(16)-H(16)	108(3)	C(18)-C(17)-C(15)	118.7(2)
C(18)-C(17)-H(17)	120.7	C(15)-C(17)-H(17)	120.7
C(17)-C(18)-C(19)	121.3(2)	C(17)-C(18)-H(18)	119.3
C(19)-C(18)-H(18)	119.3	C(13)-C(19)-C(18)	120.5(2)
C(13)-C(19)-H(19)	119.7	C(18)-C(19)-H(19)	119.7
C(21)-C(20)-N(2)	112.30(15)	C(21)-C(20)-H(20A)	109.1
N(2)-C(20)-H(20A)	109.1	C(21)-C(20)-H(20B)	109.1
N(2)-C(20)-H(20B)	109.1	H(20A)-C(20)-H(20B)	107.9
C(20)-C(21)-C(22)	115.38(17)	C(20)-C(21)-H(21A)	108.4
C(22)-C(21)-H(21A)	108.4	C(20)-C(21)-H(21B)	108.4
C(22)-C(21)-H(21B)	108.4	H(21A)-C(21)-H(21B)	107.5
C(23)-C(22)-C(27)	118.4(2)	C(23)-C(22)-C(21)	123.2(2)
C(27)-C(22)-C(21)	118.4(2)	C(22)-C(23)-C(24)	120.5(2)
C(22)-C(23)-H(23)	119.7	C(24)-C(23)-H(23)	119.7
C(25)-C(24)-C(23)	120.1(2)	C(25)-C(24)-H(24)	119.9
C(23)-C(24)-H(24)	119.9	C(24)-C(25)-C(26)	120.0(2)
C(24)-C(25)-H(25)	120.0	C(26)-C(25)-H(25)	120.
C(25)-C(26)-C(27)	120.0(2)	C(25)-C(26)-H(26)	120.0
C(27)-C(26)-H(26)	120.0	C(26)-C(27)-C(22)	120.9(2)

C(26)-C(27)-H(27)	119.5	C(22)-C(27)-H(27)	119.5
C(15)-C(14)-C(13)	121.66(19)	C(15)-C(14)-H(14)	119.2
C(13)-C(14)-H(14)	119.2		

Table S4. Anisotropic displacement parameters ($Å^2 x 10^3$) for DC-01-0155. The anisotropic displacement factor exponent takes the form: $-2□^2[h^2 a^{*2} U^{11} + ... + 2 h k a^* b^* U^{12}]$

	U^{11}	U^{22}	U^{33}	U^{23}	U^{13}	U^{12}
C(1)	29(1)	31(1)	27(1)	-3(1)	10(1)	-6(1)
N(2)	29(1)	29(1)	31(1)	0(1)	11(1)	-4(1)
C(3)	39(1)	46(1)	29(1)	-2(1)	14(1)	-5(1)
C(4)	37(1)	43(1)	27(1)	-5(1)	9(1)	-4(1)
C(5)	27(1)	30(1)	28(1)	-1(1)	5(1)	-2(1)
C(7)	47(1)	27(1)	56(1)	6(1)	15(1)	-1(1)
C(6)	37(1)	34(1)	42(1)	5(1)	5(1)	1(1)
C(8)	37(1)	28(1)	46(1)	-7(1)	14(1)	-5(1)
C(9)	27(1)	27(1)	28(1)	-1(1)	10(1)	-4(1)
C(10)	29(1)	30(1)	36(1)	-1(1)	10(1)	-1(1)
C(11)	50(1)	35(1)	42(1)	6(1)	12(1)	-3(1)
C(12)	95(2)	58(2)	48(1)	7(1)	39(1)	0(2)
C(13)	28(1)	31(1)	35(1)	0(1)	2(1)	-1(1)
C(15)	28(1)	41(1)	56(1)	1(1)	9(1)	-2(1)
O(16)	47(1)	74(1)	91(1)	-22(1)	35(1)	-25(1)
C(17)	34(1)	42(1)	66(1)	-1(1)	2(1)	-12(1)
C(18)	50(1)	56(1)	61(1)	-25(1)	6(1)	-16(1)
C(19)	43(1)	53(1)	46(1)	-15(1)	9(1)	-11(1)
C(20)	30(1)	35(1)	40(1)	-1(1)	14(1)	-7(1)
C(21)	37(1)	69(1)	41(1)	4(1)	10(1)	-18(1)
C(22)	34(1)	41(1)	43(1)	-3(1)	10(1)	-7(1)
C(23)	39(1)	56(1)	51(1)	4(1)	9(1)	-12(1)
C(24)	43(1)	59(2)	69(2)	-1(1)	19(1)	-15(1)
C(25)	34(1)	58(1)	73(2)	-14(1)	11(1)	-9(1)
C(26)	42(1)	61(2)	58(1)	-5(1)	-2(1)	2(1)
C(27)	45(1)	50(1)	51(1)	3(1)	8(1)	-4(1)
Cl(28)	38(1)	36(1)	71(1)	-3(1)	25(1)	-4(1)
C(14)	30(1)	36(1)	47(1)	-5(1)	8(1)	-4(1)

Table S5. Hydrogen coordinates (x 10^4) and isotropic displacement parameters ($Å^2$x 10^3) for DC-01-0155.

	x	y	z	U(eq)
H(1)	5962	5902	3528	34
H(2)	6570(20)	4230(40)	2334(18)	35
H(3A)	6233	4697	715	44
H(3B)	5582	6539	799	44
H(4A)	4517	3133	880	43
H(4B)	3932	4635	84	43
H(7A)	3704	9532	1625	52
H(7B)	4551	8498	1061	52
H(6A)	2643	7108	507	47
H(6B)	2397	7200	1577	47
H(8A)	5867	8680	2660	44
H(8B)	4750	8383	3151	44
H(9)	3740	5358	2987	33
H(10)	5039	2248	2643	38
H(11)	4564	1070	3924	51
H(12A)	3487	4321	4294	95
H(12B)	3945	2792	5104	95
H(12C)	2673	2565	4201	95
H(16)	-920(40)	2350(60)	2260(30)	90(12)
H(17)	-571	833	895	60
H(18)	650	701	-235	69
H(19)	2435	2502	-22	57
H(20A)	8143	5780	2013	41
H(20B)	7472	7559	2187	41
H(21A)	8381	5065	3771	59
H(21B)	7927	7012	3899	59
H(23)	9629	8450	2540	59
H(24)	11750	9438	3028	67
H(25)	13153	8512	4550	67
H(26)	12446	6631	5610	69
H(27)	10343	5633	5130	60
H(14)	1720	4764	2406	46

Table S6. Torsion angles [°] for DC-01-0155.

C(8)-C(1)-N(2)-C(3)	-66.02(19)	C(9)-C(1)-N(2)-C(3)	58.26(19)
C(8)-C(1)-N(2)-C(20)	60.9(2)	C(9)-C(1)-N(2)-C(20)	-174.77(15)
C(20)-N(2)-C(3)-C(4)	-178.09(17)	C(1)-N(2)-C(3)-C(4)	-49.0(2)
N(2)-C(3)-C(4)-C(5)	47.1(2)	C(3)-C(4)-C(5)-C(13)	-171.38(17)
C(3)-C(4)-C(5)-C(6)	67.0(2)	C(3)-C(4)-C(5)-C(9)	-51.8(2)
C(8)-C(7)-C(6)-C(5)	-39.8(3)	C(13)-C(5)-C(6)-C(7)	169.96(17)
C(4)-C(5)-C(6)-C(7)	-67.0(2)	C(9)-C(5)-C(6)-C(7)	52.1(2)
N(2)-C(1)-C(8)-C(7)	73.3(2)	C(9)-C(1)-C(8)-C(7)	-50.9(2)
C(6)-C(7)-C(8)-C(1)	38.6(3)	N(2)-C(1)-C(9)-C(10)	65.64(18)
C(8)-C(1)-C(9)-C(10)	-167.87(14)	N(2)-C(1)-C(9)-C(5)	-63.10(18)
C(8)-C(1)-C(9)-C(5)	63.38(18)	C(13)-C(5)-C(9)-C(10)	52.0(2)
C(4)-C(5)-C(9)-C(10)	-68.76(19)	C(6)-C(5)-C(9)-C(10)	169.58(16)
C(13)-C(5)-C(9)-C(1)	179.36(15)	C(4)-C(5)-C(9)-C(1)	58.57(19)
C(6)-C(5)-C(9)-C(1)	-63.10(18)	C(1)-C(9)-C(10)-C(11)	111.2(2)
C(5)-C(9)-C(10)-C(11)	-123.4(2)	C(9)-C(10)-C(11)-C(12)	2.4(4)
C(4)-C(5)-C(13)-C(19)	-11.2(3)	C(6)-C(5)-C(13)-C(19)	112.6(2)
C(9)-C(5)-C(13)-C(19)	-130.3(2)	C(4)-C(5)-C(13)-C(14)	168.24(17)
C(6)-C(5)-C(13)-C(14)	-68.0(2)	C(9)-C(5)-C(13)-C(14)	49.2(2)
O(16)-C(15)-C(17)-C(18)	-179.9(2)	C(14)-C(15)-C(17)-C(18)	-0.9(3)
C(15)-C(17)-C(18)-C(19)	1.7(4)	C(14)-C(13)-C(19)-C(18)	-1.8(3)
C(5)-C(13)-C(19)-C(18)	177.7(2)	C(17)-C(18)-C(19)-C(13)	-0.4(4)
C(3)-N(2)-C(20)-C(21)	-175.9(2)	C(1)-N(2)-C(20)-C(21)	55.8(2)
N(2)-C(20)-C(21)-C(22)	169.4(2)	C(20)-C(21)-C(22)-C(23)	23.5(4)
C(20)-C(21)-C(22)-C(27)	-159.0(2)	C(27)-C(22)-C(23)-C(24)	0.4(4)
C(21)-C(22)-C(23)-C(24)	177.8(3)	C(22)-C(23)-C(24)-C(25)	0.0(4)
C(23)-C(24)-C(25)-C(26)	-0.5(4)	C(24)-C(25)-C(26)-C(27)	0.7(4)
C(25)-C(26)-C(27)-C(22)	-0.3(4)	C(23)-C(22)-C(27)-C(26)	-0.2(4)
C(21)-C(22)-C(27)-C(26)	-177.8(3)	O(16)-C(15)-C(14)-C(13)	177.7(2)
C(17)-C(15)-C(14)-C(13)	-1.2(3)	C(19)-C(13)-C(14)-C(15)	2.6(3)
C(5)-C(13)-C(14)-C(15)	-176.94(18)		

Table S7. Hydrogen bonds for DC-01-0155 [Å and °].

D-H...A	d(D-H)	d(H...A)	d(D...A)	<(DHA)
N(2)-H(2)...Cl(28)#1	0.88(3)	2.36(3)	3.1700(18)	153(2)
C(4)-H(4B)...Cl(28)#2	0.97	2.94	3.877(2)	163.6
C(8)-H(8A)...Cl(28)#3	0.97	2.77	3.645(2)	150.3
O(16)-H(16)...Cl(28)	0.89(4)	2.16(4)	3.0289(19)	165(4)
C(20)-H(20B)...Cl(28)#3	0.97	2.86	3.823(2)	169.7

Symmetry transformations used to generate equivalent atoms:
#1 x+1,y,z   #2 -x,y+1/2,-z   #3 x+1,y+1,z
